# Incorporation of sensing modalities into de novo designed fluorescence-activating proteins

**DOI:** 10.1038/s41467-020-18911-w

**Published:** 2021-02-08

**Authors:** Jason C. Klima, Lindsey A. Doyle, Justin Daho Lee, Michael Rappleye, Lauren A. Gagnon, Min Yen Lee, Emilia P. Barros, Anastassia A. Vorobieva, Jiayi Dou, Samantha Bremner, Jacob S. Quon, Cameron M. Chow, Lauren Carter, David L. Mack, Rommie E. Amaro, Joshua C. Vaughan, Andre Berndt, Barry L. Stoddard, David Baker

**Affiliations:** 1 Department of Biochemistry, University of Washington, Seattle, WA USA; 2 Institute for Protein Design, University of Washington, Seattle, WA USA; 3 Division of Basic Sciences, Fred Hutchinson Cancer Research Center, Seattle, WA USA; 4 Molecular Engineering Ph.D. Program, University of Washington, Seattle, WA USA; 5 Institute for Stem Cell and Regenerative Medicine, University of Washington, Seattle, WA USA; 6 Department of Bioengineering, University of Washington, Seattle, WA USA; 7 Department of Chemistry, University of Washington, Seattle, WA USA; 8 Department of Chemistry and Biochemistry, University of California, San Diego, La Jolla, CA USA; 9 Department of Rehabilitation Medicine, University of Washington, Seattle, WA USA; 10 Department of Physiology and Biophysics, University of Washington, Seattle, WA USA; 11 Howard Hughes Medical Institute, University of Washington, Seattle, WA USA; 12 Present Address: Department of Bioengineering, Stanford University, Stanford, CA USA

**Keywords:** Wide-field fluorescence microscopy, Fluorescent proteins, Protein design, X-ray crystallography

## Abstract

Through the efforts of many groups, a wide range of fluorescent protein reporters and sensors based on green fluorescent protein and its relatives have been engineered in recent years. Here we explore the incorporation of sensing modalities into de novo designed fluorescence-activating proteins, called mini-fluorescence-activating proteins (mFAPs), that bind and stabilize the fluorescent *cis*-planar state of the fluorogenic compound DFHBI. We show through further design that the fluorescence intensity and specificity of mFAPs for different chromophores can be tuned, and the fluorescence made sensitive to pH and Ca^2+^ for real-time fluorescence reporting. Bipartite split mFAPs enable real-time monitoring of protein–protein association and (unlike widely used split GFP reporter systems) are fully reversible, allowing direct readout of association and dissociation events. The relative ease with which sensing modalities can be incorporated and advantages in smaller size and photostability make de novo designed fluorescence-activating proteins attractive candidates for optical sensor engineering.

## Abstract (web summary)

Fluorescent protein reporters based on GFP exist, but have intrinsic disadvantages. Here the authors incorporate pH, Ca^2+^ and protein–protein interaction sensing modalities into de novo designed mini-fluorescence-activating proteins (mFAPs), with increased photostability and smaller size, which bind a range of DFHBI chromophore variants.

## Introduction

**Fig. 1:**
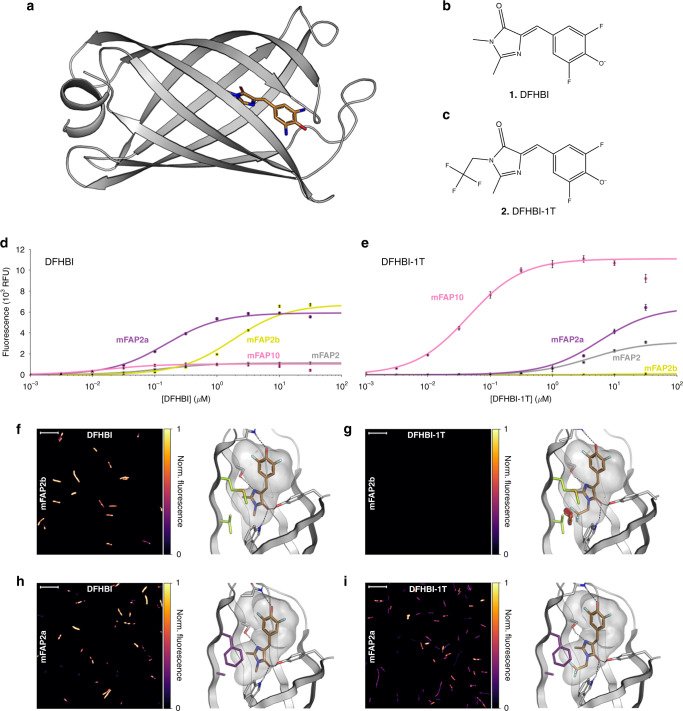
Characterization of brighter and chromophore-specific mFAPs. **a** Computational model of de novo designed β-barrel variant mFAP2b showing protein backbone *(cartoon)* and bound DFHBI chromophore *(sticks)*. **b**, **c** Chemical structures of DFHBI and DFHBI-1T, respectively. **d**, **e** In vitro titration of **d** DFHBI or **e** DFHBI-1T with mFAP2 *(gray)*, mFAP2b *(lime)*, mFAP2a *(violet)*, and mFAP10 *(pink)* proteins. Error bars represent s.d. of the mean of eight technical replicates. Normalized means were fit to a single-binding site isotherm function using non-linear least squares fitting to obtain *K*_d_ values (Table 1), and the fits scaled to the maximum mean relative fluorescence unit (RFU) values *(lines)*. **f**–**i** Each panel shows a representative image of the fluorescence signal emitted by *E. coli* cells expressing the indicated mFAP variant labeled with 10.0 µM concentration of the indicated chromophore *(left)* and a zoom-in of the modeled-binding pocket of that mFAP variant bound to the chromophore *(right)*. The images *(left)* are the pseudocolored normalized fluorescence intensity per pixel. Scale bars represent 10 microns. Imaging was independently repeated twice with similar results. The computational models *(right)* show the residues unique to mFAP2b (V13, M15) *(lime sticks)* or mFAP2a (A13, F15) *(violet sticks)*. Intermolecular hydrogen bonds to the chromophore are shown as black dotted lines. Vacuum electrostatic contact potential around the chromophore is shown in a transparent gray surface. **f** mFAP2b with DFHBI, **g** mFAP2b with DFHBI-1T does not emit a detectable fluorescence signal because binding is precluded by steric clashes of DFHBI-1T with V13 *(red cylinders)*, **h** mFAP2a with DFHBI, and **i** mFAP2a with DFHBI-1T. Source data is available for Fig. 1f–i.

De novo designed mini-fluorescence-activating proteins (mFAPs) (Fig. 1a) bind and activate the fluorescence of the fluorogenic compound DFHBI (3,5-difluoro-4-hydroxybenzylidene imidazolinone) (**1**, Fig. 1b) in vitro and in bacteria, yeast, and mammalian cells^1^. DFHBI does not fluoresce when free in solution^2^, but becomes brightly fluorescent upon stabilization of the *cis*-planar conformation (planar Z conformation) through macromolecular binding^3^. RNA aptamers have been evolved to bind similar fluorogenic DFHBI-derived compounds^4,5^ (e.g., DFHBI, DFHBI-1T [(Z)-4-(3,5-difluoro-4-hydroxybenzylidene)-2-methyl-1-(2,2,2-trifluoroethyl)-1H-imidazol-5(4 H)-one] (**2**, Fig. 1c) and DFHO [3,5-difluoro-4-hydroxybenzylidene imidazolinone-2-oxime]), with up to 0.72 fluorescence quantum yield^6^, but to our knowledge so far no protein-based systems other than the mFAPs have been reported to bind and fluorescently activate DFHBI-1T or DFHO chromophores.

Circularly permuted fluorescent proteins such as cpGFP and cpFAST enable the real-time detection of analytes of interest using fluorescence microscopy^7–9^. Likewise, self-complementing split fluorescent protein reporter systems based on green fluorescent protein (GFP) variants^10–12^ and FAST^13^ have been engineered to monitor protein–protein interactions in vitro, *in cyto*, and in vivo^14^ using fluorescence microscopy. The β-barrel structure of mFAPs suggests that mFAPs could be re-designed to monitor analyte fluxes and protein–protein interactions; such sensors could have complementary biophysical properties to existing fluorescent proteins (such as intrinsically fluorescent GFP and extrinsically fluorogenic DiBs^15^, Y-FAST^16^, Ca^2+^-responsive cpFAST^7^, and splitFAST^13^ reporters and sensors).

mFAPs have several biophysical properties that make them attractive candidates for further development. First, they are less than half the size of GFP, so their genetic footprint is smaller, and fusions to proteins of interest are less perturbative. Second, the bound chromophore can readily exchange with free chromophore in solution, and hence mFAPs can be more photostable^17^ than GFP. Third, chemical derivatives of DFHBI with different fluorescence properties can be fluorescently activated, providing more control over color (i.e., fluorescence excitation and emission wavelength) than intrinsically fluorescent proteins^7^. Fourth, de novo mFAPs can be engineered to remain folded at low pH, facilitating the engineering of pH-responsive fluorogenic optical sensors. Finally, the chromophore-binding pocket is close to the protein surface, potentially enabling the design of allosteric coupling between chromophore binding and linked analyte binding domains for analyte-responsive fluorogenic optical sensors^7,18,19^.

Here, we explore the incorporation of sensing modalities into the mFAPs. We develop and apply methodologies for engineering chromophore-selective, pH-responsive, Ca^2+^-responsive, bipartite, and circularly permuted optical sensors based on de novo designed fluorescence-activating proteins.

## Results

### Optimizing brightness and chromophore selectivity

We began by seeking to improve the stability of mFAPs at low pH, the binding affinity to the phenolic and phenolate forms of DFHBI, as well as the fluorescence intensity of both complexes. mFAP2 was chosen for optimization because it has the highest absolute fluorescence quantum yield (*ϕ*_c_ of 2.1%) and highest affinity (*K*_d_ of ~180 nM) for the phenolate form of DFHBI compared to mFAP1^1^. Through a series of library selections (see “Methods”) targeting aliphatic and aromatic residues directly interacting with DFHBI or in the hydrophobic core of the β-barrel, as well as residues in the loop connecting the seventh and eighth β-strands (loop7) of the β-barrel, we obtained three brighter and chromophore-selective mFAP2 variants: mFAP2a, mFAP2b, and mFAP10 that incorporate 12, 10, and 11 mutations, respectively (Supplementary Fig. 1).

Titrations of mFAP2, mFAP2a, mFAP2b, and mFAP10 with either DFHBI (Fig. 1d) or DFHBI-1T (Fig. 1e) and quantum yield measurements (Table 1 and Supplementary Fig. 2) showed that: mFAP2, mFAP2a, and mFAP10 have ~2.7-fold, ~2.5-fold, and ~12-fold brighter fluorescence with DFHBI-1T than DFHBI, but bind DFHBI with ~30-fold, ~39-fold, and ~2.6-fold higher affinity than DFHBI-1T, respectively; and that mFAP2b has ~30-fold brighter fluorescence with DFHBI than DFHBI-1T and binds DFHBI with ~6.1-fold higher affinity than DFHBI-1T. The mFAP10–DFHBI-1T complex is the brightest, with 23.7% absolute quantum yield (under conditions with 99.9% of chromophore bound) and a 17.5-fold increased brightness over the previously reported mFAP2–DFHBI complex^1^, resulting in a 242-fold fluorescence activation over free DFHBI-1T (Table 1). Relative fluorescence intensities and thermodynamic dissociation constants (*K*_d_) for the deprotonated (phenolate) states of DFHBI, DFHBI-1T, and DFHO for the Ca^2+^-independent mFAP variants presented in this study are given in Supplementary Table 1 (for example, mFAP3 binds the yellow colored DFHO chromophore with ~10-fold lower fluorescence intensity than mFAP2b with DFHBI). Using a laser scanning confocal fluorescence microscope to image *E. coli* expressing either mFAP2a or mFAP2b labeled with either DFHBI or DFHBI-1T, we observed pronounced chromophore selectivity of mFAP2b for DFHBI over DFHBI-1T, and chromophore promiscuity of mFAP2a for both DFHBI or DFHBI-1T (Fig. 1f–i). *E. coli* cultures expressing either mFAP2a or mFAP2b mixed in a 1:1 cellular ratio labeled with DFHBI-1T have ~49% of the total fluorescence signal of cultures labeled with DFHBI (Supplementary Fig. 3).

**Table 1:** Photophysical properties of mFAPs with DFHBI and DFHBI-1T compared with controls.

	*λ*_abs_ (nm)^a^	*λ*_ex_ (nm)^a^	*λ*_em_ (nm)^a^	Extinction coefficient (M^−1^ cm^−1^)^b^	Brightness (M^−1^ cm^−1^)^c^	Absolute quantum yield^d^	Relative quantum yield^d^	Reported quantum yield	% Bound	*K*_d_ (μM)
EGFP	–	488^f^	507^f^	56,000^f^	33,600^f^	–	–	0.60^f^	–	–
mFAP2a + DFHBI	491	491	505	64,900	3890	0.060	0.063	–	99.9	0.15 ± 0.011
mFAP2a + DFHBI-1T	492	493	505	75,100	9690	0.129	0.128	–	95.8	5.8 ± 0.86
mFAP2b + DFHBI	495	495	509	60,500	5630	0.093	0.099	–	99.1	1.8 ± 0.25
mFAP2b + DFHBI-1T	430	494	505	37,800	189	0.005	0.003	–	95.1	11 ± 3.1
mFAP10 + DFHBI	470	475	497	48,900	1290	0.026	0.029	–	100.0	0.017 ± 0.0079
mFAP10 + DFHBI-1T	484	485	503	67,200	15,900 (2.1x dimmer than EGFP)	0.237	0.230	–	99.9	0.045 ± 0.0065
DFHBI	418^e^	423^e^	489^e^	30,100^e^ 31,935^f^	–	0.001^f^	–	0.0007^e^	–	–
DFHBI-1T	422^e^	426^e^	495^e^	35,400^e^	–	–	–	0.00098^e^	–	–

The % bound values are calculated based on the reported *K*_d_ values and final protein and chromophore concentrations used in quantum yield measurements. *K*_d_ values are obtained by non-linear least squares fits to the mean fluorescence intensities of the eight technical replicates per chromophore titration (Fig. 1d, e). *K*_d_ error estimates are s.d. of the mean of the non-linear least squares fits.

^a^*λ*_abs_ is peak absorbance wavelength, *λ*_ex_ is peak excitation wavelength, and *λ*_em_ is peak emission wavelength (Supplementary Fig. 2).

^b^Extinction coefficients are measured from *λ*_abs_ estimated based on 1 data point in this study.

^c^Brightness is defined as extinction coefficient multiplied by absolute quantum yield.

^d^Absolute quantum yield is the average of ten scans measured with an integrating sphere; relative quantum yield is reported using Acridine Yellow G^54^ and fluorescein as reference standards.

^e^Previously reported value^4^.

^f^Previously reported value^1^.

**Fig. 2:**
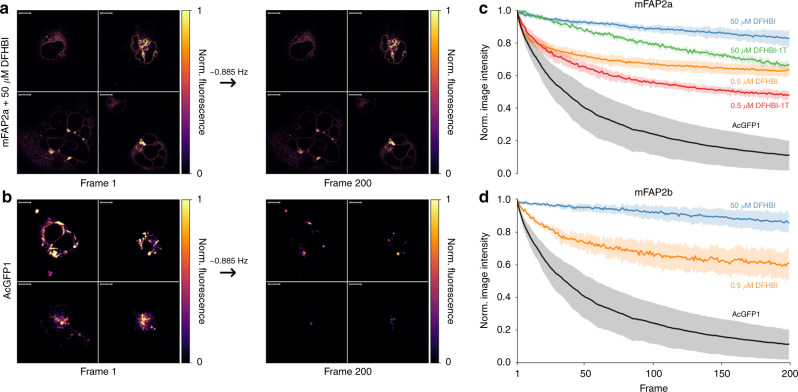
Photostability of mFAPs compared to AcGFP1. **a**, **b** Normalized fluorescence intensity images for four regions of interest (ROIs) acquired under continuous wave imaging at ~0.885 Hz (1.13 s frame^−1^) shown for frame 1 *(left)* and frame 200 *(right)* of fixed COS-7 cells expressing endoplasmic reticulum-targeted **a** mFAP2a labeled with 50.0 µM DFHBI compared to **b** AcGFP1. Scale bars represent 10 microns. **c**, **d** Means *(lines)* and s.d. of the means *(shading)* of the normalized summed pixel intensities of four ROIs (*n* = 4) under continuous wave imaging (~0.885 Hz) for **c** mFAP2a labeled with 50.0 µM DFHBI (~330-fold above *K*_d_) *(blue)*, 500 nM DFHBI (~3.3-fold above *K*_d_) *(orange)*, 50.0 µM DFHBI-1T (~8.6-fold above *K*_d_) *(green)*, or 500 nM DFHBI-1T (~12-fold below *K*_d_) *(red)* compared to AcGFP1 *(black)*, and **d** mFAP2b labeled at 50.0 µM DFHBI (~28-fold above *K*_d_) *(blue)* or 500 nM DFHBI (~3.6-fold below *K*_d_) *(orange)* compared to AcGFP1 *(black)*. Source data is available for Fig. 2.

We next targeted mFAP2a or mFAP2b to the endoplasmic reticulum (ER) of mammalian COS-7 cells using a C-terminal sec61β localization sequence, and observed bright fluorescence of ER under fixed cell (Supplementary Fig. 4) and live cell (Supplementary Movie 1 and Supplementary Movie 2) epifluorescence microscopy after labeling with DFHBI. Under fixed cell imaging, following washing and re-labeling with DFHBI-1T (Supplementary Fig. 4) the fluorescence was altered as expected, demonstrating external spatiotemporal control over fluorescence. To compare the photostability of mFAP2a and mFAP2b to a monomeric enhanced GFP (EGFP) variant, we also targeted AcGFP1 to the ER of COS-7 cells. Upon continuous wave illumination imaging at ~0.885 Hz (1.13 s frame^−1^) of fixed COS-7 cells using laser scanning confocal fluorescence microscopy, we found at 50.0 µM chromophore (saturating conditions) a 6.2-fold, 3.5-fold, and 6.1-fold improved photostability of mFAP2a–DFHBI, mFAP2a–DFHBI-1T, and mFAP2b–DFHBI complexes over AcGFP1, respectively. At 500 nM chromophore (sub-saturating conditions), the improvements in photostability of the three complexes over AcGFP1 were 5.0-fold, 3.8-fold, and 4.7-fold, respectively (Fig. 2).

### Incorporation of pH-responsiveness

**Fig. 3:**
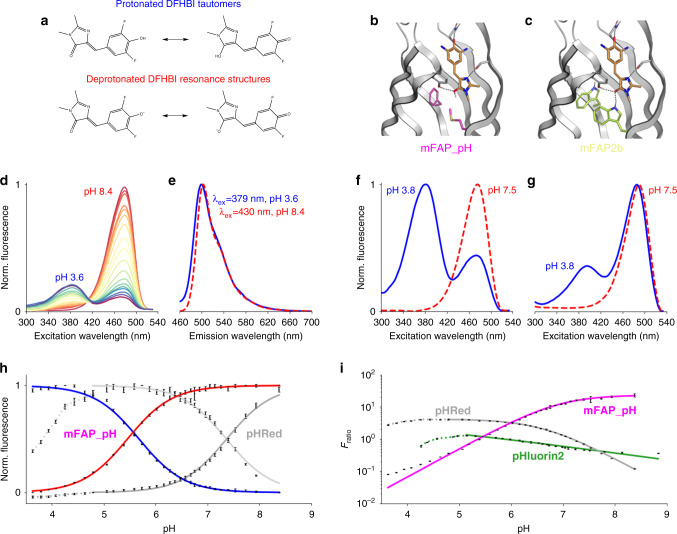
Characterization of pH-responsive mFAP_pH. **a**–**c** Chemical basis of pH-responsiveness in mFAP_pH. **a** Chemical structures of protonated tautomers and deprotonated resonance structures of DFHBI. **b**, **c** Computational models showing the residues unique to **b** mFAP_pH (M27, F93) *(magenta)* and **c** mFAP2b (W27, W93) *(lime)*. The arrangement of intermolecular hydrogen bonds *(black dotted lines)* in **b** the mFAP_pH–DFHBI complex permits binding to both the phenolate and phenolic (shown) forms of DFHBI whereas of **c** the mFAP2b–DFHBI complex only permits binding to the phenolate (shown) form of DFHBI. **d** Normalized fluorescence excitation spectra of the mFAP_pH–DFHBI complex for a pH titration between pH 3.6 and pH 8.4. **e** Normalized fluorescence emission spectra of the mFAP_pH–DFHBI complex at pH 3.6 and pH 8.4. **f**, **g** Fluorescence excitation spectra normalized to pH 3.8 and pH 7.5 of **f** pH-responsive mFAP_pH–DFHBI complex and **g** pH-unresponsive mFAP2b–DFHBI complex. **h** Normalized mean (*n* = 3) fluorescence intensity from the pH titration of the mFAP_pH–DFHBI complex *(blue and red)* and previously reported pHRed *(dark gray and light gray)*, showing fluorescence emission by exciting the blueshifted fluorescence excitation peak *(blue and dark gray)* and fluorescence emission by exciting the redshifted fluorescence excitation peak *(red and light gray)*. **i** Ratiometric fluorescence (*F*_ratio_) from the pH titration of the mFAP_pH–DFHBI complex *(magenta)*, pHRed *(gray)*, and pHluorin2 *(green)*. **h**, **i** The means are fit to **h** a sigmoid or inverse sigmoid function or **i** a logistic function using non-linear least squares fitting *(lines)*. The dotted lines indicate pH values at which the measured *F*_ratio_ coincides with two different pH values, and therefore are not used in the fittings. Error bars represent the s.d. of the mean of three technical replicates.

A prerequisite for designing a robust pH-responsive mFAP is the ability to bind both protonated (phenolic) DFHBI tautomers and both deprotonated (phenolate) DFHBI resonance structures (Fig. 3a). When stabilized in the *cis*-planar conformation, the phenolic and phenolate forms of DFHBI exhibit blueshifted and redshifted peak excitation wavelengths, respectively^6^ (Fig. 3d). mFAP2b binds both forms of DFHBI (Fig. 3g); to increase pH sensitivity we screened design variants based on the change in fluorescence between pH 3.61 and pH 7.34 (Supplementary Fig. 5), and identified a particularly pH-responsive variant we call mFAP_pH. Computational models (Fig. 3b, c) suggest that the amino acid substitutions in mFAP_pH compared to mFAP2b (i.e., W27M and W93F) improve pH-responsiveness of the protein–DFHBI complex by increasing shape complementarity toward the protonated (phenolic) form of DFHBI by removing a hydrogen bond between the W27 indole ring and the DFHBI imidazolinone moiety, and by reducing net positive charge in the β-barrel core via removing a buried unsatisfied hydrogen bond donor in the indole ring of W93, resulting in a higher ratio of protein-bound phenolic DFHBI to phenolate DFHBI at low pH (Fig. 3f, g). The phenolic and phenolate forms of DFHBI had nearly equivalent affinities (*K*_d_ values) for mFAP_pH of ~190 nM and ~160 nM, respectively (Supplementary Fig. 5). The pK_a_ of free, unbound DFHBI in solution^5^ is ~5.4, and we observe the same pK_a_ for the mFAP_pH–DFHBI complex (Fig. 3h).

At peak excitation and emission wavelengths (Fig. 3d, e), mFAP_pH showed a marked ~250-fold-change in ratiometric fluorescence (*F*_ratio_, see “Methods”) from pH 8.38 to pH 3.63, compared with only a ~34-fold-change in *F*_ratio_ from pH 8.38 to pH 4.79 for pHRed^20^ and a ~3.5-fold-change in *F*_ratio_ from pH 8.83 to pH 5.14 for pHluorin2^21^ (Fig. 3i). At low pH, the β-barrel fold of mFAP_pH is more resistant to denaturation than those of pHRed and pHluorin2, and thus the mFAP_pH–DFHBI complex has a higher dynamic range for ratiometric fluorescence across the physiologically relevant pH range. Ratiometric fluorescence imaging of the mFAP_pH–DFHBI complex hence should enable real-time in situ quantification of pH.

### Incorporation of Ca^2+^-responsiveness

**Fig. 4:**
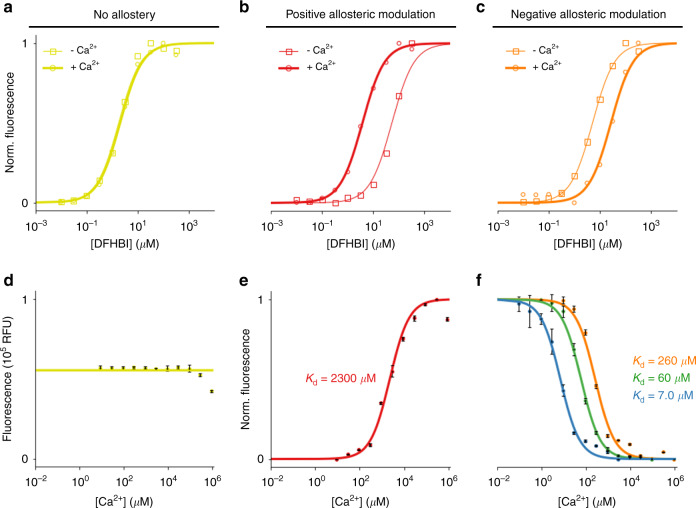
In vitro characterization of Ca^2+^-responsive mFAPs. **a**–**c** DFHBI titration in the absence of Ca^2+^ *(squares)* and presence of Ca^2+^ *(circles)*. **a** For mFAP2b, Ca^2+^ does not affect DFHBI binding. **b** For EF1p2_mFAP2b, binding of Ca^2+^ and DFHBI exhibit positive allostery. **c** For EF1n_mFAP2b, binding of Ca^2+^ and DFHBI exhibit negative allostery. **a**–**c** Normalized fluorescence intensities (*n* = 1) are fit to a sigmoid function using non-linear least squares fitting *(lines)*. **d**–**f** Ca^2+^ titrations with excess DFHBI concentration compared to protein concentration. **d** Unnormalized mean fluorescence intensities of mFAP2b demonstrating a lack of Ca^2+^-responsiveness. **e** Normalized mean fluorescence intensities of EF1p2_mFAP2b (with one EF-hand motif, *K*_d_ = 2300 µM) demonstrating positive allostery between DFHBI and Ca^2+^ binding. **f** Ca^2+^-responsiveness is dependent on the number of EF-hand motifs inserted into loop7, as exemplified by the normalized mean fluorescence intensities of EF1n_mFAP2b (with one EF-hand motif, *K*_d_ = 260 µM), EF2n_mFAP2b (with two EF-hand motifs, *K*_d_ = 60 µM), and EF4n_mFAP2b (with four EF-hand motifs, *K*_d_ = 7.0 µM), demonstrating negative allostery between DFHBI and Ca^2+^ binding. **d**–**f** Error bars represent the s.d. of the mean of three technical replicates. The means (*n* = 3) are fit to a **d** constant function, or **e** sigmoid or **f** inverse sigmoid function with Hill coefficients of 1, using non-linear least squares fitting *(lines)* to obtain *K*_d_ values (Supplementary Table 2).

To enable the engineering of additional environmental responsiveness in mFAPs, we used Rosetta^22,23^ to de novo design 59 extensions of β-barrel loops 1, 3 and 7, and screened them for fluorescence after labeling with DFHBI. We identified five extended loop7 variants that maintain the β-barrel fold and are compatible with DFHBI binding (Supplementary Fig. 6). Using these extended loop sequences as linkers (see “Methods”), we grafted one EF-hand motif^24^, one EF-hand domain^25^ (i.e., two EF-hand motifs), or calmodulin^26^ (i.e., four EF-hand motifs) into loop7 of mFAP2b (Supplementary Fig. 7). Through DFHBI and Ca^2+^ titrations, we found that Ca^2+^ binding was allosterically coupled to DFHBI binding, with either positive^7^ or negative^8^ allosteric modulation of fluorescence (Fig. 4) depending only on the amino acid sequence of the linkers. As expected based on the cooperativity of Ca^2+^ binding to calmodulin^27,28^, we found that as the number of grafted EF-hand motifs increases, so does the affinity for Ca^2+^ ions (Fig. 4f). While some existing fluorescent Ca^2+^ sensors harboring calmodulin such as GCaMP6f^29^ are characterized by Hill coefficients of ~2–3, Ca^2+^-responsive mFAPs are characterized by Hill coefficients of ~1 (similar to previously reported ratiometric-pericam^8^, CatchER^18^, and XCaMPs^30^), suggesting that one Ca^2+^-binding site is allosterically coupled to chromophore affinity (Supplementary Table 2). Circular dichroism experiments showed that Ca^2+^ binding increases the α-helical secondary structure, presumably in the calmodulin domain, and enhances thermostability for EF4n_mFAP2b harboring calmodulin (Supplementary Fig. 8).

Incorporation of the mFAP2a hydrophobic core amino acid substitutions (A13, F15) (Supplementary Fig. 9) increased Ca^2+^ affinity by up to 11.7-fold for positively allosteric proteins and decreased Ca^2+^ affinity by up to 11.6-fold for negatively allosteric proteins (Supplementary Table 2 and Supplementary Figs. 10, 11,  12, 13). The substitutions increase the affinity of DFHBI binding (Fig. 1d), and DFHBI and Ca^2+^ titration data indicate thermodynamic coupling between DFHBI and Ca^2+^ binding (Supplementary Fig. 14 and Supplementary Note 1). Overall, the Ca^2+^-responsive mFAPs exhibit over 500-fold differences in affinity for Ca^2+^ (Supplementary Table 2), enabling the choice of a sensor with optimal fluorescence dynamic range in the anticipated Ca^2+^ concentration range under study.

**Fig. 5:**
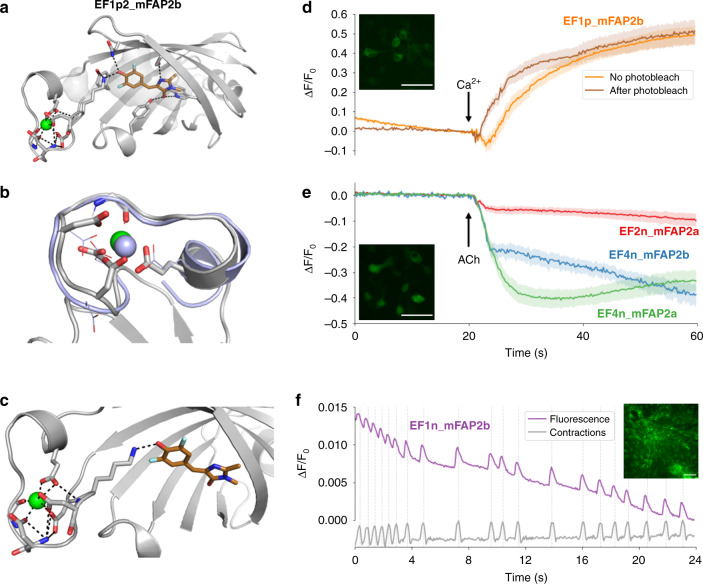
Structural and in cell characterization of Ca^2+^-responsive mFAPs. **a**–**c** X-ray co-crystal structure of EF1p2_mFAP2b bound to DFHBI and Ca^2+^ at 2.1 Å resolution. **a** Structure of monomer with Ca^2+^ *(green sphere)* and DFHBI *(copper sticks)* bound, with protein side-chains *(gray sticks)* forming first- and second-shell hydrogen bonds with Ca^2+^ and DFHBI *(black dotted lines)*, and vacuum electrostatic contact potential around DFHBI *(transparent gray surface)*. **b** Aligned is the nuclear magnetic resonance (NMR) solution structure from PDB accession code 1NKF *(violet cartoon and lines)* with La^3+^ ion bound *(violet sphere)* having the closest C_α_-C_α_ root mean square deviation (2.19 Å) to the structure of the grafted EF-hand motif *(gray sticks and cartoon)* with bound Ca^2+^ ion *(green sphere)*. **c** Co-crystal structure of EF-hand motif residues *(gray sticks)* reveals that residue K101 from the EF-hand motif directly hydrogen bonds *(black dotted lines)* with DFHBI, suggesting an allosteric coupling mechanism between Ca^2+^ *(green sphere)* and DFHBI *(copper sticks)* binding. **d** Fluorescence imaging of Ca^2+^ titrations (beginning at arrow) of live HEK293 cells expressing extracellular EF1p_mFAP2b, demonstrating positive allostery *in cyto*. Average fluorescence fold-change *(lines)* and s.e.m. *(shading)* is shown for regions of interest (ROIs) surrounding single cells without photobleaching *(orange*; *n* = 15*)* or after a photobleaching challenge *(dark orange*; *n* = 15*)* from three technical replicates. **e** Fluorescence imaging of acetylcholine (ACh) stimulations (added at arrow) of live HEK293 cells expressing cytosolic either EF2n_mFAP2a *(red)*, EF4n_mFAP2b *(blue)*, or EF4n_mFAP2a *(green)*, demonstrating negative allostery *in cyto*. Average fluorescence fold-change *(lines)* and s.e.m. *(shading)* are shown for ROIs surrounding single cells expressing EF2n_mFAP2a (*n* = 15), EF4n_mFAP2b (*n* = 10), or EF4n_mFAP2a (*n* = 15) from three technical replicates (Table 2). **f** Fluorescence imaging of live human induced pluripotent stem cell-derived cardiomyocytes expressing sarcoplasmic reticulum-targeted EF1n_mFAP2b *(violet)* labeled with DFHBI at approximately $\frac{{K_{\mathrm{d}}^ - }}{2}$ (Supplementary Table 2) showing whole field of view normalized fluorescence fold-change, demonstrating negative allostery *in cyto*. The normalized average of three ROI traces in the fluorescence channel *(gray)* indicate peak cardiac contraction frames *(dotted lines)*. **d**–**f** Representative whole field of view pseudocolored maximum intensity *z*-axis projections. Scale bars represent **d**, **e** 50 and **f** 100 microns. Source data is available for Fig. 5d–f.

To investigate the origin of the allosteric coupling between Ca^2+^ and DFHBI binding, we solved an X-ray crystal structure (Supplementary Table 3) of one of the positively allosteric Ca^2+^-responsive mFAPs harboring one EF-hand motif, EF1p2_mFAP2b, in complex with DFHBI and Ca^2+^ (Fig. 5a, b and Supplementary Fig. 15). The EF1p2_mFAP2b–DFHBI–Ca^2+^ co-crystal structure revealed the residue K101 from the Ca^2+^-bound EF-hand motif forms a hydrogen bond to the hydroxybenzylidene moiety of DFHBI, providing structural insight into the allosteric coupling mechanism between DFHBI and Ca^2+^ binding (Fig. 5c). Indeed, the K101A lysine-to-alanine substitution in EF1p2_mFAP2b reduces DFHBI affinity ~21-fold in the presence of excess Ca^2+^ ($K_{\mathrm{d}}^ +$), and Ca^2+^ affinity ~29-fold in the presence of excess DFHBI, compared with EF1p2_mFAP2b (Supplementary Fig. 16). This lysine residue is the second amino acid of the first EF-hand motif in all of the Ca^2+^-responsive mFAPs, suggesting it influences the allostery in each case. Molecular dynamics (MD) simulations starting from the X-ray crystal structure coordinates of EF1p2_mFAP2b in four conditions (apo, Ca^2+^-bound, DFHBI-bound and with both Ca^2+^ and DFHBI) suggest Ca^2+^ binding to loop7 shifts the free energy landscape towards the holo (fluorescently active) conformation even in the absence of DFHBI (Supplementary Fig. 17), suggesting a conformational selection^31,32^ mechanism consistent with the experimentally observed allosteric coupling of DFHBI and Ca^2+^ binding (Supplementary Fig. 10).

To explore whether the Ca^2+^-responsive mFAPs could detect Ca^2+^ fluxes in mammalian cells, we first expressed a positively allosteric Ca^2+^-responsive mFAP harboring one EF-hand motif, EF1p_mFAP2b, in the extracellular matrix of HEK293 cells by fusion to an N-terminal immunoglobulin κ-chain leader sequence secretion signal and a C-terminal transmembrane anchoring domain from platelet-derived growth factor receptor (PDGFR). To optimize detection sensitivity and photostability while compromising on fluorescence dynamic range (see Supplementary Note 1), we chose to label HEK293 cells with DFHBI concentrations at approximately the $\left( {K_{\mathrm{d}}^ + \cdot K_{\mathrm{d}}^ - } \right)^{1/2}$ for EF1p_mFAP2b, where $K_{\mathrm{d}}^ +$ and $K_{\mathrm{d}}^ -$ are the DFHBI *K*_d_ for the Ca^2+^-bound or Ca^2+^-free sensor, respectively (Supplementary Table 2). Titration of Ca^2+^ from 0 to 10 mM final concentration under constant DFHBI concentration resulted in a fluorescence fold-change ($\frac{{{\Delta} F}}{{F_0}}$) of ~0.5 (Fig. 5d). The fluorescence response was similar after photobleaching the cells, presumably due to the high-chromophore concentrations improving the photostability of mFAPs (Fig. 2).

Next, we expressed negatively allosteric Ca^2+^-responsive mFAPs containing two or four EF-hand motifs (EF2n_mFAP2a, EF4n_mFAP2b, and EF4n_mFAP2a) in the cytosol of HEK293 cells and stimulated Ca^2+^ release into the cytosol via endogenous muscarinic receptors^19^ by treatment with 100 μM acetylcholine (ACh). As expected, Ca^2+^ release into the cytosol resulted in a decrease in fluorescence upon ACh stimulation (Fig. 5e and Table 2) with DFHBI concentrations at approximately $\left( {K_{\mathrm{d}}^ + \cdot K_{\mathrm{d}}^ - } \right)^{1/2}$, which balances detection sensitivity and photostability against fluorescence dynamic range (Supplementary Note 1). Compared to the positive control fluorescent Ca^2+^ sensor^29^ GCaMP6f (Supplementary Fig. 18), the Ca^2+^-responsive mFAPs have lower fluorescence dynamic range at the DFHBI concentrations used, but are highly photostable (Fig. 5e, first 20 s).

**Table 2:** Fluorescence response to acetylcholine stimulation of HEK293 cells expressing cytosolic Ca^2+^-responsive mFAPs or GCaMP6f.

Sensor	Peak $| {\frac{{{\Delta} {\boldsymbol{F}}}}{{{\boldsymbol{F}}_0}}} |$	[DFHBI] (µM)	Regions of interest	Fluorescence response to increased [Ca^2+^]
EF2n_mFAP2a	0.12 ± 0.092	20.0	15	Negative
EF4n_mFAP2b	0.42 ± 0.14	43.3	10	Negative
EF4n_mFAP2a	0.46 ± 0.12	43.3	15	Negative
GCaMP6f	11 ± 1.8	0.00	12	Positive

The mean and s.d. of the mean of peak absolute values of the fluorescence fold-change (peak $| {\frac{{{\Delta} F}}{{F_0}}} |$) upon acetylcholine (ACh) stimulation over the indicated number of regions of interest surrounding single cells (3 technical replicates per sensor; Fig. 5e and Supplementary Fig. 18).

As the fluorescence of negatively allosteric Ca^2+^-responsive mFAPs increases when the Ca^2+^ concentration decreases, negatively allosteric Ca^2+^-responsive mFAPs enable reporting Ca^2+^ effluxes from compartments as increases in fluorescence signal (similar to inverse-pericam^8,33^ Ca^2+^-responsiveness). We expressed a negatively allosteric Ca^2+^-responsive mFAP containing one EF-hand motif, EF1n_mFAP2b, in the sarcoplasmic reticulum (SR) of cultured human induced pluripotent stem cell (hiPSC)-derived cardiomyocytes (CMs), as existing Ca^2+^-responsive fluorescent protein sensors targeted to the SR report Ca^2+^ effluxes as decreases in fluorescence signal^34,35^. Again optimizing photostability while compromising on fluorescence fold-change, we chose to label CMs with a DFHBI concentration at approximately $\left( {K_{\mathrm{d}}^ + \cdot K_{\mathrm{d}}^ - } \right)^{1/2}$ for EF1n_mFAP2b (Supplementary Table 2). Ca^2+^ transients in the SR during cardiac contraction cycling were detectable with high photostability (Supplementary Fig. 19). Labeling CMs with DFHBI concentrations at approximately $\frac{{K_{\mathrm{d}}^ - }}{2}$ for EF1n_mFAP2b (Supplementary Table 2) resulted in robustly detectable Ca^2+^ transients during cardiac contraction cycling and moderate photostability using ~3-fold higher laser power density (Fig. 5f). The image acquisition rate in these experiments was ~16.7 Hz (60 ms frame^−1^), suggesting that EF1n_mFAP2b responds to Ca^2+^ fluxes in less than 60 ms. Temporal analysis averaged over 20 cardiac contraction cycles revealed a 96 ± 45 ms (mean ± s.d.) lag time between peak SR fluorescence and peak cardiac contraction, indicating that endogenous SR Ca^2+^ signaling precedes cardiomyocyte contractile motion. Inhibition of Ca^2+^ reuptake into the SR by treatment with cyclopiazonic acid (CPA) resulted in a sustained increase in fluorescence in the SR consistent with inhibition of SERCA pumps^34,36^ (Supplementary Fig. 19).

### Split fluorescence-activating proteins

We next sought to design bipartite split fluorogenic sensors^13^ from mFAPs by creating split points in the β-hairpins and loop7 of the mFAP2a scaffold. With eight β-strands^1^ per β-barrel, there are seven possible bipartite split mFAPs (Supplementary Fig. 20). As the split mFAP fragments would have solvent-exposed hydrophobic patches that could hamper solubility, we initially tagged split mFAP fragments to maltose-binding protein (MBP) to improve soluble expression levels. β-barrel complementation assays in excess DFHBI-1T showed that split mFAP fragments m12 and m38 displayed the highest fluorescence activation above background, with 7.34-fold higher mean fluorescence intensity over mean background fluorescence intensity. After background subtraction, the brightest fragment combination, m12 and m38, had 184-fold higher mean fluorescence intensity than the dimmest fragment combination, m1 and m28. Differences in the fluorescence excitation spectra of the fluorescently active β-barrel complexes in excess DFHBI-1T suggest that bipartite split mFAPs stabilize the fluorescently active *cis*-planar conformation of DFHBI-1T in slightly different chromophore environments (Supplementary Fig. 20).

**Fig. 6:**
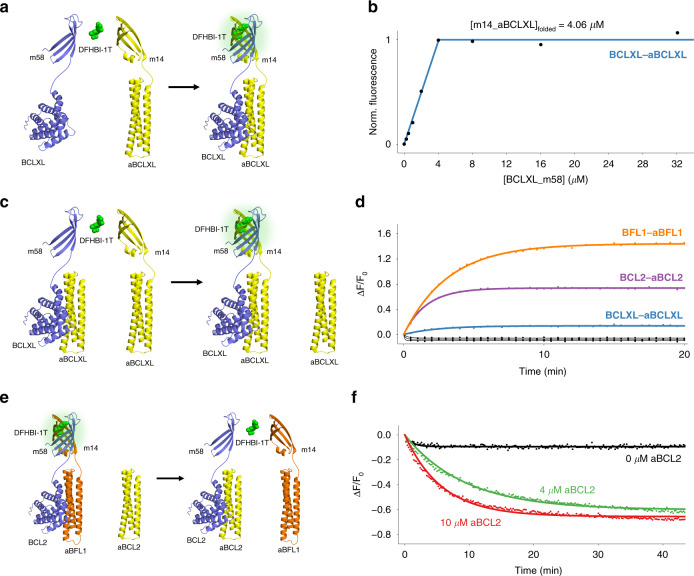
Assembly and disassembly of bipartite split mFAP fragments m14 and m58. **a**–**d** Assembly of split mFAP fragments. **a** Association model in which BCLXL is fused to m58 (BCLXL_m58) *(violet cartoon)*, aBCLXL is fused to m14 (m14_aBCLXL) *(yellow cartoon)*, and fluorescence of DFHBI-1T *(green spheres)* is activated upon association *(arrow)* of BCLXL_m58 and m14_aBCLXL. **b** Normalized fluorescence intensity *(points)* of BCLXL_m58 titration into a constant m14_aBCLXL concentration in excess DFHBI-1T after reaching equilibrium, showing the fit to a bimolecular association model *(line)* using non-linear least squares fitting. **c** Split mFAP competitor pre-incubation model in which fluorescence of DFHBI-1T *(green spheres)* is activated upon competition *(arrow)* of m14_aBCLXL with unfused aBCLXL *(yellow cartoons)* for the BCLXL-binding cleft of BCLXL_m58 *(violet cartoon)* (the reaction evolves analogously for BFL1–aBFL1 and BCL2–aBCL2 cognate-binding partners). **d** Temporal evolution of fluorescence fold-change in excess DFHBI-1T upon (*n* = 1) addition of equimolar m14_aBFL1 *(orange points)* or buffer *(black points)* to pre-incubated equimolar BFL1_m58 and aBFL1, addition of equimolar m14_aBCL2 *(violet points)* or buffer *(black points)* to pre-incubated equimolar BCL2_m58 and aBCL2, and addition of equimolar m14_aBCLXL *(blue points)* or buffer *(black points)* to pre-incubated equimolar BCLXL_m58 and aBCLXL, showing the fits to a monophasic exponential function *(lines)* using non-linear least squares fitting. **e**, **f** Disassembly of split mFAP fragments. **e** Pre-assembled split mFAP competition model in which BCL2 is fused to m58 (BCL2_m58) *(violet cartoon)*, aBFL1 is fused to m14 (m14_aBFL1) *(orange cartoon)*, and fluorescence of DFHBI-1T *(green spheres)* is activated before unfused aBCL2 *(yellow cartoon)* competes with m14_aBFL1 for the BCL2-binding cleft of BCL2_m58 *(arrow)*, resulting in fluorescence deactivation. **f** Temporal evolution of fluorescence fold-change in excess DFHBI-1T of pre-incubated equimolar BCL2_m58 and m14_aBFL1 at 2.00 µM final concentrations with unfused aBCL2 titrated in at (*n* = 1) 0 µM *(black points)*, 4.00 µM *(green points)*, and 10.0 µM *(red points)* final concentrations, showing the fits to a monophasic exponential function *(lines)* using non-linear least squares fitting. Source data is available for Fig. 6b, d, f.

Titrations of MBP-tagged split mFAP fragments into their complementary MBP-tagged split mFAP fragments in excess DFHBI-1T resulted in reconstitution of fluorescence at high-protein concentrations, but the signal did not plateau even at the highest concentrations tested. The estimated split mFAP fragment dissociation constants (*K*_d_ values) are ≥281 µM for m12 and m38, ≥22.0 µM for m14 and m58, ≥232 µM for m16 and m78, and ≥354 µM for m17 and m8 (Supplementary Fig. 20). In contrast, when we fused complementary split mFAP fragments to BCL2 family member proteins and high affinity (*K*_d_ $\simeq$ 1 nM) designed binding partners^37^ (Fig. 6a), the fluorescence increased linearly until reaching a plateau at equimolar concentrations of complementary split mFAP fragments (Fig. 6b).

To assess whether split mFAPs could be used for real-time monitoring of protein–protein association, we pre-incubated equimolar BCLXL_m58 with unfused aBCLXL in excess DFHBI-1T to pre-assemble non-fluorescent BCLXL_m58–aBCLXL complex. Upon addition of equimolar m14_aBCLXL (or buffer as a negative control), the fluorescence increased as m14_aBCLXL competed with unfused aBCLXL for the BCLXL-binding cleft of BCLXL_m58, resulting in assembly of the m14–m58 complex, which activates the fluorescence of DFHBI-1T (Fig. 6c, d). The reaction evolved analogously for BFL1–aBFL1 and BCL2–aBCL2 cognate-binding partners. Different peak fluorescence fold-changes observed amongst split mFAP fusions to BCLXL–aBCLXL, BCL2–aBCL2, and BFL1–aBFL1 complexes suggest that the molecular geometry of the heterodimer interaction affects the brightness of the assembled β-barrel complex. Fluorescence excitation spectra revealed a prominent peak in fluorescence excitation wavelength at 488 nm upon combining split mFAP fragments compared to buffer negative controls (Supplementary Fig. 21).

To assess whether split mFAPs could be used for real-time monitoring of protein–protein dissociation, we pre-incubated BCL2_m58 with equimolar m14_aBFL1 in excess DFHBI-1T to pre-assemble fluorescent complexes. As the non-cognate BCL2–aBFL1 complex has a dissociation constant (*K*_d_) of 320 ± 40 nM, the cognate BCL2–aBCL2 complex has a *K*_d_ of 0.8 ± 0.5 nM^37^, and aBFL1 and aBCL2 interact with the same binding cleft of BCL2, aBCL2 should outcompete aBFL1 for binding to BCL2 (Fig. 6e). Indeed, titration of aBCL2 into pre-assembled BCL2_m58–m14_aBFL1 complex in excess DFHBI-1T resulted in an aBCL2 concentration-dependent decrease in fluorescence (Fig. 6f). Fluorescence excitation spectra showed the disappearance of the fluorescence excitation peak at 488 nm wavelength consistent with chromophore unbinding and deactivation of fluorescence upon split mFAP fragment disassembly (Supplementary Fig. 21).

### Circular permutation

To further explore the range of possibilities for mFAP-based sensors, we circularly permuted mFAPs (cpmFAPs) using Rosetta and the split points from the four brightest bipartite split mFAPs. The brightest cpmFAP tested, cp35-34_mFAP2a_12, has a de novo designed α-helical linker and displayed ~93% of the fluorescence intensity of mFAP2a at equimolar concentration and excess DFHBI-1T (Supplementary Fig. 22). Size-exclusion chromatography with multi-angle light scattering showed cp35-34_mFAP2a_12 to be monomeric (Supplementary Fig. 23).

## Discussion

We have demonstrated that the functionality and brightness of mFAPs can be readily extended by structure-based design and engineering. It should be emphasized, however, that the engineering of useful fluorogenic sensors based on mFAPs is still in its early days–the mFAPs were first described in September 2018^1^. Currently, existing fluorescent protein-based sensors still have numerous advantages over mFAPs in brightness (e.g., EGFP is ~2.1-fold brighter than the mFAP10–DFHBI-1T complex) for applications involving single molecule localization microscopy^15,38^, fluorescence spectral diversity^39^, higher Ca^2+^ affinity^7^, and higher fluorescence dynamic range^29^, as in self-labeling chemigenetic indicators^40–42^. These sophisticated reporters and sensors reflect decades of work by many groups; we hope this report will stimulate exploration of de novo designed fluorescence-activating proteins. With further optimization using both selection and computational design methodologies, there is likely considerable room for improvement of brightness, photostability, pH-responsiveness, and Ca^2+^-responsiveness.

At this stage, the possible advantages of de novo designed mFAP sensors over existing fluorescent protein-based reporters are listed in Table 3. Two notable advantages of split mFAPs over existing split GFP-based approaches for monitoring transient protein–protein interactions are the rapid activation of fluorescence upon assembly of split mFAP fragments that enables tracking of protein–protein association, and the rapid deactivation of fluorescence upon disassembly of split mFAP fragments that enables tracking of protein–protein dissociation (similar to splitFAST but with ~2 orders of magnitude lower fragment affinities). mFAPs can activate the fluorescence of DFHBI-derived chromophores with emission spectra in different color ranges, as illustrated by the activation of DFHO^5^ fluorescence by mFAP3. mFAPs can be used as modular fluorogenic optical sensors for detection and quantification of other small-molecules, ions, or proteins by insertion of their respective binding peptides into the loops of mFAPs without circularly permuting the mFAPs, as in construction of Ca^2+^-responsive mFAPs. The cpmFAPs enable design of modular fluorogenic sensors by fusing analyte binding peptides (e.g., calmodulin and M13) directly to the juxtaposed termini or within β-sheets, as in construction of GCaMP^19,29^. More generally, as brighter and more photostable fluorogenic compounds are developed, the methodologies described herein should be readily applicable to creating protein-based fluorogenic optical sensors from binders to these compounds.

**Table 3:** Potential advantages of de novo designed mFAP sensors over existing fluorescent protein-based reporters and sensors.

Biophysical property	Advantage
Size	Smaller size^1^ than GFP-based^8^, DiBs^15^ and FAST-based^7,16^ reporters (Supplementary Data 1).
Photostability	Higher photostability over AcGFP1 in fixed mammalian cells (Fig. 2).
Reversible fluorescence	Spatiotemporal control over fluorescence via on-demand labeling protocols (Supplementary Fig. 4), unlike intrinsically fluorescent proteins^8,50^, and like DiBs^15^ and FAST-based^7,16^ reporters.
Ratiometric fluorescence dynamic range	Higher ratiometric fluorescence dynamic range across the physiologically relevant pH range than pHRed^20,51^ and pHluorin2^21^ (Fig. 3).
Low-Ca^2+^ affinity	Lower Ca^2+^ affinity than existing fluorescent protein-based Ca^2+^ sensors such as GCaMPer^34^ and CatchER^18^ for imaging Ca^2+^ transients in high-Ca^2+^ concentration environments (Fig. 5f and Supplementary Table 2).
Rapid response of split mFAPs	Rapid change in fluorescence intensity upon split mFAP fragment assembly (Fig. 6d), similar to splitFAST in not requiring chromophore maturation like split GFP^10,11^.
Reversibility of split mFAPs	Reversible fluorescence of split mFAPs, unlike split GFP^10,11^, and similar to splitFAST^13^ but with lower fragment affinities (Fig. 6f and Supplementary Fig. 20).

## Methods

### Design of brighter and pH-responsive mFAPs

A previously described^1^ mFAP2 computational design model was used as a template for manual selection and design of mutable residues using Rosetta^23,43^ macromolecular modeling software. Guided by the previously generated deep mutational scanning maps^1^ of stability and fluorescence of b11L5F, we constructed three mFAP2 mutational variants mFAP2(P50T,S52V), mFAP2(S52T), and mFAP2(P50T,S52V,G100D) that were expected to improve the stability of the protein while also aiding crystallization. Circular dichroism in the absence of DFHBI revealed that mFAP2(P50T,S52V), hereafter called mFAP2.1, demonstrated improved stability at pH 2.93 (Supplementary Fig. 1b, c) and higher fluorescence in the presence of DFHBI at pH 3.66 (Supplementary Fig. 1d, e) compared to mFAP2, consistent with improved binding of the phenolic form of DFHBI to the stabilized protein. A minimal site-directed mutagenesis (SDM) library (Supplementary Data 3) was generated at 15 residue positions on mFAP2.1 encoding mutations hypothesized to improve the fluorescence ratio fold-change from low to high pH, increase DFHBI affinity, and reduce conformational flexibility of the loop connecting the seventh and eighth β-strands (known as loop7) juxtaposing the DFHBI-binding pocket.

Fluorescence screening of the SDM library at pH 3.66 and pH 7.36 revealed that the most pH-responsive mutant mFAP2.1(T50P), also known as mFAP2.2, demonstrated ~1.3-fold higher fluorescence ratio fold-change from pH 3.66–7.36 than mFAP2.1 (Supplementary Fig. 1f). Subsequently, two independent combinatorial libraries were generated from mFAP2.2: one at five positions aimed at increasing loop7 rigidity (Supplementary Data 4), and another at eight positions aimed at optimizing hydrophobic packing of residues in the hydrophobic β-barrel core (Supplementary Data 5). The brightest variant from the first library mFAP2.2(A100E,G101N,N102D,T104H), hereafter known as mFAP2.3, and the brightest variant of the second library mFAP2.2(M27W,V39I,V57A,F93W), hereafter known as mFAP2.4, showed an increase in fluorescence intensity from the phenolate form of DFHBI of ~1.1-fold and ~3.4-fold from mFAP2.2 at pH 7.36, respectively (Supplementary Fig. 1g,h). The mutations producing mFAP2.3 and mFAP2.4 were combined into one scaffold generating mFAP2.5. A single mutation (V67I) was identified by screening a combinatorial library (Supplementary Data 6) of mutations at 7 positions aimed at packing more methyl groups into the hydrophobic β-barrel core of mFAP2.5. The protein (Fig. 1a), referred to as mFAP2b (“b” for bright), is ~1.2-fold brighter than mFAP2.5 and ~1.3-fold brighter than mFAP2.4 at neutral pH (Supplementary Fig. 1i). It was demonstrated that mFAP2b had ~10-fold weaker affinity for DFHBI than the initial mFAP2 design.

Another combinatorial library (Supplementary Data 7) was generated at five positions of mFAP2b aimed at increasing affinity for the deprotonated state of DFHBI without compromising fluorescence intensity by packing both aromatic and aliphatic residues in the hydrophobic β-barrel core of mFAP2b. Screening for fluorescence intensity of the phenolate state of DFHBI using a relatively low-DFHBI concentration (555 nM) resulted in mFAP2b(V13A,M15F), known as mFAP2a (“a” for affinity). mFAP2a displayed ~1.3-fold brighter fluorescence than mFAP2b at low-DFHBI concentration (Supplementary Fig. 1j). Aiming to further improve packing of methyl groups in the hydrophobic β-barrel core to increase the fluorescence intensity of the phenolate state of DFHBI at neutral pH while accommodating the interesting geometry of the Y71W mutation, a combinatorial library (Supplementary Data 8) was constructed at three positions of mFAP2a, resulting in designs mFAP3 through mFAP8 (Supplementary Table 1). However, these mutants were dimmer and demonstrated lower expression levels than mFAP2b or mFAP2a (Supplementary Table 1), although mFAP3 showed to be the brightest DFHO-binding variant (mFAP3 with 10.0 µM DFHO is ~10-fold dimmer than mFAP2b with 10.0 µM DFHBI). In order to increase the fluorescence brightness of mFAP2a with DFHBI-1T, a final combinatorial library was constructed at six positions of mFAP2a (Supplementary Data 9) by mutating aliphatic and aromatic residues in the chromophore-binding pocket of mFAP2a juxtaposing DFHBI-1T in computational models. Screening for fluorescence intensity of DFHBI-1T using a low-DFHBI-1T concentration (250 nM) resulted in mFAP2a(W27I,W93F), also known as mFAP10. An additional 14 designs (including mFAP_pH) were constructed by combining loop7 and hydrophobic β-barrel core mutations from the variants demonstrating the highest fluorescence intensity of the deprotonated state of DFHBI from the mFAP2.2 loop7 combinatorial library (Supplementary Data 4) and mFAP2.2 hydrophobic β-barrel core combinatorial library (Supplementary Data 5 and Supplementary Fig. 1g, h).

### Design of chromophore-selective mFAPs

Computational modeling of DFHBI (Fig. 1b) into the binding pocket of mFAP2b (Fig. 1f) and mFAP2a (Fig. 1h) using Rosetta^23,43^ macromolecular modeling software showed that the mutations V13A and M15F resulted in a void in the binding pocket of mFAP2a. It was hypothesized that a commercially available structural derivative of the DFHBI chromophore with a trifluoromethyl group, DFHBI-1T^4^ (Fig. 1c), could pack into the void without causing steric clashes. Computational modeling of DFHBI-1T in the pocket of mFAP2a (Fig. 1i) demonstrated good protein–chromophore shape complementarity, whereas DFHBI-1T modeled into the mFAP2b pocket (Fig. 1g) resulted in steric clashes. Studying the fluorescence of mFAP2a and mFAP2b in the presence of DFHBI-1T experimentally validated this (Fig. 1d, e). Relative fluorescence intensities and binding affinities of DFHBI, DFHBI-1T, and DFHO for selected mFAP variants were then measured at neutral pH (Supplementary Table 1). The fluorescence intensity of mFAP2a with DFHBI-1T was improved upon screening the “IN2” combinatorial library, resulting in mFAP10.

### Design of extended loop library

β-hairpin loop fragments from the RCSB Protein Data Bank [www.rcsb.org] were used to manually curate custom β-barrel loop fragment databases. RosettaRemodel^44^ was used to fix the β-hairpin loop termini to loops 1, 3, 5, and 7 of the de novo β-barrel scaffolds b11 and b32^1^, picking 3-mer and 9-mer fragments from the custom β-barrel loop fragment databases, from which 2226 designs with successfully closed loops were generated. Loop coordinates were extracted as .pdb files and used as templates to generate Rosetta blueprint files specifying amino acid type, secondary structure, and ABEGO^45^ type of loops to be rebuilt onto loops 1, 3, 5, and 7 of a computational model of mFAP2b, resulting in 8904 Rosetta blueprint files. For each blueprint file, a RosettaScripts^22,23^ XML script (Supplementary Note 2) was used to graft the loop onto mFAP2b with a centroid energy function followed by Monte Carlo^46^ sampling of protein side-chain repacking and protein side-chain and backbone minimization steps in a full-atom Cartesian coordinate energy function^47^. Seven-thousand seven-hundred forty-eight resulting designs were filtered for the following computational protein design metrics (as scored from the XML script described in Supplementary Note 2): geometry = 1; total_score_res ≤ −3.72714; holes ≤ −1.2729; pstat ≥ 0.755044; buns_sc_heavy ≤ 2; buns_bb_heavy ≤ 2; interfE ≤ −38.375; SC ≥ 0.734076; p_aa_pp ≤ −40.8947; and omega ≤ 2.8757. Only 59 designs with extended loops built onto loops 1, 3 and 7 of mFAP2b passed the filter criteria for experimental testing (Supplementary Data 11).

### Design of Ca^2+^-responsive mFAPs

The mFAP2b loop7 sequence and the five extended loop7 sequences shown to confer fluorescence (Supplementary Fig. 6) were sampled as linkers for grafting the sequence of one EF-hand motif from Protein Data Bank (PDB) accession code 1NKF^24^ onto loop7 of mFAP2b. An in-house script (Supplementary Note 3) was written to prune the experimentally validated extended loop7 sequences one residue at a time keeping up to four residues on the N-terminal and C-terminal linkers relative to the grafted EF-hand motif, optionally adding an additional glycine residue on the N-terminal linker and optionally adding an additional glycine or proline residue on the C-terminal linker. This combinatorial library (Supplementary Data 12) had a theoretical diversity of 1140 linkers. The linkers resulting in positively and negatively allosteric Ca^2+^-responsive mFAPs containing one EF-hand motif were combinatorially sampled to act as linkers for grafting two EF-hand motifs from PDB accession code 1FW4^25^ onto loop7 of mFAP2b, where the N-terminal helix of PDB accession code 1FW4 was truncated up to homologous residues on successfully grafted single EF-hand motif designs. This combinatorial library (Supplementary Data 13) had a theoretical diversity of 385 linkers. The linkers resulting in negatively allosteric Ca^2+^-responsive mFAPs containing two EF-hand motifs were combinatorially sampled to act as linkers for grafting four EF-hand motifs from PDB accession code 1PRW^26^ onto loop7 of mFAP2b, where the N-terminal helix of PDB accession code 1PRW was truncated up to homologous residues on successfully grafted single EF-hand motif designs. This combinatorial library (Supplementary Data 14) had a theoretical diversity of 25 linkers.

### Design of split mFAPs and cpmFAPs

Split mFAPs were designed by manually inspecting the single-chain mFAP2a computational design model. In designing split mFAP fusions to BCL2 family heterodimers, linker compositions and lengths were chosen by manually inspecting the split mFAP2a computational design models and available crystal structures (PDB accession codes 5JSN and 5JSB). Split mFAP2a fragments were fused to maltose-binding protein, BCL2, aBCL2, BFL1, aBFL1, BCLXL, and aBCLXL after cysteine residues unlikely to be participating in disulfide bonds were mutated to serine or alanine residues (Supplementary Data 1).

Circularly permuted mFAP2a and mFAP2b were generated from mFAP2a and mFAP2b computational models using Rosetta^23^ and custom scripts in which N- and C-termini (“split points”) were selected at mFAP loop2, loop4, loop6, and loop7 locations, and the two N-terminal and two C-terminal residues of cpmFAP scaffolds were re-designed compared to their respective residue types in mFAP2a (Supplementary Note 4). De novo structured and unstructured linkers covalently fusing the canonical mFAP termini were designed using RosettaScripts^22,23^, and 4000 resulting designs were filtered and sorted on design metrics. The top 12 designs were chosen for experimental testing after 3 circularly permuted mFAP2b variants were mutated to circularly permuted mFAP2a variants using the (V13A, M15F) double point mutation (in canonical mFAP residue numbering) (Supplementary Fig. 22a, b). In the subsequent round of cpmFAP designs, the de novo designed linker sequences from cp35-34_mFAP2a_12, cp35-34_mFAP2a_10, cp35-34_mFAP2a_08, and cp35-34_mFAP2a_11 were each sampled with the four split points described above, and the two N-terminal and two C-terminal residues in the cpmFAP were reverted back to their respective residue types in mFAP2a (Supplementary Fig. 22d and Supplementary Data 1).

### Synthetic DNA construction

For combinatorial libraries, oligonucleotides with degenerate codons encoding desired mFAP sequences were designed using SwiftLib^48^. Overlapping forward and reverse oligonucleotides with degenerate and/or non-degenerate codons spanning the mFAP gene of interest were synthesized (IDT DNA). Oligonucleotides spanning identical gene regions were pooled at equimolar ratios relative to the theoretical amino acid diversity encoded by each gene region. Full-length genes were constructed using assembly polymerase chain reaction (PCR) with Phusion polymerase (NEB). For the extended loop library, the loop1, loop3, and loop7 libraries were assembled separately, and the concentrations of assembly PCR products consisting of full-length genes were quantified on a NanoDrop 8000 (Thermo Scientific) and the three libraries pooled in quantities proportional to the theoretical library diversities of assembly PCR products. Successfully assembled full-length genes, as well as synthetic gBlock (IDT DNA) oligonucleotides encoding full-length protein sequences, with 5′ and 3′ flanking vector backbone sequences were sub-cloned into the pET15b vector (Novagen) or the pcDNA5/FRT/TO vector (ThermoFisher Scientific) using Gibson assembly. The mFAP2.2 loop7 combinatorial library was constructed via Gibson assembly of polyacrylamide gel electrophoresis (PAGE)-purified duplex oligonucleotides into pET15b-mFAP2.2 linear vector DNA. The full-length genes encoding pHRed^20^ and pHluorin2^21^ were synthesized and cloned into the pET29b(+) vector (IDT DNA). Cloned DNA constructs were transformed into Lemo21(DE3) competent *E. coli* (NEB) and plated onto lysogeny broth (LB)-agar plates supplemented with 50.0 μg mL^−1^ carbenicillin or 50.0–100 μg mL^−1^ kanamycin.

### Screening libraries

The number of *E. coli* colonies picked for functional screening was approximately 4-fold the theoretical diversity of each library. *E. coli* colonies were inoculated into 1.00 mL of LB media supplemented with 50.0 μg mL^−1^ carbenicillin in Nunc 2.0 mL DeepWell 96-well plates (Thermo Scientific), and were grown at 37 °C shaking at 1200 rpm overnight. Twenty-five microliters of these cultures were inoculated into 1.00 mL of fresh LB media supplemented with 50.0 μg mL^−1^ carbenicillin, grown at 37 °C and 1200 rpm for 3–4 h, then 0.5 mM isopropyl β-d-thiogalactopyranoside (IPTG) final concentration was added to each well, and protein expression induced for 4 h at 37 °C shaking at 1200 rpm. Cells were pelleted at 2272 × *g* for 2–5 min and pellets were resuspended in 50.0 μL of lysis buffer #1 (25.0 mM Tris, 300 mM NaCl, 20.0 mM imidazole, pH 8.00) supplemented with 1.00 mg mL^−1^ PMSF, a small amount of deoxyribonuclease I (DNase I) from bovine pancreas (Sigma Aldrich), and 2.00 mg mL^−1^ lysozyme from chicken egg white (Sigma) for lysis. Crude lysates were vigorously shaken at 25–37 °C for 12–48 h, then clarified by centrifugation. Clarified lysates were assayed on a Synergy Neo2 hybrid multi-mode reader (BioTek) in 96-well non-binding surface microplates (Corning 3650) with Gen5 (version 3.03.14) software.

For each clone encoding a β-barrel core variant, loop7 variant, or extended loop variant, 15.0 μL of clarified lysate was combined with 185 μL of Na_2_HPO_4_-citrate (pH 7.36 or pH 3.66) buffer supplemented with 150 mM NaCl and either 1.08 μM DFHBI (Lucerna), 555 nM DFHBI (Lucerna), or 250 nM DFHBI-1T (Lucerna). Na_2_HPO_4_-citrate buffer was made from 200 mM Na_2_HPO_4_ and 100 mM citrate stock solutions, and final pH was adjusted using hydrochloric acid (HCl) or sodium hydroxide (NaOH). DFHBI and DFHBI-1T stock solutions were 2.00 mM in 23.8 mM Tris (pH 8.00), 95.0 mM NaCl, and 5% dimethyl sulfoxide (DMSO). Clones that demonstrated fluorescence were Sanger sequenced via colony PCR of overnight cultures, and the brightest designs or designs with highest fluorescence fold-change across pH 7.36–3.66 were further characterized with large-scale protein purification^49^.

For each clone encoding one or more EF-hand motifs grafted onto β-barrel loop7, 15.0 μL of lysate was combined with 185 μL of either 2.00 mM CaCl_2_ (Sigma Aldrich), 25.0 mM Tris (pH 8.00), 100 mM NaCl, 1.08 μM DFHBI or 2.00 mM EGTA (Sigma Aldrich), 25.0 mM Tris (pH 8.00), 100 mM NaCl, 1.08 μM DFHBI. Clones that demonstrated greater than an approximately twofold change in fluorescence intensity between CaCl_2_ and EGTA conditions were Sanger sequenced, and the designs demonstrating the highest fold-change in fluorescence intensity between CaCl_2_ and EGTA conditions were further characterized with large-scale protein purification^49^.

### Fluorescence intensity assays

To measure fluorescence intensities of the phenolate form of chromophores, fluorescence was measured on a Synergy Neo2 hybrid multi-mode reader (BioTek) in flat bottom, black polystyrene, non-binding surface 96-well microplates (Corning 3650). Fluorescence intensity was measured in triplicate by exciting at *λ*_ex_ = 484 nm and measuring fluorescence emission at *λ*_em_ = 505 nm (or *λ*_em_ = 511 nm for clones harboring the W27 mutation) (Supplementary Fig. 1g, h). The W27 mutation redshifts the peak emission wavelength from *λ*_em_ = ~505 nm to *λ*_em_ = ~511 nm, presumably due to the W27 indole ring donating a hydrogen bond to the imidazolinone moiety of the deprotonated state of DFHBI, which stabilizes the chromophore conjugated π–electron system in the excited state causing the redshift in emission^50^. Fifteen microliters of small-scale purified protein^49^ was combined with 185 μL of Na_2_HPO_4_-citrate (pH 7.36) buffer supplemented with 150 mM NaCl and 108 nM DFHBI for a final concentration of 100 nM DFHBI. In measuring the excitation spectra from *λ*_em_ = 525 nm of each clone in triplicate (Supplementary Fig. 1i), 30.0 μL of large-scale purified protein^49^ was combined with 170 μL of 25.0 mM Tris (pH 8.00) supplemented with 100 mM NaCl and 914 nM DFHBI for a final concentration of 7.77 μM protein and 777 nM DFHBI. In measuring the fluorescence intensity at *λ*_ex_ = 484 nm and *λ*_em_ = 509 nm of each clone in triplicate (Supplementary Fig. 1j), 30.0 μL of large-scale purified protein^49^ was combined with 170 μL of 25.0 mM Tris (pH 8.00) supplemented with 100 mM NaCl and 653 nM DFHBI for a final concentration of 5.55 μM protein and 555 nM DFHBI. In measuring fluorescence intensity at *λ*_ex_ = 468 nm and *λ*_em_ = 530 nm of each clone in technical triplicate (Supplementary Fig. 1k), 24.0 μL of 35.4 µM large-scale purified protein^49^ was combined with 1.00 μL of 1.25 µM DFHBI or 1.00 μL of 1.25 µM DFHBI-1T (Lucerna) (from 2 mM chromophore stock solutions dissolved in 0.5% DMSO and 99.5% high-salt Tev cleavage buffer [25.0 mM Tris, 100 mM NaCl, pH 8.00]) for final concentrations of 34.0 μM protein and 50.0 nM chromophore. The mFAP9–DFHBI complex had ~1.1-fold the fluorescence intensity of the mFAP10–DFHBI complex, and the mFAP9–DFHBI-1T complex had ~0.75-fold the fluorescence intensity of the mFAP10–DFHBI-1T complex (Supplementary Fig. 1k). In collecting fluorescence emission spectra by exciting fluorescence at *λ*_ex_ = 484 nm and collecting fluorescence emission at *λ*_em_ = 495–650 nm (Supplementary Fig. 6b), and in measuring fluorescence endpoints in techincal triplicate at unique peak excitation and emission wavelengths (Supplementary Fig. 6c), 195 μL of large-scale purified protein^49^ was combined with 5.00 μL of 25 mM Tris (pH 8.00) supplemented with 100 mM NaCl and 40.0 μM DFHBI for a final concentration of 10.0 μM protein and 1.00 μM DFHBI.

In measuring fluorescence intensity of small-scale purified proteins^49^ (Supplementary Table 1), wells were excited at *λ*_ex_ = 488 nm for DFHBI and DFHBI-1T and *λ*_ex_ = 505 nm for DFHO, and fluorescence emission measured at *λ*_em_ = 510 nm for DFHBI and DFHBI-1T and *λ*_em_ = 545 nm for DFHO. Fluorescence endpoints were measured using either 25.0 μL or 50.0 μL of small-scale purified protein in either 175 μL or 150 μL, respectively, of Na_2_HPO_4_-citrate (pH 7.36) buffer supplemented with 150 mM NaCl and either 100 nM or 10.0 μM of either DFHBI (Lucerna), DFHBI-1T (Lucerna) or DFHO (Lucerna) (from 20.0 mM chromophore stock solutions in 100% DMSO), for 200 μL final volumes per well.

To measure the fluorescence intensities of circularly permuted mFAP2a variants (Supplementary Fig. 22b, d), fluorescence endpoints were measured on a Synergy Neo2 hybrid multi-mode reader (BioTek) in flat bottom, black polystyrene, non-binding surface 96-well microplates (Corning 3650) or half-area microplates (Corning 3686). Fluorescence endpoints were measured in technical triplicate by exciting at *λ*_ex_ = 488 nm and measuring fluorescence emission at *λ*_em_ = 510 nm (Supplementary Fig. 22b), or exciting at *λ*_ex_ = 468 nm and measuring fluorescence emission at *λ*_em_ = 530 nm (Supplementary Fig. 22d). Ninety microliters of 55.6 µM large-scale purified protein^49^ in high-salt Tev cleavage buffer was combined with 10.0 µL of 5.00 µM DFHBI-1T in high-salt Tev cleavage buffer for final concentrations of 50.0 µM protein and 500 nM DFHBI-1T in 100 µL final volumes (Supplementary Fig. 22b), or 48.0 µL of 41.7 µM large-scale purified protein^49^ in high-salt Tev cleavage buffer was combined with 2.00 µL of 1.25 µM DFHBI-1T in high-salt Tev cleavage buffer for final concentrations of 40.0 µM protein and 50.0 nM DFHBI-1T in 50.0 µL final volumes (Supplementary Fig. 22d).

### Densitometry

In quantifying relative protein expression levels in *E. coli* (Supplementary Table 1), 5.00 μL of small-scale purified proteins^49^ were combined with 5.00 μL of 2x Laemmli buffer, denatured at 99 °C for 10 min, and 5.00 μL of each sample run on Any kD Mini-PROTEAN TGX Stain-Free Precast Gels (Bio-Rad) in Tris-glycine buffer alongside 5.00 μL of Precision Plus Protein Unstained Protein Standard (Bio-Rad). Gels were stained using an eStain L1 Protein Staining System (GenScript) and imaged using a Molecular Imager ChemiDoc XRS+ (Bio-Rad). Relative densitometry was analyzed in Image Lab Software Version 6.0.1 build 34 Standard Edition (Bio-Rad) referenced to the 15 kDa protein ladder band.

### pH-dependent fluorescence assays

pH-dependent fluorescence was measured on a Synergy Neo2 hybrid multi-mode reader in flat bottom, black polystyrene, non-binding surface 96-well microplates (Corning 3650). In measuring the pH-sensitivity of β-barrel variants in technical triplicate (Supplementary Fig. 5a, b), 20.0 μL of large-scale purified protein^49^ was combined with 180 μL of Na_2_HPO_4_-citrate (pH 7.34 or 3.61) supplemented with 150 mM NaCl and 278 nM DFHBI for final concentrations of 2.50 μM protein and 250 nM DFHBI. Buffer wells for background subtraction were prepared identically except using 20.0 μL of high-salt Tev cleavage buffer instead of purified protein. Wells were excited at *λ*_ex_ = 387 nm or *λ*_ex_ = 484 nm and fluorescence emission measured at *λ*_em_ = 501 nm or *λ*_em_ = 505 nm, respectively. Following background subtraction from the mean endpoint fluorescence of buffer controls, the fluorescence ratio fold-change from pH 3.61–7.34 was calculated as:

1 $${\mathrm{Fluorescence}}\,{\mathrm{Ratio}}\,{\mathrm{Fold}} {\hbox{-}} {\mathrm{Change}}\left( {{\mathrm{pH}}\,3.61 {\hbox{-}} 7.34} \right) = \frac{{\left( {\frac{{F_{{\mathrm{pH}}\,3.61}^{\lambda _{{\mathrm{ex}}} = 387\,{\mathrm{nm}},\,\lambda _{{\mathrm{em}}} = 501\,{\mathrm{nm}}}}}{{F_{{\mathrm{pH}}\,3.61}^{\lambda _{{\mathrm{ex}}} = 484\,{\mathrm{nm}},\,\lambda _{{\mathrm{em}}} = 505\,{\mathrm{nm}}}}}} \right)}}{{\left( {\frac{{F_{{\mathrm{pH}}\,7.34}^{\lambda _{{\mathrm{ex}}} = 387\,{\mathrm{nm}},\,\lambda _{{\mathrm{em}}} = 501\,{\mathrm{nm}}}}}{{F_{{\mathrm{pH}}\,7.34}^{\lambda _{{\mathrm{ex}}} = 484\,{\mathrm{nm}},\,\lambda _{{\mathrm{em}}} = 505\,{\mathrm{nm}}}}}} \right)}},$$

where $F_{{\mathrm{pH}}}^{\lambda _{{\mathrm{ex}}},\,\lambda _{{\mathrm{em}}}}$ is the endpoint fluorescence measurement at the subscripted pH value and superscripted fluorescence excitation (*λ*_ex_) and fluorescence emission (*λ*_em_) wavelengths.

In measuring pH-sensitivity of β-barrel variants (Supplementary Fig. 1f) in technical triplicate, 140 μL of Na_2_HPO_4_-citrate (pH 7.36 or 3.66) supplemented with 150 mM NaCl and 1.43 μM DFHBI was combined with 10.0 μL of small-scale purified protein^49^ for a final concentration of 1.33 μM DFHBI. Fluorescence endpoints were measured by exciting at *λ*_ex_ = 387 nm or *λ*_ex_ = 483 nm and measuring fluorescence emission at *λ*_em_ = 501 nm or *λ*_em_ = 504 nm, respectively. Without background subtraction, the fluorescence ratio fold-change from pH 3.66–7.36 was calculated as:

2 $${\mathrm{Fluorescence}}\,{\mathrm{Ratio}}\,{\mathrm{Fold}} {\hbox{-}} {\mathrm{Change}}\left( {{\mathrm{pH}}\,3.66 {\hbox{-}} 7.36} \right) = \frac{{\left( {\frac{{F_{{\mathrm{pH}}\,3.66}^{\lambda _{{\mathrm{ex}}} = 387\,{\mathrm{nm}},\,\lambda _{{\mathrm{em}}} = 501\,{\mathrm{nm}}}}}{{F_{{\mathrm{pH}}\,3.66}^{\lambda _{{\mathrm{ex}}}= 483\,{\mathrm{nm}},\,\lambda _{{\mathrm{em}}} = 504\,{\mathrm{nm}}}}}} \right)}}{{\left( {\frac{{F_{{\mathrm{pH}}\,7.36}^{\lambda _{{\mathrm{ex}}} = 387\,{\mathrm{nm}},\,\lambda _{{\mathrm{em}}} = 501\,{\mathrm{nm}}}}}{{F_{{\mathrm{pH}}\,7.36}^{\lambda _{{\mathrm{ex}}} = 483\,{\mathrm{nm}},\,\lambda _{{\mathrm{em}}} = 504\,{\mathrm{nm}}}}}} \right)}},$$

where $F_{{\mathrm{pH}}}^{\lambda _{{\mathrm{ex}}},\,\lambda _{{\mathrm{em}}}}$ is the mean of three technical replicates of endpoint fluorescence measurements at the subscripted pH value and superscripted fluorescence excitation (*λ*_ex_) and fluorescence emission (*λ*_em_) wavelengths.

In measuring pH-dependent fluorescence (Fig. 3d–i), Na_2_HPO_4_-citrate buffer supplemented with 150 mM NaCl at unique pH values were produced via mixing various volumes of 100 mM citric acid (Sigma Aldrich), 200 mM Na_2_HPO_4_ (Sigma Aldrich), and 2.00 M NaCl, and final pHs quantified via an Accumet AB15 Basic pH meter (Fisher Scientific). pHRed and pHluorin2 were produced via large-scale protein purification, 6xHis-tag removal and size-exclusion chromatography (SEC) purification, and mFAP_pH and mFAP2b were produced via large-scale protein purification and SEC purification^49^. pH-dependent fluorescence was measured at 500 nM final protein concentration for mFAP2b, mFAP_pH and pHRed, and 170 nM final concentration for pHluorin2, at each pH in technical triplicate in 200 μL final volumes per well. To prevent pH fluctuations upon addition of protein and DFHBI, 195 μL of Na_2_HPO_4_-citrate buffers supplemented with 150 mM NaCl at each pH was aliquoted per well, 4.00 μL of purified protein was aliquoted per well, and 1.00 μL of 1.00 mM DFHBI (in 2.5% DMSO and 97.5% high-salt Tev cleavage buffer) was added to wells containing mFAP_pH, whereas 1.00 μL of chromophore buffer (2.5% DMSO and 97.5% high-salt Tev cleavage buffer) was added to wells containing pHRed or pHluorin2. Blank wells for background subtraction for mFAP2b, mFAP_pH, pHRed and pHluorin2 were prepared identically, respectively, except adding 4.00 μL of high-salt Tev cleavage buffer instead of purified protein. In measuring fluorescence excitation spectra at each pH (Fig. 3d), excitation wavelengths were set to the range *λ*_ex_ = 300–530 nm and fluorescence emission measured at *λ*_em_ = 562 nm. In measuring pH-dependent fluorescence excitation spectra at pH 3.76 and pH 7.34 (Fig. 3f, g), excitation spectra were measured using excitation wavelengths in the range *λ*_ex_ = 300–540 nm and emission wavelength *λ*_em_ = 592 nm.

In measuring fluorescence emission spectra at two pH values (Fig. 3e), emission spectra for the blueshifted excitation peak was measured at pH 3.63 using excitation wavelength *λ*_ex_ = 379 nm and emission wavelengths in the range *λ*_em_ = 460–700 nm, and emission spectra for the redshifted excitation peak was measured at pH 8.38 using excitation wavelength *λ*_ex_ = 430 nm and emission wavelengths in the range *λ*_em_ = 460–700 nm. In measuring fluorescence from both the blueshifted and redshifted excitation peaks (Fig. 3h, i), for mFAP_pH the fluorescence excitation wavelengths were *λ*_ex_ = 379 nm and *λ*_ex_ = 483 nm and fluorescence emission wavelengths were *λ*_em_ = 498 nm and *λ*_em_ = 503 nm, respectively, whereas for pHRed the fluorescence excitation wavelengths were *λ*_ex_ = 440 nm and *λ*_ex_ = 575 nm and fluorescence emission wavelengths were both *λ*_em_ = 635 nm, and for pHluorin2 the fluorescence excitation wavelengths were *λ*_ex_ = 405 nm and *λ*_ex_ = 485 nm and fluorescence emission wavelengths were both *λ*_em_ = 535 nm. In Fig. 3i, for the mFAP_pH–DFHBI complex, pHRed, and pHluorin2 the ratiometric fluorescence (*F*_ratio_) is calculated from the background-subtracted, unnormalized fluorescence measurements using fluorescence emission from the redshifted excitation peak ($F^{{\mathrm{redshifted}}\,\lambda _{{\mathrm{ex}}}}$) as the numerator and fluorescence emission from the blueshifted excitation peak ($F^{{\mathrm{blueshifted}}\,\lambda _{{\mathrm{ex}}}}$) as the denominator:

3 $$F_{{\mathrm{ratio}}} = \frac{{F^{{\mathrm{redshifted}}\,\lambda _{{\mathrm{ex}}}}}}{{F^{{\mathrm{blueshifted}}\,\lambda _{{\mathrm{ex}}}}}}.$$

In Fig. 3i, mean ratiometric fluorescence values are fit to continuous logistic functions with the formula $F_{{\mathrm{ratio}}} = \frac{{23.5}}{{1 + {\mathrm{e}}^{ - 2.02\cdot ({\mathrm{pH}} - 6.90)}}}$ for the mFAP_pH–DFHBI complex, $F_{{\mathrm{ratio}}} = \frac{{4.20}}{{1 + {\mathrm{e}}^{2.00\cdot ({\mathrm{pH}} - 6.60)}}}$ for pHRed, and $F_{{\mathrm{ratio}}} = \frac{{5.96}}{{1 + {\mathrm{e}}^{0.522\cdot ({\mathrm{pH}} - 2.80)}}}$ for pHluorin2 using non-linear least squares fitting, which serve as continuous calibration curves for real-time quantification of pH^51^.

In measuring the protonated (phenolic) and deprotonated (phenolate) DFHBI affinities with mFAP_pH (Supplementary Fig. 5c), fluorescence endpoints were measured on a Synergy Neo2 hybrid multi-mode reader (BioTek) in flat bottom, black polystyrene, non-binding surface 96-well microplates (Corning 3650). mFAP_pH was produced by large-scale protein purification^49^ and aliquoted to 200 μL final volumes at 500 nM final concentration in seven serial dilutions ($\sqrt {10}$ dilution factor) of DFHBI starting from 10.0 μM DFHBI final concentration, including an eighth condition without chromophore, in Na_2_HPO_4_-citrate buffer supplemented with 143 mM NaCl final concentration at either pH 3.61 or pH 7.34. For pH 3.61 fluorescence was excited at *λ*_ex_ = 379 nm and fluorescence emission measured at *λ*_em_ = 498 nm, and for pH 7.34 fluorescence was excited at *λ*_ex_ = 483 nm and fluorescence emission measured at *λ*_em_ = 503 nm. At each pH, background fluorescence endpoints of wells with identical chromophore concentrations but purified protein replaced with an identical volume of buffer were measured, and fluorescence endpoints subtracted from those measured with protein. Background-subtracted data were normalized from 0 to 1 and fit to a single-binding site isotherm function using non-linear least squares fitting to obtain the reported *K*_d_ values, which were less than the protein concentrations tested.

### Chromophore titrations

Fluorescence endpoints were measured on a Synergy Neo2 hybrid multi-mode reader (BioTek) in flat bottom, black polystyrene, non-binding surface 96-well microplates (Corning 3650). In measuring chromophore-binding affinities (Fig. 1d, e), mFAP2, mFAP2a, and mFAP2b, and mFAP10 were produced by large-scale protein purification and SEC purification^49^. Proteins were aliquoted in eight technical replicates in 200 μL final volumes to 20.0 nM final concentration in ten serial dilutions ($\sqrt {10}$ dilution factor) of DFHBI starting from 31.6 μM DFHBI or 31.6 μM DFHBI-1T final concentrations, including an eleventh condition without chromophore. Fluorescence was excited at *λ*_ex_ = 468 nm and fluorescence emission measured at *λ*_em_ = 530 nm. Background fluorescence endpoints of wells with identical chromophore concentrations but purified protein replaced with an identical volume of high-salt Tev cleavage buffer were measured, and fluorescence endpoints subtracted from those measured with protein. Background-subtracted data were averaged and the means normalized from 0 to 1 and fit to a single-binding site isotherm function using non-linear least squares fitting to obtain a fitted *K*_d_ value (Table 1), and the fit scaled to the maximum mean value (Fig. 1d, e).

Chromophore affinities (Supplementary Table 1) were obtained by producing proteins via large-scale protein purification (some variants further purified by SEC)^49^ and preparing proteins at 25.0 μL final volumes in flat bottom, black polystyrene, non-binding surface 96-well half-area microplates (Corning 3686). Proteins were aliquoted to 500 nM final concentrations in high-salt Tev cleavage buffer (mFAP2.2.5 was prepared at 386 nM, and mFAP2a and mFAP3 were prepared at 50.0 nM) in eleven serial dilutions ($\sqrt {10}$ dilution factor) from 1.00 mM DFHBI, 1.00 mM DFHBI-1T, or 1.00 mM DFHO, including a twelfth condition without chromophore. Fluorescence endpoints were measured on a Synergy Neo2 hybrid multi-mode reader (BioTek) with excitation and emission wavelengths set differently between protein variants to achieve maximal fluorescence intensity. Background fluorescence endpoints of wells with identical chromophore concentrations but purified protein replaced with an identical volume of high-salt Tev cleavage buffer were measured, and fluorescence endpoints measured with identical instrument settings subtracted from those measured with protein prior to normalization.

### Ca^2+^-responsive mFAP DFHBI titrations

In measuring Ca^2+^-dependent DFHBI affinity of Ca^2+^-responsive mFAPs on a Synergy Neo2 hybrid multi-mode reader (Fig. 4a–c and Supplementary Figs. 10a, b, d, e, g, h,  11a, b, d, e, g, h,  12a, b, d, e, g, h,  16a), large-scale purified proteins^49^ were aliquoted to a final concentration of 500 nM in either 450 mM CaCl_2_ (Sigma Aldrich) (prepared in high-salt Tev cleavage buffer) or high-salt Tev cleavage buffer, along with ten serial dilutions ($\sqrt {10}$ dilution factor) of DFHBI starting from 316 μM DFHBI including an eleventh condition without chromophore. Final volumes were 25.0 μL in flat bottom, black polystyrene, non-binding surface 96-well half-area microplates (Corning 3686) or 200 μL in flat bottom, black polystyrene, non-binding surface 96-well microplates (Corning 3650). Fluorescence endpoints were measured using excitation wavelength *λ*_ex_ = 488 nm and emission wavelength *λ*_em_ = 510 nm. Background fluorescence endpoints of wells with identical chromophore concentrations lacking protein (substituted for equivalent volumes of high-salt Tev cleavage buffer) were measured and subtracted from protein measurements prior to normalization.

For DFHBI titrations of EF4n_mFAP2a and EF4n_mFAP2b (Supplementary Fig. 13a, b), a small amount of Chelex 100 sodium form (Sigma Aldrich) was added to purified protein (produced by large-scale protein purification and SEC purification^49^) and nutated at 4 °C overnight. High-salt Tev cleavage buffer with a small amount of Chelex 100 was prepared and mixed at room temperature overnight, and a 2.00 mM DFHBI stock solution (in 5% DMSO and 95% high-salt Tev cleavage buffer) with a small amount of Chelex 100 was prepared and nutated overnight at 4 °C. Proteins were aliquoted to a final concentration of 5.00 μM in either 500 μM CaCl_2_ (Sigma Aldrich) (prepared in high-salt Tev cleavage buffer) or 500 μM EGTA (Sigma Aldrich) (prepared in high-salt Tev cleavage buffer), along with ten serial dilutions ($\sqrt {10}$ dilution factor) of DFHBI starting from 316 μM DFHBI (using Chelex 100 pre-treated DFHBI stock solution and high-salt Tev cleavage buffer), including an eleventh condition without chromophore. Final volumes were 25.0 μL in flat bottom, black polystyrene, non-binding surface 96-well half-area microplates (Corning 3686). Fluorescence endpoints were measured using excitation wavelength *λ*_ex_ = 488 nm and emission wavelength *λ*_em_ = 510 nm for EF4n_mFAP2b, and excitation wavelength *λ*_ex_ = 478 nm and emission wavelength *λ*_em_ = 520 nm for EF4n_mFAP2a. Background fluorescence endpoints of wells with identical chromophore concentrations lacking protein (substituted for equivalent volumes of Chelex 100 pre-treated high-salt Tev cleavage buffer) were measured and subtracted from protein measurements prior to normalization.

### Ca^2+^-responsive mFAP Ca^2+^ titrations

In measuring Ca^2+^ affinity of mFAPs on a Synergy Neo2 hybrid multi-mode reader (Fig. 4d, e, f and Supplementary Figs. 10c, f, i, 11c, f, i, 13c, f, i, 16b), large-scale purified proteins^49^ were aliquoted in technical triplicate to a final concentration of 500 nM with 5.00 μM DFHBI in eleven serial dilutions ($\sqrt {10}$ dilution factor) of CaCl_2_ (Sigma Aldrich) starting from either 900 mM or 90.0 mM CaCl_2_ (diluted using high-salt Tev cleavage buffer) including a twelfth condition without CaCl_2_. Final volumes were 200 μL in flat bottom, black polystyrene, non-binding surface 96-well microplates (Corning 3650). Fluorescence endpoints were measured using excitation wavelength *λ*_ex_ = 484 nm and emission wavelength *λ*_em_ = 508 nm. Background fluorescence endpoints of wells with identical chromophore and CaCl_2_ concentrations lacking protein (substituted for equivalent volumes of high-salt Tev cleavage buffer) were measured in technical triplicate, averaged per condition, and subtracted from protein measurement averages of the same conditions.

For Ca^2+^ titrations of EF4n_mFAP2a and EF4n_mFAP2b (Supplementary Fig. 13c), a small amount of Chelex 100 sodium form (Sigma Aldrich) was added to purified protein (produced by large-scale protein purification and SEC purification^49^) and nutated at 4 °C overnight. EF4n_mFAP2a was aliquoted in technical triplicate to a final concentration of 8.00 μM with 80.0 μM DFHBI and EF4n_mFAP2b was aliquoted in technical triplicate to a final concentration of 6.25 μM with 43.4 μM DFHBI, in eleven serial dilutions ($\sqrt {10}$ dilution factor) of CaCl_2_ starting from 9.00 mM or 4.00 mM CaCl_2_ (diluted using high-salt Tev cleavage buffer pre-treated with Chelex 100) including a twelfth condition without CaCl_2_. Final volumes were 25.0 μL in flat bottom, black polystyrene, non-binding surface 96-well half-area microplates (Corning 3686) or 200 μL in flat bottom, black polystyrene, non-binding surface 96-well microplates (Corning 3650). Fluorescence endpoints were measured for EF4n_mFAP2a using excitation wavelength *λ*_ex_ = 478 nm and emission wavelength *λ*_em_ = 520 nm, and for EF4n_mFAP2b using excitation wavelength *λ*_ex_ = 484 nm and emission wavelength *λ*_em_ = 508 nm. Background fluorescence endpoints of wells with identical chromophore and CaCl_2_ concentrations lacking protein (substituted for equivalent volumes of high-salt Tev cleavage buffer) were measured in technical triplicate, averaged per condition, and subtracted from protein measurement averages of the same conditions. For non-linear least squares fitting to obtain *K*_d_ values, means were fit to a single-binding site isotherm function where Ca^2+^-binding sites were modeled independently with a Hill coefficient of 1.

### Ca^2+^-responsive mFAP fluorescence spectra

Fluorescence spectra of Ca^2+^-responsive mFAPs were measured on a Synergy Neo2 hybrid multi-mode reader in flat bottom, black polystyrene, non-binding surface 96-well microplates (Corning 3650). In Supplementary Fig. 9a–n, fluorescence excitation spectra were measured using excitation wavelengths *λ*_ex_ = 350–530 nm and emission wavelength *λ*_em_ = 570 nm, and fluorescence emission spectra measured using excitation wavelength *λ*_ex_ = 430 nm and emission wavelengths *λ*_em_ = 470–750 nm. In Supplementary Fig. 9o–t, for optimized signal the fluorescence excitation spectra were measured using excitation wavelengths *λ*_ex_ = 350–510 nm and emission wavelength *λ*_em_ = 550 nm, and fluorescence emission spectra measured using excitation wavelength *λ*_ex_ = 450 nm and emission wavelengths *λ*_em_ = 490–700 nm. 6xHis-tagged proteins were produced via large-scale protein purification^49^ and aliquoted to final volumes of 200 μL per well with final concentrations of either 1.50 mM EGTA (Sigma Aldrich) (prepared in high-salt Tev cleavage buffer), or 750 mM CaCl_2_ (Sigma Aldrich) (prepared in high-salt Tev cleavage buffer), with either: 89.1 μM EF1p_mFAP2b and 1.40 μM DFHBI (Supplementary Fig. 9a); 8.50 μM EF1p_mFAP2a and 600 nM DFHBI (Supplementary Fig. 9b); 51.4 μM EF1p2_mFAP2b and 5.40 μM DFHBI (Supplementary Fig. 9c); 900 nM EF1p2_mFAP2a and 700 nM DFHBI (Supplementary Fig. 9d); 47.4 μM EF1p3_mFAP2b and 1.70 μM DFHBI (Supplementary Fig. 9e); 30.6 μM EF1p3_mFAP2a and 800 nM DFHBI (Supplementary Fig. 9f); 107 μM EF1n_mFAP2b and 2.50 μM DFHBI (Supplementary Fig. 9g); 32.7 μM EF1n_mFAP2a and 1.30 μM DFHBI (Supplementary Fig. 9h); 47.7 μM EF1n2_mFAP2b and 13.8 μM DFHBI (Supplementary Fig. 9i); 30.6 μM EF1n2_mFAP2a and 2.40 μM DFHBI (Supplementary Fig. 9j); 125 μM EF1n3_mFAP2b and 1.60 μM DFHBI (Supplementary Fig. 9k); 13.5 μM EF1n3_mFAP2a and 700 nM DFHBI (Supplementary Fig. 9l); 83.5 μM EF2n_mFAP2b and 5.00 μM DFHBI (Supplementary Fig. 9m); 6.90 μM EF2n_mFAP2a and 1.20 μM DFHBI (Supplementary Fig. 9n); 7.10 μM EF2n2_mFAP2b and 7.30 μM DFHBI (Supplementary Fig. 9o); 1.60 μM EF2n2_mFAP2a and 1.30 μM DFHBI (Supplementary Fig. 9p); 21.1 μM EF2n3_mFAP2b and 13.7 μM DFHBI (Supplementary Fig. 9q); 1.50 μM EF2n3_mFAP2a and 4.60 μM DFHBI (Supplementary Fig. 9r); 14.7 μM EF4n_mFAP2b and 14.5 μM DFHBI (Supplementary Fig. 9s); or 5.60 μM EF4n_mFAP2a and 31.4 μM DFHBI (Supplementary Fig. 9t).

### EF2n_mFAP2b DFHBI titration versus Ca^2+^ titration heatmap

EF2n_mFAP2b was produced by large-scale protein purification^49^ and aliquoted to a final concentration of 500 nM in eleven serial dilutions ($\sqrt {10}$ dilution factor) of CaCl_2_ starting from 4.50 mM CaCl_2_ along columns, and eight serial dilutions ($\sqrt {10}$ dilution factor) of DFHBI starting from 500 µM DFHBI along rows, at final volumes of 25.0 μL in flat bottom, black polystyrene, non-binding surface 96-well half-area microplates (Corning 3686). Fluorescence endpoints were measured on a Synergy Neo2 hybrid multi-mode reader using excitation wavelength *λ*_ex_ = 484 nm and emission wavelength *λ*_em_ = 508 nm. Raw data (without background subtraction) were normalized from 0 to 1 and reported (Supplementary Fig. 14c, *top row*).

### Split mFAP titration assays

To measure fluorescence intensities in a protein-fragment complementation assay^12,13,52^ (Supplementary Fig. 20b), fluorescence was measured on a Synergy Neo2 hybrid multi-mode reader (BioTek) in flat bottom, black polystyrene, non-binding surface 96-well half-area microplates (Corning 3686). In technical triplicate, 12.0 µL of each split mFAP fragment covalently fused to maltose-binding protein (MBP) was mixed to an equimolar concentration supplemented with 1.00 µL of 1.25 mM DFHBI-1T (Lucerna) at 25.0 μL final volumes per well. Fluorescence endpoints were measured using excitation wavelength *λ*_ex_ = 478 nm and emission wavelength *λ*_em_ = 520 nm. In technical triplicate, background fluorescence endpoints of wells with identical chromophore concentrations lacking protein (substituted for equivalent volumes of high-salt Tev cleavage buffer) were measured, and the mean fluorescence endpoints were subtracted from the mean fluorescence endpoints of samples containing protein.

Split mFAP fragment affinities (Supplementary Fig. 20d–g) were estimated by preparing MBP-tagged split mFAP fragments by large-scale protein purification^49^ in high-salt Tev cleavage buffer, with 25.0 µM DFHBI-1T final concentration at 28.0 μL final volumes per well in flat bottom, black polystyrene, non-binding surface 96-well half-area microplates (Corning 3686). Three microliters of either 132 µM m12, 122 µM m14, 101 µM m16, or 84.9 µM m17 in high-salt Tev cleavage buffer was mixed with 3.00 µL of 150 µM DFHBI-1T in high-salt Tev cleavage buffer. For each split mFAP fragment, 12.0 µL of the complementary split mFAP fragment in high-salt Tev cleavage buffer (the titrant) was mixed in from eleven serial dilutions ($\sqrt {10}$ dilution factor) starting from 422 µM m38, 33.0 µM m58, 348 µM m78, or 531 µM m8 stock solutions, respectively, including a twelfth condition without titrant. Fluorescence endpoints were measured on a Synergy Neo2 hybrid multi-mode reader (BioTek) using excitation wavelength *λ*_ex_ = 468 nm and emission wavelength *λ*_em_ = 530 nm. For each titration, the fluorescence intensity of the condition without titrant was subtracted from the fluorescence intensities of samples containing titrant, then the background-subtracted data was normalized from 0 to 1. In collecting fluorescence excitation and emission spectra (Supplementary Fig. 20c), the conditions with the highest protein concentrations and 25.0 µM DFHBI-1T were used. Excitation spectra were measured using excitation wavelengths in the range *λ*_ex_ = 350–498 nm and emission wavelength *λ*_em_ = 530 nm, and emission spectra were measured using excitation wavelength *λ*_ex_ = 468 nm and emission wavelengths in the range *λ*_em_ = 500–650 nm. Fluorescence excitation and emission spectra of conditions without the addition of the complementary split mFAP fragment were measured and used for background subtraction at the corresponding wavelengths.

For titrating BCLXL_m58 into m14_aBCLXL (Fig. 6b), m14_aBCLXL and BCLXL_m58 were prepared by large-scale protein purification^49^ in high-salt Tev cleavage buffer. Fluorescence endpoints were measured on a Synergy Neo2 hybrid multi-mode reader (BioTek) in flat bottom, black polystyrene, non-binding surface 384-well microplates (Corning 4514) using fluorescence excitation wavelength *λ*_ex_ = 468 nm and fluorescence emission wavelength *λ*_em_ = 530 nm. Nine wells each with 3.90 µL of 19.6 µM m14_aBCLXL and 2.20 µL of 114 µM DFHBI-1T were prepared, and 3.90 µL of either high-salt Tev cleavage buffer or BCLXL_m58 was aliquoted per well to reach final concentrations of 0 µM, 251 nM, 501 nM, 1.00 µM, 2.01 µM, 4.01 µM, 8.02 µM, 16.0 µM, or 32.1 µM BCLXL_m58, with 25.0 µM DFHBI-1T and 7.64 µM m14_aBCLXL in 10.0 µL final volumes per well. Fluorescence intensities were measured after 2847 s of double orbital shaking in the dark. Fluorescence from the 0 µM BCLXL_m58 condition was subtracted from each condition, and the background-subtracted fluorescence in relative fluorescence units (RFU), *F*, was normalized by the formula:

4 $${\mathrm{Norm}}{\mathrm{.Fluorescense}} = \frac{{F - F_{{\mathrm{min}}}}}{{F_{{\mathrm{max}}} - F_{{\mathrm{min}}}}},$$

where *F*_min_ (RFU) was the minimum fluorescence intensity, and *F*_max_ (RFU) was the fit to a constant function using non-linear least squares fitting of the fluorescence intensities of the four highest BCLXL_m58 concentrations. Using a bimolecular association model:

5 $$ {{\mathrm{BCLXL}}\_{\mathrm{m}}58} + {{\mathrm{m}}14\_{\mathrm{aBCLXL}}} \, \mathop { \rightleftharpoons }\limits_{k_2}^{k_1} \, {{\mathrm{BCLXL}}\_{\mathrm{m}}58 {\hbox{--}} {\mathrm{m}}14\_{\mathrm{aBCLXL}}} ,$$

it can be shown that:

6 $$\begin{array}{l} [{\mathrm{BCLXL}}\_{\mathrm{m}}58{\hbox{--}}{\mathrm{m}}14\_{\mathrm{aBCLXL}}] = 0.5 \cdot \left( {[{\mathrm{BCLXL}}\_{\mathrm{m}}58]_{{\mathrm{total}}} + [{\mathrm{m}}14\_{\mathrm{aBCLXL}}]_{{\mathrm{total}}} + K_{\mathrm{d}}} \right)\\ - \, 0.5 \cdot \sqrt {\left( { - [{\mathrm{BCLXL}}\_{\mathrm{m}}58]_{{\mathrm{total}}} - [{\mathrm{m}}14\_{\mathrm{aBCLXL}}]_{{\mathrm{total}}} - K_{\mathrm{d}}} \right)^2 \,- \, \left( {4 \cdot [{\mathrm{BCLXL}}\_{\mathrm{m}}58]_{{\mathrm{total}}} \cdot [{\mathrm{m}}14\_{\mathrm{aBCLXL}}]_{{\mathrm{total}}}} \right)} \end{array},$$

where $\frac{{k_2}}{{k_1}} = K_{\mathrm{d}} = K_{\mathrm{d}}^{{\mathrm{BCLXL}}\_{\mathrm{m}}58-{\mathrm{m}}14\_{\mathrm{aBCLXL}}}$. The theoretical maximum fluorescent complex concentration, $[{\mathrm{BCLXL}}\_{\mathrm{m}}58{\hbox{--}}{\mathrm{m}}14\_{\mathrm{aBCLXL}}]_{{\mathrm{max}}}$, is reached at excess $[{\mathrm{BCLXL}}\_{\mathrm{m}}58]_{{\mathrm{total}}}$, taken at $[{\mathrm{BCLXL}}\_{\mathrm{m}}58]_{{\mathrm{excess}}} = 10.0\,{\mathrm{M}}$. Similarly, it can also be shown that:

7 $$\begin{array}{l}[{\mathrm{BCLXL}}\_{\mathrm{m}}58{\hbox{--}}{\mathrm{m}}14\_{\mathrm{aBCLXL}}]_{{\mathrm{max}}} = 0.5 \cdot \left( {[{\mathrm{BCLXL}}\_{\mathrm{m}}58]_{{\mathrm{excess}}} + [{\mathrm{m}}14\_{\mathrm{aBCLXL}}]_{{\mathrm{total}}} + K_{\mathrm{d}}} \right)\\ - \, 0.5 \cdot \sqrt {\left( { - [{\mathrm{BCLXL}}\_{\mathrm{m}}58]_{{\mathrm{excess}}} - [{\mathrm{m}}14\_{\mathrm{aBCLXL}}]_{{\mathrm{total}}} - K_{\mathrm{d}}} \right)^2 \,- \,\left( {4 \cdot [{\mathrm{BCLXL}}\_{\mathrm{m}}58]_{{\mathrm{excess}}} \cdot [{\mathrm{m}}14\_{\mathrm{aBCLXL}}]_{{\mathrm{total}}}} \right)} \end{array}.$$

As fluorescent complexes only form with the folded fraction of m14_aBCLXL, *p*_folded_, under the condition that $[{\mathrm{m}}14\_{\mathrm{aBCLXL}}]_{{\mathrm{folded}}} = p_{{\mathrm{folded}}} \cdot {7.64\ μ{\mathrm{{M}}}}\ {\mathrm{{is}}}\ {\mathrm{{the}}}\ [{\mathrm{m}}14\_{\mathrm{aBCLXL}}]_{{\mathrm{total}}}$, we fit *p*_folded_ as a free parameter to the normalized fluorescence intensity with the formula:

8 $$\frac{{F - F_{{\mathrm{min}}}}}{{F_{{\mathrm{max}}} - F_{{\mathrm{min}}}}} = \frac{{[{\mathrm{BCLXL}}\_{\mathrm{m}}58{\hbox{--}}{\mathrm{m}}14\_{\mathrm{aBCLXL}}]}}{{[{\mathrm{BCLXL}}\_{\mathrm{m}}58{\hbox{--}}{\mathrm{m}}14\_{\mathrm{aBCLXL}}]_{{\mathrm{max}}}}},$$

where:

9 $$K_{\mathrm{d}}^{{\mathrm{BCLXL}}\_{\mathrm{m}}58-{\mathrm{m}}14\_{\mathrm{aBCLXL}}} = K_{\mathrm{d}}^{{\mathrm{BCLXL}}-{\mathrm{aBCLXL}}} \cdot K_{\mathrm{d}}^{{\mathrm{m}}14-{\mathrm{m}}58} = 1.23 \cdot {10^{-13}}\,{\mathrm{M}},$$

because the aBCLXL domain of m14_aBCLXL associates with the binding cleft of the BCLXL domain of BCLXL_m58 with the previously reported^37^ BCLXL–aBCLXL thermodynamic dissociation constant of $K_{\mathrm{d}}^{{\mathrm{BCLXL}}-{\mathrm{aBCLXL}}}$ = $5.59 \cdot 10^{ - 9}\,{\mathrm{M}}$, and the m14 domain of m14_aBCLXL associates with the m58 domain of BCLXL_m58 with the m14–m58 thermodynamic dissociation constant taken as $K_{\mathrm{d}}^{{\mathrm{m}}14-{\mathrm{m}}58} = 22.0 \cdot 10^{ - 6}\,{\mathrm{M}}$ in 25.0 µM DFHBI-1T (Supplementary Fig. 20e), under an approximation that the BCLXL_m58–m14_aBCLXL interaction energy comprises only the BCLXL–aBCLXL and m14–m58 interaction energies:

10 $${\Delta} {\mathrm{G}}^{{\mathrm{BCLXL}}\_{\mathrm{m}}58-{\mathrm{m}}14\_{\mathrm{aBCLXL}}} = {\Delta} {\mathrm{G}}^{{\mathrm{BCLXL}}-{\mathrm{aBCLXL}}} + {\Delta} {\mathrm{G}}^{{\mathrm{m}}14-{\mathrm{m}}58},$$

where ${\Delta} {\mathrm{G}}$ is the change in Gibbs free energy upon the superscripted protein–protein interaction in 25.0 µM DFHBI-1T. Non-linear least squares fitting yields $p_{{\mathrm{folded}}} = 0.532 \pm 0.0160$, and therefore the reported $[{\mathrm{m}}14\_{\mathrm{aBCLXL}}]_{{\mathrm{folded}}}$ = 4.06 µM (Fig. 6b). The error estimate is s.d. of the fit.

### Split mFAP temporal assays

In temporally monitoring fluorescence intensities (Fig. 6d), fluorescence was measured on a Synergy Neo2 hybrid multi-mode reader (BioTek) in flat bottom, black polystyrene, non-binding surface 96-well microplates (Corning 3650) using excitation wavelength *λ*_ex_ = 468 nm and emission wavelength *λ*_em_ = 530 nm. aBCL2, aBFL1, aBCLXL, m14_aBCL2, m14_aBFL1, m14_aBCLXL, BCL2_m58, BFL1_m58, and BCLXL_m58 were prepared by large-scale protein purification^49^ in high-salt Tev cleavage buffer. Two wells each with 36.0 µL of either aBCL2, aBFL1, or aBCLXL, 36.0 µL of either BCL2_m58, BFL1_m58, or BCLXL_m58, and 12.0 µL of 250 µM DFHBI-1T were prepared with matched cognate-binding partners, and samples were mixed by double orbital shaking at room temperature for 30 min in the dark. Subsequently, 36.0 µL of high-salt Tev cleavage buffer was aliquoted into the first of the two wells (negative control group), and 36.0 µL of either m14_aBCL2, m14_aBFL1, or m14_aBCLXL was aliquoted into the second of the two wells (experimental group) with matched cognate-binding partners, respectively. Fluorescence intensities were measured every 30 s between 5 s double orbital shake steps to mix the samples for 1200 s. Final sample conditions were 2.79 µM of aBCL2, m14_aBCL2, and BCL2_m58, 2.48 µM of aBFL1, m14_aBFL1, and BFL1_m58, 3.88 µM of aBCLXL, m14_aBCLXL, and BCLXL_m58, and 25.0 µM DFHBI-1T for all conditions in 120 µL final volumes per well. For each condition, fluorescence fold-change was calculated as:

11 $$\frac{{{\Delta} F}}{{F_0}} = \frac{{F - {F_0}}}{{F_0}},$$

where *F* (RFU) is the fluorescence intensity per measurement and *F*_0_ (RFU) is the fluorescence intensity of the first measurement, then fluorescence fold-change was fit to a monophasic exponential function using non-linear least squares fitting (Fig. 6d). In collecting fluorescence excitation and emission spectra after reaching equilibrium (Supplementary Fig. 21a), fluorescence excitation spectra were measured using excitation wavelengths in the range *λ*_ex_ = 350–530 nm and emission wavelength *λ*_em_ = 562 nm, and emission spectra were measured using excitation wavelength *λ*_ex_ = 438 nm and emission wavelengths in the range *λ*_em_ = 470–650 nm, and the normalized spectra reported without background subtraction.

In temporally monitoring fluorescence intensities (Fig. 6f), fluorescence was measured on a Synergy Neo2 hybrid multi-mode reader (BioTek) in flat bottom, black polystyrene, non-binding surface 96-well half-area microplates (Corning 3686) using excitation wavelength *λ*_ex _ = 478 nm and emission wavelength *λ*_em_ = 530 nm. m14_aBFL1, BCL2_m58, and aBCL2 were prepared by large-scale protein purification^49^ in high-salt Tev cleavage buffer. Three wells of 2.22 µM m14_aBFL1 with 2.22 µM BCL2_m58 and 27.8 µM DFHBI-1T in high-salt Tev cleavage buffer at final volumes of 45.0 µL were prepared and mixed by double orbital shaking at room temperature for 20 min in the dark. Subsequently, 5.00 µL of either 100 µM aBCL2, 40.0 µM aBCL2, or high-salt Tev cleavage buffer was aliquoted per well, respectively, and fluorescence intensities measured every 12 s between 5 s double orbital shake steps to mix the samples for 2,604 s. Final sample conditions were 25.0 µM DFHBI-1T, 2.00 µM m14_aBFL1, 2.00 µM BCL2_m58 and either 10.0 µM, 4.00 µM, or 0 µM aBCL2 in 50.0 µL final volumes per well. For each condition, fluorescence fold-change was calculated by Eq. (11) where *F* (RFU) is the fluorescence intensity per measurement and *F*_0_ (RFU) is the fluorescence intensity of the first measurement, then fluorescence fold-change was fit to a monophasic exponential function using non-linear least squares fitting (Fig. 6f). In collecting fluorescence excitation and emission spectra after reaching equilibrium (Supplementary Fig. 21b), fluorescence excitation spectra were measured using excitation wavelengths in the range *λ*_ex_ = 350–530 nm and emission wavelength *λ*_em_ = 570 nm, and fluorescence emission spectra were measured using excitation wavelength *λ*_ex_ = 430 nm and emission wavelengths in the range *λ*_em_ = 470–750 nm, and the normalized spectra reported without background subtraction.

### Circular dichroism

Circular dichroism (CD) measurements were recorded at 25 °C in a 1 mm cuvette on an AVIV model 420 CD spectrometer (Biomedical, Inc.). mFAP2 and mFAP2.1 were purified by large-scale protein purification and SEC purification^49^ in phosphate-buffered saline (PBS) (25.0 mM phosphate, 150 mM NaCl, pH 7.40), and far-ultraviolet CD wavelength scans recorded from 195 nm to 260 nm. mFAP2 was measured at 0.441 mg mL^−1^ and mFAP2.1 at 0.500 mg mL^−1^ in Na_2_HPO_4_-citrate buffer with pH adjusted to 7.75, 3.96, or 2.93 using NaOH and HCl. The reported data was background-subtracted using buffer only contols (Supplementary Fig. 1b, c).

EF4n_mFAP2b (Supplementary Fig. 8) was purified by large-scale protein purification and SEC purification^49^ in Dulbecco’s Phosphate-Buffered Saline without calcium or magnesium (DPBS) (Thermo Scientific). A small amount of Chelex 100 was added to the protein sample and nutated overnight at 4 °C. A stock solution of 1.00 mM CaCl_2_ was prepared in DPBS pre-treated with a small amount of Chelex 100 overnight. Far-ultraviolet CD wavelength scans and thermal denaturations were performed with 0.500 mg mL^−1^ protein in either 100 µM CaCl_2_ or DPBS, both using Chelex 100 pre-treated DPBS. Far-ultraviolet CD wavelength scans from 195 nm to 260 nm were recorded at 25 °C, and thermal denaturation was monitored at 220 nm wavelength from 25 °C to 95 °C at 2 °C evenly spaced intervals. Reported data are background-subtracted from the corresponding 100 μM CaCl_2_ or DPBS buffer measurements without protein.

### Quantum yield measurements

*Protein preparation:* mFAP2a, mFAP2b, and mFAP10 were produced by large-scale protein purification and SEC purification^49^, and dialyzed overnight into DPBS that was adjusted to pH 7.40 using NaOH.

*Chromophore preparation*: DFHBI (Lucerna) and DFHBI-1T (Lucerna) were dissolved to 20.0 mM in 100% DMSO, and diluted in DPBS (pH 7.40) to measure absorbances on a Jasco V-750 spectrophotometer at peak absorbance wavelengths (417 nm for DFHBI and 422 nm for DFHBI-1T). Following background subtraction of identical buffer without chromophore, Beer’s Law was used to calculate the molar chromophore concentrations of the stock solutions using previously reported extinction coefficients^4^.

*Preparation of protein–chromophore complexes*: For quantum yield measurements, 1.00 μM, 836 nM, or 919 nM chromophore solutions in DPBS (pH 7.40) at 4.00 mL final volumes were prepared for the following eight conditions: DFHBI only, DFHBI-1T only, 43.5 µM 6xHis-mFAP10 with DFHBI, 43.5 µM 6xHis-mFAP10 with DFHBI-1T, 134 μM 6xHis-mFAP2a with DFHBI, 134 μM 6xHis-mFAP2a with DFHBI-1T, 206 μM 6xHis-mFAP2b with DFHBI, and 206 μM 6xHis-mFAP2b with DFHBI-1T.

*Extinction coefficients*: Absorbance spectra of protein–chromophore complexes were first measured with a Thermo Scientific BioMate 3S UV–Vis Spectrophotometer (1 nm interval, 800 nm min^−1^). The extinction coefficients were then calculated using Beer’s Law:

12 $$A = \varepsilon \cdot b\cdot c,$$

where *A* is peak absorbance, *ε* is extinction coefficient, *b* is path length (1 cm), and *c* is concentration (1.00 μM, 836 nM, or 919 nM).

*Relative quantum yield*: A Perkin-Elmer LS-B Luminescence Spectrophotometer (10 nm bandwidth, 1 nm interval, 100 nm min^−1^) was used. The fluorescence emission spectra of the protein–chromophore complexes (in DPBS, pH 7.40) and reference dye Acridine Yellow G (in methanol) were first obtained, and the quantum yield was then calculated using the equation^53^:

13 $$\phi _{\mathrm{c}} = \phi _{\mathrm{r}}\cdot \frac{{1 - 10^{ - A_{\mathrm{r}}(\lambda _{{\mathrm{ex}}})}}}{{1 - 10^{ - A_{\mathrm{c}}(\lambda _{{\mathrm{ex}}})}}}\cdot \frac{{ {\int }{F_{\mathrm{c}}}(\lambda ) \cdot d\lambda }}{{{\int } {F_{\mathrm{r}}}(\lambda ) \cdot d\lambda }}\cdot \frac{{n_{\mathrm{c}}^2}}{{n_{\mathrm{r}}^2}},$$

where *ϕ* is quantum yield, $A(\lambda _{{\mathrm{ex}}})$ is absorbance at the excitation wavelength $\lambda _{{\mathrm{ex}}}$ ($\lambda _{{\mathrm{ex}}}$ = 440 nm), *F* is fluorescence emission, *n* is refractive index of the solution (1.3350 for DPBS at pH 7.40 and 1.3284 for methanol), and the subscripts “c” and “r” refer to the protein–chromophore complex measured and the reference dye, respectively. The reference dye Acridine Yellow G (in methanol) has a quantum yield value of 0.57 that was used^54^.

*Absolute quantum yield*: An integrating sphere instrument (Hamamatsu C9920-12) (6 nm excitation bandwidth, 1 nm interval) and a high-sensitivity photonic multi-channel analyzer (Hamamatsu C10027-01) were used to measure a light emission spectrum. Absolute quantum yields were measured for solutions of protein–chromophore complexes in DPBS (pH 7.40) in which ≥95% of the total chromophore was occupying the protein-binding pocket (Table 1). Protein–chromophore complex samples and control samples were excited at *λ*_ex_  = 440 nm and absolute quantum yields were calculated according to the equation:

14 $$\phi _{\mathrm{c}} = \frac{{f_{{\mathrm{em}}}}}{{f_{{\mathrm{abs}}}}},$$

where *f*_em_ is the emitted photon flux and *f*_abs_ is the absorbed photon flux. The absolute quantum yields of the two control samples (Acridine Yellow G and fluorescein) agreed well with literature values^54,55^. Absolute quantum yield data was analyzed with U6039-05 PLQY measurement software.

### Size-exclusion chromatography with multi-angle light scattering

Protein samples were prepared at 2.0 mg mL^−1^ and applied to a Superdex 75 10/300 GL column (GE Healthcare) on a LC 1200 Series HPLC machine (Agilent Technologies) for size-based separation, a Heleos detector (Wyatt Technologies) for light scattering signals, and a t-Rex detector for differential refractive index detection. Results were analyzed using ASTRA 7.2 software for weighted average molecular weight.

### COS-7 cell culture and transfection

COS-7 cells (ATCC CRL-1651) were cultured in Dulbecco’s modified Eagle’s medium (DMEM) supplemented with 1x NEAA, 100 units mL^−1^ penicillin, 100 µg mL^−1^ streptomycin, and 10% fetal bovine serum (FBS). For transfection, cells were collected using 0.25% trypsin EDTA, and approximately 1 million cells transfected by nucleofection using 2 µg of plasmid DNA (Supplementary Data 10), 18.0 µL of Lonza s.e. cell supplement, 82.0 µL of Lonza s.e. nucleofection solution, and pulse code DS-120 on a Lonza 4D X Nucleofector system. Cells were seeded into ibidi µ-Slide 8-well glass bottom chambers at a density of approximately 30,000 cells well^−1^ and recovered overnight at 37 °C.

### COS-7 cell fixation

COS-7 cells were treated at 37 °C with PFA/GA fixation solution (containing 100 mM aqueous PIPES buffer at pH 7.0, 1 mM MgCl_2_, 3.2% paraformaldehyde, and 0.1% gluteraldehyde) for 10 min, reduced with 10 mM aqueous sodium borohydride for 10 min, then rinsed with 1x PBS (11.9 mM phosphates, 137 mM NaCl, 2.70 mM KCl, pH 7.40) (Fisher Scientific #BP399-1) for 5 min (Supplementary Fig. 4).

### Epifluorescence microscopy of COS-7 cells

Conventional widefield epifluorescence imaging of cultured live COS-7 cells (Supplementary Movie 1 and Supplementary Movie 2) and fixed COS-7 cells (Supplementary Fig. 4) was performed on an inverted Nikon Ti-S microscope configured with a 60x/1.2 NA water-immersion objective lens (Nikon), a multiband filter set (LF405/488/532/635-A-000, Semrock), and a Zyla 5.5 sCMOS camera (Andor). Micro-Manager software with MM Studio and MMCore were used for acquisition. For widefield epifluorescence microscopy of fixed COS-7 cells expressing mFAP2a or mFAP2b targeted to the endoplasmic reticulum (ER) (Supplementary Fig. 4), samples were labeled with either 40.0 µM DFHBI or 40.0 µM DFHBI-1T in 1x PBS for at least 10 min before imaging. Cells were rinsed three times with 1.00 mL of 1x PBS. Samples were illuminated with 470 nm light at an intensity of ~2 W cm^−2^. Exposure times were 200 ms and current was 500 mA. For time-lapse widefield epifluorescence microscopy of live COS-7 cells expressing mFAP2a or mFAP2b targeted to the ER (Supplementary Movie 1, Supplementary Movie 2), cells were labeled with 40.0 µM DFHBI in 1x PBS. The time-lapse movies were acquired using 200 ms exposure times every 5 s for 25 total frames, with 100 mA excitation current. The total acquisition duration per movie was just over 2 min, and movie playback speeds adjusted to 5 frames s^−1^.

### Photostability assay

COS-7 cells transfected with either pcDNA5/FRT/TO-AcGFP1-sec61β, pcDNA5/FRT/TO-mFAP2a-sec61β, or pcDNA5/FRT/TO-mFAP2b-sec61β (Supplementary Data 10) were fixed and imaged (Fig. 2) at 25 °C using a Zeiss LSM-510 laser scanning confocal fluorescence microscope equipped with a Plan-Apochromat 63x/1.4 NA oil DIC objective lens and SP1 software. The Argon/2 488 nm excitation laser was set to 50% output power (4.0 A tube current) and at 10% transmission resulting in 10.3 µW laser power. The pinhole size was set to 98 μm (1.02 Airy units). The excitation laser source passed through a HFT488 dichroic beam splitter to the specimen, and fluorescence captured through the HFT488 dichroic beam splitter to a 505 nm long-pass emission filter to the detector. Acquiring 740 × 740 pixel (71.43 µm^2^) images with a single laser scan direction and 12-bit pixel depth resulted in a 1.13 s scantime and 0.8 μs pixeltime for photostability assays. For all samples, amplifier offset was set to −1.0 and amplifier gain set to 1.0. For samples expressing AcGFP1-sec61β, detector gain was set to 500. For samples expressing mFAP2a-sec61β, detector gain was set to 800 for 50.0 μM DFHBI labeling, 700 for 50.0 μM DFHBI-1T labeling, and 900 for 500 nM DFHBI and 500 nM DFHBI-1T labeling. For samples expressing mFAP2b-sec61β, detector gain was set to 700 for 50.0 μM DFHBI labeling and 900 for 500 nM DFHBI labeling. Fixed COS-7 cells expressing AcGFP1-sec61β were imaged in Tris buffered saline (TBS) (25.0 mM Tris, 300 mM NaCl, pH 8.00), and those expressing mFAP2a-sec61β and mFAP2b-sec61β were washed ten times with 50.0 μL of high-salt Tev cleavage buffer, and labeled with chromophore in high-salt Tev cleavage buffer for at least 10 min prior to imaging. Raw “*.lsm” data files were analyzed using linear lookup tables covering the full range of data (Fig. 2a, b). For each region of interest encompassing one or more fixed cells, normalized image intensity (Fig. 2c, d) was calculated by summing image pixel intensities per frame and dividing by the summed image pixel intensities of the first frame. Pixel intensities at the bit depth of the microscope detector were discounted. Non-linear least squares fitting of the average normalized image intensities to a monophasic exponential decay function of the form $F = h + (1 - h) \cdot {\mathrm{e}}^{ - k \cdot t}$ was used to obtain *h* constants, the percentage at which the normalized average image intensities asymptotically plateau. The ratio of *h* constants relative to AcGFP1 were reported as improved photostability over AcGFP1.

### Laser scanning confocal fluorescence microscopy of *E. coli*

Lemo21(DE3) *E. coli* cultures expressing either mFAP2a or mFAP2b were induced at 500 µM IPTG final concentration for 4 h at 37 °C shaking at 250 rpm. For imaging separate cultures (Fig. 1f–i), 1.5% agarose pads supplemented with either 10.0 μM DFHBI or 10.0 μM DFHBI-1T final concentrations were each molded using six stacked microscope slides on a leveled surface^56^. In all, 1.00 mL of induced cells was aliquoted from each culture, centrifuged, the pellet resuspended in 1.00 mL of high-salt Tev cleavage buffer, solution pelleted again, and the pellet resuspended in either 100 μL of 10.0 μM DFHBI or 100 μL of 10.0 μM DFHBI-1T (from 2.00 mM chromophore stock solutions in 5% DMSO and 95% high-salt Tev cleavage buffer), and incubated for 10 min at 25 °C. Two microliters of cells in chromophore solution were aliquoted onto ~1 cm^2^ agarose pads containing the corresponding chromophore, and the agarose pad placed in μ-Slide 4 Well chambers (ibidi) for imaging.

For imaging mixed cultures (Supplementary Fig. 3), induced *E. coli* cultures were diluted 10-fold in ddH_2_O and their optical density at 600 nm measured using a Genesys 10S UV–Vis spectrophotometer (Thermo Scientific). Proportional volumes of cell cultures expressing either mFAP2a or mFAP2b were mixed to achieve a 1:1 ratio of cells from each culture. One-hundred microliters of this mixture was centrifuged, the pellet resuspended in 200 μL of high-salt Tev cleavage buffer, solution pelleted again, and the pellet resuspended in either 10.0 μL of 10.0 μM DFHBI or 10.0 μL of 10.0 μM DFHBI-1T (from 2.00 mM chromophore stock solutions in 5% DMSO and 95% high-salt Tev cleavage buffer), and incubated for 10 min at 25 °C. Five microliters of each cellular mixture in different chromophores was pipetted onto frosted microscope slides (Fisher Scientific) between Premium Superslip glass coverslips (Fisher Scientific).

Images were acquired on a Zeiss LSM-510 laser scanning confocal fluorescence microscope equipped with a Plan-Apochromat 63x/1.4 NA oil DIC objective lens and SP1 software. The Argon/2 488 nm excitation laser was set to 50% output power (4.0 A tube current) and at 10% transmission resulting in 10.3 µW laser power. Pinhole size was set to 98 μm (1.02 Airy units), and fluorescence was captured through a 505 nm long-pass emission filter. Detector gain was set to 650, amplifier offset was set to −0.85, and amplifier gain set to 1.0. 740 × 740 pixel (71.43 µm^2^) images (Fig. 1f–i) or 1480 × 1480 pixel (142.86 µm^2^) images (Supplementary Fig. 3) were acquired with a single laser scan direction and 12-bit pixel depth. Raw “*.lsm” data files were analyzed using linear lookup tables covering the full range of data (Fig. 1f–i and Supplementary Fig. 3a, b). In Supplementary Fig. 3c, summed pixel intensities were calculated per image including the pixel intensities at the bit depth of the microscope detector.

### X-ray crystallography

EF1p2_mFAP2b was produced by large-scale protein purification, 6xHis-tag removal and SEC purification^49^ in TBS. Purified protein was mixed with excess DFHBI (resuspended in 100% DMSO), while keeping the final DMSO concentration <1%, followed by addition of 5.00 mM CaCl_2_ final concentration. The EF1p2_mFAP2b–DFHBI complex was then concentrated to ~25 mg mL^−1^, and initially tested for crystallization via sparse matrix screens in 96-well sitting drops using a mosquito (TTP LabTech). A single crystal was obtained in a 200 nL drop from 100 mM HEPES pH 7.50 and 25% PEG 3350 (Index, Hampton Research). The drop was flooded with reservoir solution plus 2.00 mM DFHBI and 20% ethylene glycol then flash frozen in liquid nitrogen. Data was collected with a home-source rotating anode on a Saturn 944+ CCD and processed in HKL2000^57^. For phasing and refinement, structures were solved by Molecular Replacement with Phaser via phenix^58,59^ using a mFAP2b design model from Rosetta^23^ with appropriate residue side-chains cut back to C_α_ atoms, and DFHBI and loop7 residues removed. The structure was then built and refined using Coot^60^ and phenix^61^, respectively (Supplementary Table 3).

### Molecular dynamics simulations

Chain B of the refined EF1p2_mFAP2b–DFHBI–Ca^2+^ co-crystal structure (PDB accession code 6OHH) was used as the starting point for molecular dynamics (MD) simulations. The other three system conditions were obtained by removing the coordinates of the Ca^2+^ ion and DFHBI ligand (Apo), or either that of the ion or of the ligand (DFHBI-bound and Ca^2+^-bound conditions, respectively). Missing side-chains were added using Schrödinger’s Maestro (version 10.4, Schrödinger, LLC, New York, NY) and all crystallographic waters were kept. Protonation states at pH 7 were assigned using Maestro’s PROPKA. The accessible protein cavities left by the removal of the ligand in the Apo and Ca^2+^-bound systems were hydrated with Dowser^62^. Proteins were solvated in TIP3P water boxes^63^ with a buffer distance of 16 Å to the box edges and NaCl ions were added to provide charge neutrality at a total concentration of 150 mM. The Amber14SB force field^64,65^ was used for the protein and NaCl. The DFHBI ligand was parametrized using Antechamber and the generalized Amber Force Field (GAFF)^66,67^, with geometry optimization performed with Gaussian 09^68^. Ca^2+^ parameters were obtained from Bradbrook et al.^69^.

The systems were minimized in five stages with increasing number of unconstrained atoms (proton only, solvent, ligand, side-chains, and the full system) totaling 13,000 steps of steepest descent and conjugate gradient methods. This was followed by equilibration involving an initial heating to 100 K at constant volume for 50 ps followed by heating to 298 K at a constant pressure of 1 bar for 200 ps. The systems were further equilibrated at 298 K and 1 bar for 2.25 ns. Production runs were performed using GPU accelerated Amber14^65,70^ at 1 bar and 298 K with periodic boundary conditions and a 2 fs timestep, with non-bonded short-range interactions evaluated within a cutoff of 10 Å. Each of the four system conditions were simulated in three independent 500 ns replicates. The first 100 ns of each production run were discounted from analysis to allow for adequate structural relaxation from the starting conformation. The trajectories were visualized in VMD^71^ and aligned to the co-crystal protein structure. The protein backbone atom coordinates were used to probe the conformational free energy landscape using PyEMMA^72^ and in-house scripts. The loop7 residue coordinates of all simulations were jointly clustered into 25 clusters, resulting in whole protein cluster centroids with an average backbone heavy atom root mean square deviation (RMSD) of 2.49 Å to each other. Clustering was performed on loop7 backbone coordinates using PyEMMA’s k_means algorithm, and each cluster centroid structure was defined as the structure, which minimized the sum of the RMSD values to all other cluster members. CPPTRAJ^73^ and MDTraj^74^ were used for RMSF and RMSD analysis.

### HEK293 cell transfections

Plasmid DNAs (Supplementary Data 15 and Supplementary Data 17) were purified in ddH_2_O for transfections. pGP-CMV-GCaMP6f was a gift from Douglas Kim & GENIE Project (Addgene plasmid #40755; [http://n2t.net/addgene:40755]; RRID: Addgene_40755). Wild-type HEK293 cells (ATCC CRL-1573) were cultured on 24-well plates in DMEM media (4.5 g L^−1^ d-glucose, l-glutamine; ThermoFisher #11965-092) supplemented with 10% FBS and 1% Penicillin/Streptomycin in a 5% CO_2_ atmosphere at 37 °C. Cells were seeded into 24-well plates (ThermoFisher #FB012929) at 100,000 cells well^−1^. Twenty-four hours after seeding, cell media was aspirated and replaced with 200 μL of DMEM media. Lipofectamine 3000 (ThermoFisher Scientific) reagents were prepared according to the manufacturer’s instructions by mixing 1.50 μL of Lipofectamine 3000 reagent diluted in 25.0 μL of OPTI-MEM with 1 μg of plasmid DNA diluted in 25.0 μL OPTI-MEM and 2.00 μL of P3000 reagent, allowing for formation of DNA–lipid complexes for ~10 min after combination of the two mixtures. Reagent volumes were increased for the number of wells to be transfected. Fifty microliters of complexed Lipofectamine reagents were pipetted into each well, cells were incubated for 30 min, and the volume in each well was raised to 750 μL with DMEM media. Approximately 6 h after transfection, media was replaced with 1.00 mL of DMEM media. Cells were incubated for 48 h to allow for protein expression.

### HEK293 cell surface-displayed Ca^2+^ titrations

*Ca*^*2+*^ *titration protocol*: A stock solution of 20.0 mM DFHBI was prepared in 100% anhydrous DMSO. Two days after the transfection of pDisplay-EF1p_mFAP2b, each 24-well plate well was rinsed twice with pre-warmed Ca^2+^-deficient Tyrode’s solution (124 mM NaCl, 2 mM KCl, 2 mM MgCl_2_, 10 mM HEPES and 10 mM glucose, pH between 7.3 and 7.4 with NaOH), then filled with 200 µL of the same solution containing 7.00 µM DFHBI (approximately $\left( {K_{\mathrm{d}}^ + \cdot K_{\mathrm{d}}^ - } \right)^{1/2}$ for EF1p_mFAP2b; Supplementary Table 2). Cells were incubated (5% CO_2_ at 37 °C) in the dark for 5 min before the Ca^2+^ titration to allow DFHBI to reach equilibrium with the sensor. Cells were imaged at 5 frames s^−1^ for 1 min with a sCMOS camera (Photometrics Prime95B) and a 20x magnification lens (Leica HCX PL FLUOTAR L 20x/0.40 NA CORR). The cells were continually illuminated at 7.65 mW cm^−2^ with a LumenCor Light Engine (Semrock filters: Excitation 474/27 nm; Emission 520/35 nm). Twenty seconds into the imaging experiment, a syringe pump (Harvard Apparatus Pump 11 Elite) was triggered by a TTL pulse. Two-hundred microliters of 20 mM Ca^2+^ Tyrode’s solution (84 mM NaCl, 2 mM KCl, 2 mM MgCl_2_, 20 mM CaCl_2_, 10 mM HEPES and 10 mM glucose, pH between 7.3–7.4 with NaOH) with the identical DFHBI concentration was added at a rate of 2 mL min^−1^ to the wells. In technical triplicate, the final conditions of each well were 400 μL volume and 10 mM Ca^2+^, with constant 7.00 µM DFHBI concentration throughout the experiment. “After Photobleaching” (see Fig. 5d) cells in a different region of interest (ROI) were illuminated for the same duration and imaging conditions used for each imaging experiment, prior to the titration experiment.

*Data analysis*: Regions of interest (ROIs) surrounding single cells from three technical replicates were hand-drawn in ImageJ software^75^. For each cellular ROI in each frame the average fluorescence was calculated in ImageJ. In order to perform background subtraction, cellular ROIs were then moved proximal to the original cell to an ROI where there was no fluorescence. Each cellular ROIs average fluorescence had the average background intensity subtracted for each frame. Following background subtraction, fluorescence fold-change was calculated by Eq. (11) where *F* is the background-subtracted average fluorescence per ROI per frame, and *F*_0_ is the background-subtracted average baseline fluorescence as measured 1 s prior to the Ca^2+^ titration^29^ (Fig. 5d).

### HEK293 cell acetylcholine stimulations

*Stimulation protocol*: A stock solution of 20.0 mM DFHBI was prepared in 100% anhydrous DMSO. Media was aspirated from the wells and the cells were rinsed once with 200 μL of Tyrode’s solution (120 mM NaCl, 2 mM KCl, 2 mM MgCl_2_, 2 mM CaCl_2_, 10 mM HEPES, 10 mM glucose, pH adjusted to 7.3–7.4 using NaOH). Cells were placed in 750 μL Tyrode’s solution supplemented with 20.0 μM DFHBI for EF2n_mFAP2a-expressing cells, 43.3 μM DFHBI for EF4n_mFAP2a-expressing cells, and 43.3 μM DFHBI for EF4n_mFAP2b-expressing cells. GCaMP6f-expressing cells were placed in 750 μL Tyrode’s solution. Cells were placed in the dark at 37 °C for 30 min prior to stimulation. Imaging was performed on a Leica DMI8 microscope controlled by MetaMorph Imaging software. Cells were imaged at 5 frames s^−1^ for 1 min with a sCMOS camera (Photometrics Prime95B) and 20x magnification lens (Leica HCX PL FLUOTAR L 20x/0.40 NA CORR). Ca^2+^-responsive mFAP-expressing cells were continually illuminated at 7.65 mW cm^−2^, and GCaMP6f-expressing cells were continually illuminated at 1.24 mW cm^−2^, using a LumenCor Light Engine (Semrock filters: Excitation 474/27 nm; Emission 520/35 nm). Twenty seconds into the imaging experiment, a syringe pump (Harvard Apparatus Pump 11 Elite) was triggered by a TTL pulse. One-hundred microliters of Tyrode’s solution spiked with the identical DFHBI concentration and percent DMSO, and 850 μM acetylcholine (ACh) was added at a rate of 2 mL min^−1^ to the wells. In technical triplicate per sensor, the final conditions of each well were 850 μL volume of 100 μM ACh with constant DFHBI concentration throughout the addition of the ACh stimulation solution.

*Data analysis*: Regions of interest (ROIs) surrounding single cells from three technical replicates were hand-drawn in ImageJ software^75^. For each cellular ROI in each frame the average fluorescence was calculated in ImageJ. In order to perform background subtraction, cellular ROIs were then moved proximal to the original cell to an ROI where there was no fluorescence. Each cellular ROIs average fluorescence had the corresponding background fluorescence subtracted for each frame. Following background subtraction, fluorescence fold-change was calculated by Eq. (11) where *F* is the background-subtracted average fluorescence per ROI per frame, and *F*_0_ is the background-subtracted average baseline fluorescence as measured 1 s prior to ACh stimulation^29^. A maximal fluorescence response was determined as the maximum absolute value of the fluorescence fold-change after the stimulation frame (the peak $| {\frac{{{\Delta} F}}{{F_0}}} |$; Table 2), and a two-sided Wilcoxon rank sum test was used to compare values (Supplementary Fig. 18).

### Cardiomyocyte imaging

*hiPSC culture and cardiomyocyte differentiation*: Undifferentiated IMR90 human induced pluripotent stem cells (hiPSCs) (WiCell) were maintained in mTeSR-1 (StemCell Technologies) on tissue culture plastic coated with Matrigel diluted 1:60 (Corning) at 37 °C and 5% CO_2_. Cardiomyocytes were differentiated using a monolayer-based directed differentiation protocol^76^. hiPSCs were dissociated into a single cell suspension using Versene (Life Technologies) and plated at a high density (1.5–2.5·10^5^ cells cm^−2^) in mTeSR containing 10 µM Y-27632 ROCK inhibitor onto Matrigel-coated plates. Twenty-four hours after plating, media was replaced with fresh mTeSR. Forty-eight hours after seeding, differentiation was induced by changing the media to 10–12 µM CHIR 99021 (Stemgent) in RPMI 1640 supplemented with B27 minus insulin (RPMI-ins, ThermoFisher). After 24 h post-induction, the media was changed to fresh RPMI-ins. At 3 days post-induction, the media was changed to 3–5 µM IWP4 (Stemgent) in RPMI-ins. At 5 days post-induction, the media was changed to fresh RPMI-ins. At 7 days post-induction, the media was changed to RPMI medium containing B27 supplement with insulin (RPMI+ins), and media was changed every other day. Cardiomyocytes (CMs) were replated at day 14 post-induction in RPMI+ins at 1.5·10^5^ cells cm^−2^. CMs were then purified via metabolic challenge by culturing in DMEM without glucose or sodium pyruvate, supplemented with 4 mM sodium l-lactate for 4 days^77^.

*Recombinant adeno-associated virus (rAAV) production and infection of hiPSC CMs*: AAV-CAG-hChR2-H134R-tdTomato was a gift from Karel Svoboda (Addgene plasmid # 28017; [http://n2t.net/addgene:28017]; RRID: Addgene_28017), the vector backbone into which the EF1n_mFAP2b gene with sarcoplasmic reticulum (SR) targeting sequence was sub-cloned (Supplementary Data 16). rAAV serotype 6 (rAAV6) were produced^78^ with a titer for rAAV6-SR-EF1n_mFAP2b of 2.90·10^13^ viral genomes mL^−1^. Purified hiPSC CMs in 6-well plates were infected with 3.00 µL of the EF1n-mFAP2b rAAV6 construct at 30 days post-induction. Cardiomyocytes were incubated for 5 days before imaging. RPMI+ins media was replenished every 2 days without exchanging media.

*Contraction and SR Ca*^*2+*^ *transient imaging*: Leica DMI8 microscope controlled by MetaMorph Imaging software was used to image contraction and Ca^2+^ transients of the SR in CMs. Prior to imaging experiments, CMs were rinsed with Tyrode’s solution and the 6-well plate wells were filled with 2.0 mL of Tyrode’s solution with 6.00 µM DFHBI (Supplementary Fig. 19b) or 3.00 µM DFHBI (Fig. 5f) from the stock solution. Cardiomyocytes were imaged at ~16.7 Hz (60 ms frame^−1^) for up to 24 s with a sCMOS camera (Photometrics Prime95B) and 20x magnification lens (Leica HCX PL FLUOTAR L 20x/0.40 NA CORR). The cells were continually illuminated at 5.72 mW cm^−2^ (Supplementary Fig. 19b) or 15.8 mW cm^−2^ (Fig. 5f and Supplementary Fig. 19c) with a LumenCor Light Engine (Semrock filters: Excitation 474/27 nm; Emission 520/35 nm). For mapping contraction, three different ROI traces were hand-drawn, and displacement of cells were measured in the fluorescence channel. The normalized mean of the three ROI traces is plotted to visualize contraction of cardiomyocytes (Fig. 5f and Supplementary Fig. 19b). In order to quantify tissue-level Ca^2+^ fluxes during cardiac contraction cycling, fluorescence fold-change over the whole field of view was calculated for each frame then temporally matched with contraction data. Fluorescence fold-change in each frame was calculated by Eq. (11) where *F* is the fluorescence per frame, and *F*_0_ is the fluorescence of the frame with the minimum fluorescence (Fig. 5f and Supplementary Fig. 19b). Temporal analysis was performed over 20 cardiac contraction cycles by averaging for each contraction the temporal difference between the peak whole field of view normalized fluorescence fold-change and the peak normalized average fluorescence from three ROI traces in the fluorescence channel (Fig. 5f).

*Pharmacological inhibition of SERCA Ca*^*2+*^ *pumps*: Cardiomyocytes in 2.0 mL of Tyrode’s solution labeled with 3.00 µM DFHBI were imaged at 2 Hz for 30 min with an sCMOS camera (Photometrics Prime95B) and 20x magnification lens (Leica HCX PL FLUOTAR L 20x/0.40 NA CORR). A stock solution of 20.0 mM cyclopiazonic acid (CPA) (Tocris, 1235) in 100% anhydrous DMSO was prepared. Two milliliters of Tyrode’s solution containing 3.00 µM DFHBI and 40.0 µM CPA was administered at ~10 min from initial image acquisition to inhibit SERCA pumps and disrupt Ca^2+^ recovery into the SR after contraction^36^. Fluorescence fold-change over the whole field of view in each frame was calculated by Eq. (11), where *F* is the fluorescence per frame, and *F*_0_ is the fluorescence at the first frame (Supplementary Fig. 19c).

### Reporting summary

Further information on research design is available in the Nature Research Reporting Summary linked to this article.

## Supplementary information

Supplementary Information

Supplementary Data 1

Supplementary Data 2

Supplementary Data 3

Supplementary Data 4

Supplementary Data 5

Supplementary Data 6

Supplementary Data 7

Supplementary Data 8

Supplementary Data 9

Supplementary Data 10

Supplementary Data 11

Supplementary Data 12

Supplementary Data 13

Supplementary Data 14

Supplementary Data 15

Supplementary Data 16

Supplementary Data 17

Reporting Summary

Description of Additional Supplementary Files

Supplementary Movie 1

Supplementary Movie 2

Supplementary Movie 3

## Data Availability

The atomic coordinates and experimental data of the EF1p2_mFAP2b–DFHBI–Ca^2+^ co-crystal structure have been deposited in the RCSB Protein Data Bank with the accession code 6OHH. Time-lapse widefield epifluorescence microscopy movies of live COS-7 cells are available in Supplementary Movie 1 and Supplementary Movie 2. The normalized time-lapse widefield epifluorescence microscopy movie of live hiPSC-derived CMs expressing SR-targeted EF1n_mFAP2b labeled at 3.00 µM DFHBI is available in Supplementary Movie 3. Amino acid sequences of mFAP variants are reported in Supplementary Data 1 and Supplementary Data 2. Other amino acid, DNA and oligonucleotide sequences used throughout this research are reported in Supplementary Data 3 through Supplementary Data 17. Plasmid DNA that support the findings of this study are available at Addgene [www.addgene.org/David_Baker]. Source data are provided with this paper and online [10.5281/zenodo.3960743]. Other plasmid DNA and data that support the findings of this study are available from the corresponding author upon reasonable request. Computational models for mFAP2a, mFAP2b, mFAP10, the 59 extended loop decoys, 5 refined extended loop7 decoys, 8 circularly permuted mFAP2a or mFAP2b decoys, and 12 circularly permuted mFAP2a or mFAP2b decoys with designed linkers are available to download; the β-barrel loop fragment databases used to design the extended loop library, Supplementary Table 1, and Supplementary Table 2 are available to download [https://github.com/klimaj/mFAPs].

## Source data

Source Data

## Code availability

The Rosetta macromolecular modeling suite [https://www.rosettacommons.org] is freely available to academic/non-commercial users and commercial licenses are available via the University of Washington Technology Transfer Office. The Rosetta design and refinement protocols for the extended loop library are available in Supplementary Note 2. The sequence design protocol to generate the combinatorial library of linkers to graft one EF-hand motif onto loop7 of mFAP2b is available in Supplementary Note 3. The PyRosetta design protocol to generate circularly permuted mFAP2a and mFAP2b is available in Supplementary Note 4. The RosettaScripts script to design linkers onto cpmFAPs incorporates code in development and is available from the corresponding author upon request. Python scripts, RosettaScripts scripts, and parameterization files are available for download at [https://github.com/klimaj/mFAPs].
