# Supplementary material for: Incorporation of sensing modalities into de novo designed fluorescence-activating proteins
Source: Nat Commun. 2021 Feb 8;12:856. doi: 10.1038/s41467-020-18911-w (PMC7870846; doi:10.1038/s41467-020-18911-w)
Supplement: Supplementary file 1 — Supplementary Information [file 41467_2020_18911_MOESM1_ESM.pdf]

SUPPLEMENTARY INFORMATION

Incorporation of Sensing Modalities Into *De Novo* Designed Fluorescence-activating Proteins

Klima *et al.*

Table of Contents

Supplementary Figure 1.	4
Supplementary Figure 2.	7
Supplementary Figure 3.	8
Supplementary Figure 4.	9
Supplementary Figure 5.	10
Supplementary Figure 6.	11
Supplementary Figure 7.	13
Supplementary Figure 8.	14
Supplementary Figure 9.	15
Supplementary Figure 10.	17
Supplementary Figure 11.	18
Supplementary Figure 12.	19
Supplementary Figure 13.	20
Supplementary Figure 14.	21
Supplementary Figure 15.	23
Supplementary Figure 16.	24
Supplementary Figure 17.	25
Supplementary Figure 18.	28
Supplementary Figure 19.	31
Supplementary Figure 20.	32
Supplementary Figure 21.	34
Supplementary Figure 22.	35
Supplementary Figure 23.	37
Supplementary Table 1.	40
Supplementary Table 2.	41
Supplementary Table 3.	43
Supplementary Note 1.	44
Supplementary Note 2.	48
Supplementary Note 3.	54
Supplementary Note 4.	55
Supplementary References.	56

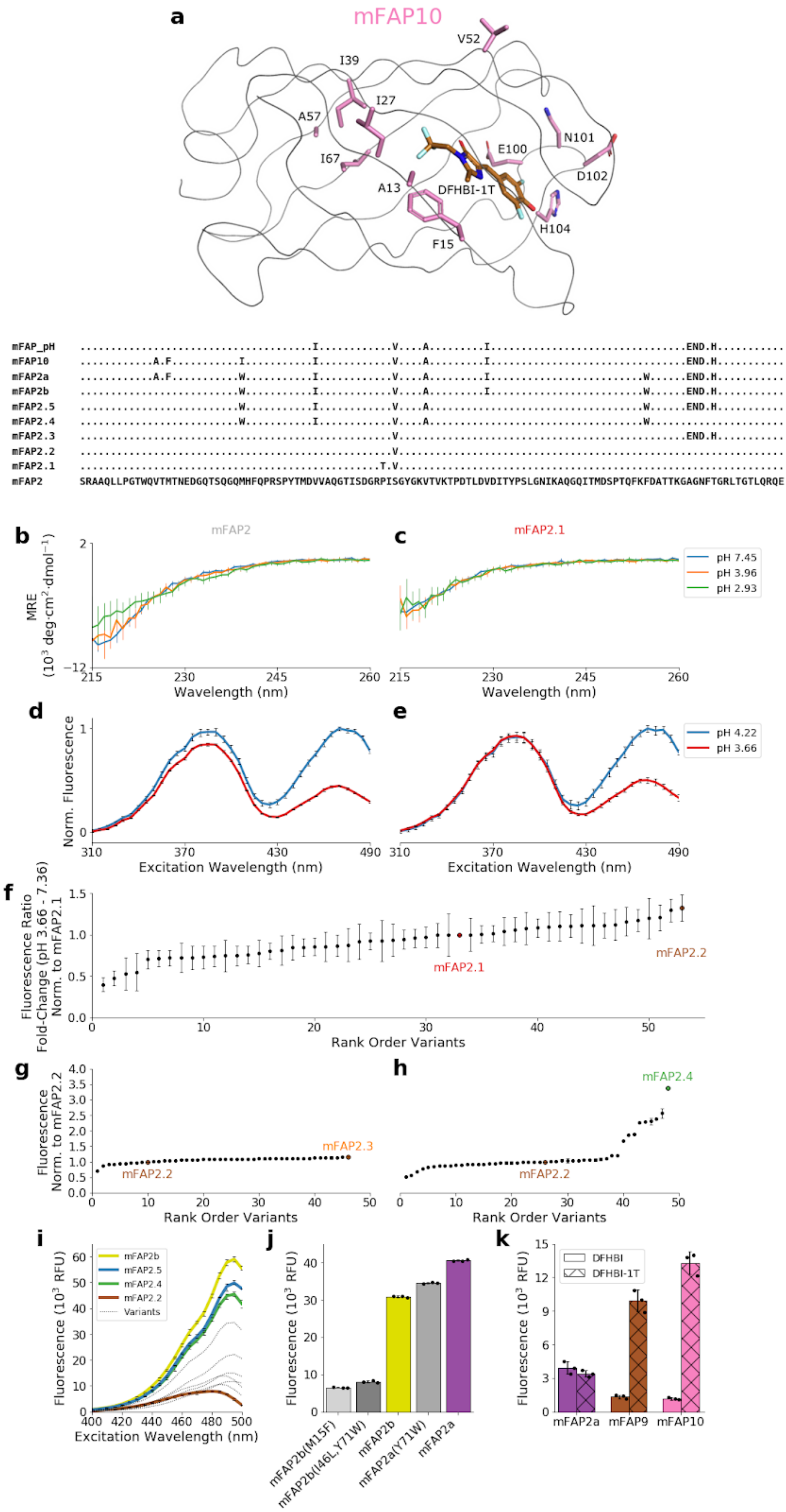
a
mFAP10
V52
I39
I27
A57
I67
E100
N101
D102
A13
DFHBI-1T
H104
F15
mFAP_pH ......................................I............V....A.........I................................END.H............
mFAP10 ............A.F...........I...........I............V....A.........I................................END.H............
mFAP2a ............A.F...........W...........I............V....A.........I..........................W.....END.H............
mFAP2b ..........................W...........I............V....A.........I..........................W.....END.H............
mFAP2.5 ..........................W...........I............V....A....................................W.....END.H............
mFAP2.4 ..........................W...........I............V....A....................................W......................
mFAP2.3 ....V...END.H............
mFAP2.2 ....V..
mFAP2.1 ....T.V..
mFAP2 SRAAQLLPGTWQVTMTNEDGQTSQGQMHFQPRSPYTMDVVAQGTISDGRPISGYGKVTVKTPDTLDVDITYPSLGNIKAQGGQITMDSPTQFKFDATTKGAGNFTGRLTGTLQRQE
b
mFAP2
MRE
(10^3 deg·cm^2·dmol^{-1})
Wavelength (nm)
c
mFAP2.1
pH 7.45
pH 3.96
pH 2.93
Wavelength (nm)
d
e
Norm. Fluorescence
Excitation Wavelength (nm)
pH 4.22
pH 3.66
f
Fluorescence Ratio
Fold-Change (pH 3.66 - 7.36)
Norm. to mFAP2.1
mFAP2.1
mFAP2.2
Rank Order Variants
g
Fluorescence
Norm. to mFAP2.2
mFAP2.3
mFAP2.2
Rank Order Variants
h
mFAP2.4
mFAP2.2
Rank Order Variants
i
mFAP2b
mFAP2.5
mFAP2.4
mFAP2.2
Variants
Fluorescence (10^3 RFU)
Excitation Wavelength (nm)
j
Fluorescence (10^3 RFU)
mFAP2b(M15F)
mFAP2b(I46L,Y71W)
mFAP2b
mFAP2a(Y71W)
mFAP2a
k
DFHBI
DFHBI-1T
Fluorescence (10^3 RFU)
mFAP2a
mFAP9
mFAP10

Supplementary Figure 1.

Engineering of mFAP10, mFAP2a and mFAP2b from mFAP2.

a, Computational model of the mFAP10 protein backbone *(grey ribbon)* and residues mutated from the original mFAP2 design *(pink sticks)* with DFHBI-1T bound *(copper sticks)*. Below, a multiple sequence alignment of mFAP variants presented in this study. **b**, Average (n=1) far-ultraviolet (UV) circular dichroism (CD) spectra of mFAP2 reveals that β-sheet character diminishes at low pH. **c**, Average (n=1) far-UV CD spectra of mFAP2.1 demonstrates similar β-sheet character across high and low pH. **d**, Average (n=3) mFAP2 fluorescence emission spectra reveals that fluorescence emission from the protonated (phenolic) form of DFHBI (with blueshifted excitation peak) diminishes between pH 4.22 and pH 3.66. **e**, Average (n=3) mFAP2.1 fluorescence emission spectra reveals that fluorescence emission from the protonated (phenolic) form of DFHBI (with blueshifted excitation peak) remains approximately constant at pH 4.22 and pH 3.66. **f**, Average (n=3 technical replicates of: n=1 biological replicate for mFAP variants at ranks 1, 2, 3, 4, 6, 7, 8, 9, 10, 12, 13, 17, 18, 21, 23, 24, 25, 26, 27, 32, 34, 35, 36, 37, 40, 42, 50, 52, and 53; n=2 biological replicates for mFAP variants at ranks 5, 11, 14, 15, 16, 30, 31, 38, 39, 41, 44, 45, 47, and 51; n=3 biological replicates for mFAP variants at ranks 20, 28, 46, and 48; n=4 biological replicates for mFAP variants at ranks 22, 33, and 43; and n=5 biological replicates for mFAP variants at ranks 19, 29, and 49) fluorescence ratio fold-change from pH 3.66-7.36 normalized to that of mFAP2.1 of selected mFAP2.1 variants from the β-barrel site-directed mutagenesis (SDM) library (Supplementary Data 3). **g**, Fluorescence intensity (n=1 biological replicate for mFAP variants at ranks 1, 2, 3, 5, 6, 7, 8, 9, 11, 13, 18, 19, 21, 23, 25, 27, 28, 30, 32, 33, 34, 35, 38, 39, 40, 44, and 45) or average fluorescence intensity

(n=2 biological replicates for mFAP variants at ranks 4, 12, 14, 15, 16, 17, 20, 26, 37, and 42; n=3 biological replicates for mFAP variants at ranks 22, 24, 31, 36, and 43; n=4 biological replicates for mFAP variants at ranks 29 and 46; n=6 biological replicates for the mFAP variant at rank 41; and n=20 biological replicates for mFAP2.2) from the deprotonated (phenolate) form of DFHBI normalized to that of mFAP2.2 of selected mFAP2.2 variants from the β-barrel loop7 combinatorial library (Supplementary Data 4). **h**, Fluorescence intensity (n=1 biological replicate for mFAP variants at ranks 2, 3, 5, 6, 7, 10, 11, 12, 13, 14, 15, 16, 17, 18, 21, 23, 24, 25, 27, 31, 33, 34, 38, 39, 40, 41, 43, and 48) or average fluorescence intensity (n=2 biological replicates for mFAP variants at ranks 1, 4, 8, 9, 22, 28, 32, 37, 42, 44, and 46; n=3 biological replicates for mFAP variants at ranks 29, 30, 45, and 47; n=4 biological replicates for mFAP variants at ranks 19 and 36; n=5 biological replicates for mFAP variants at ranks 20 and 35; and n=16 biological replicates for mFAP2.2) from the deprotonated (phenolate) form of DFHBI normalized to that of mFAP2.2 of selected mFAP2.2 variants from the β-barrel core residue combinatorial library (Supplementary Data 5). **i**, Average (n=3) fluorescence excitation spectra at neutral pH of equimolar selected mFAP2.5 variants from the β-barrel methyl group combinatorial library (Supplementary Data 6) labeled with the DFHBI concentration 10-fold below protein concentration. **j**, Average (n=3) fluorescence intensity from the deprotonated (phenolate) form of DFHBI labeled 10-fold below protein concentration of equimolar selected mFAP2b variants from the β-barrel aromatics and aliphatics combinatorial library (Supplementary Data 7). **k**, Average (n=3) fluorescence intensity from the deprotonated (phenolate) forms of DFHBI *(bars)* and DFHBI-1T *(hatched bars)* labeled 680-fold below protein concentration of equimolar mFAP2a, mFAP9 and mFAP10 from the β-barrel "IN2" combinatorial library (Supplementary

Data 9). **b**,**c**, Error bars represent s.d. of the mean of 3 measurements per wavelength. **d**,**e**,**i**,**j**,**k**, Error bars represent s.d. of the mean of 3 technical replicates. **f**, Error bars represent s.d. of the mean of 3 technical replicates of 1-5 biological replicates. **g**,**h**, Error bars represent s.d. of the mean of biological replicates.

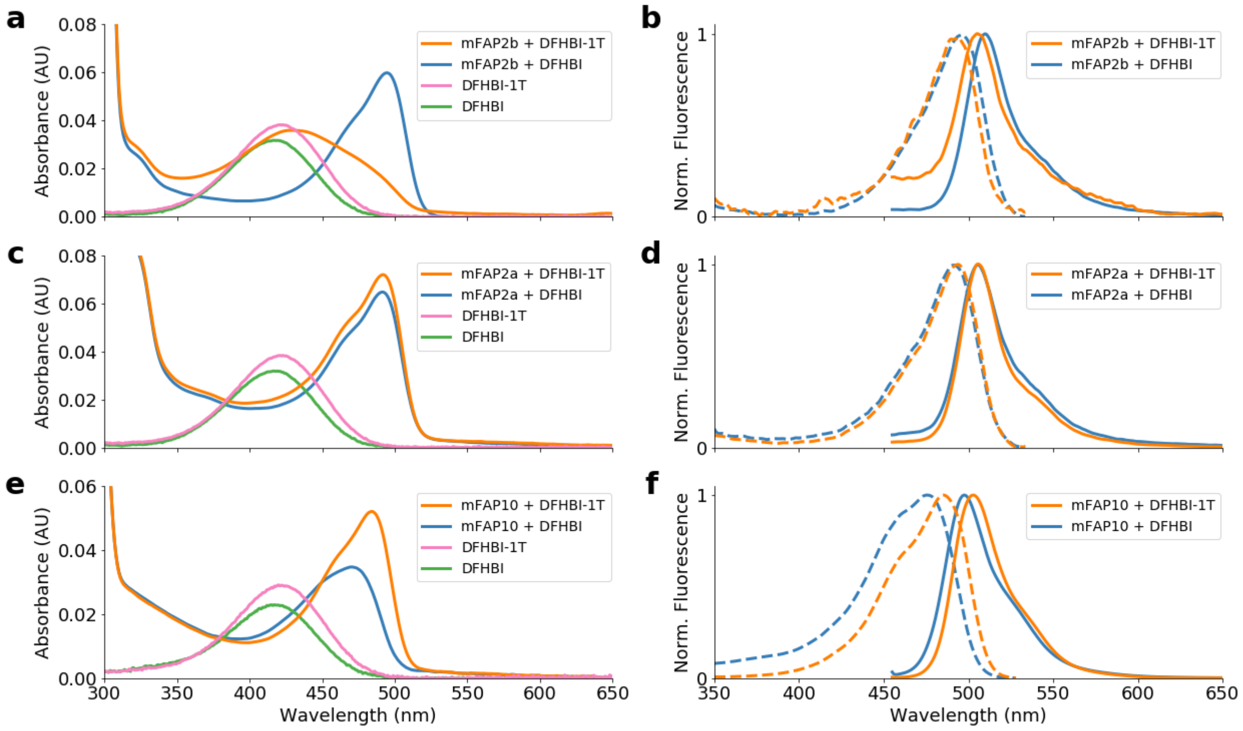

Supplementary Figure 2.

Photophysical characterization of mFAP10, mFAP2a and mFAP2b in complex with DFHBI or DFHBI-1T chromophores, and chromophores only.

a,**c**,**e**, Absorbance spectra (n=1) of saturated protein–chromophore complexes or chromophores only. **b**,**d**,**f**, Normalized fluorescence excitation *(dotted lines)* and emission *(solid lines)* spectra (n=1) of saturated protein–chromophore complexes. **a**,**b**,**c**,**d** The chromophores are at 1.00 µM final concentration. **e**,**f**, The final concentrations of DFHBI are 836 nM and the final concentrations of DFHBI-1T are 919 nM. **a**-**f**, In conditions containing protein and chromophore, the total protein concentration is in excess to the total chromophore concentration, and the percent of the chromophore bound in complex with protein is reported in Table 1.

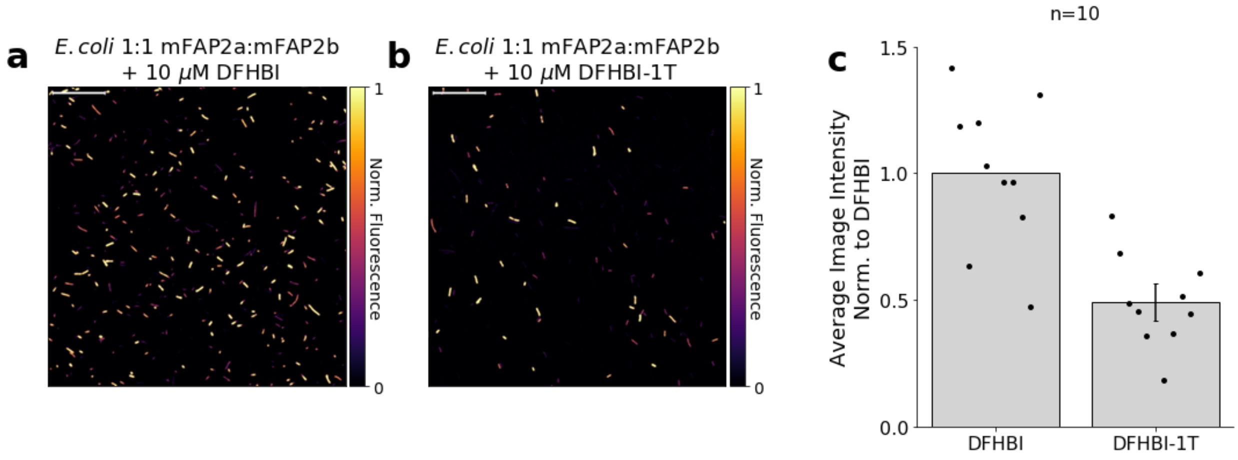

Supplementary Figure 3.

Laser scanning confocal fluorescence microscopy of *E. coli* expressing either mFAP2a or mFAP2b mixed in a 1:1 cellular ratio demonstrating mFAP2b selectivity for DFHBI over DFHBI-1T and mFAP2a promiscuity for DFHBI and DFHBI-1T.

a,**b**, Representative normalized laser scanning confocal fluorescence microscopy image of *E. coli* labeled with (**a**) 10.0 μM DFHBI or (**b**) 10.0 μM DFHBI-1T. Scale bars represent 50 microns. **c**, Average (n=10) of summed pixel intensities from 10 images of *E. coli* mixtures labeled with 10.0 μM DFHBI or 10 images of *E. coli* labeled with 10.0 μM DFHBI-1T normalized to the DFHBI images. Error bars represent the s.e.m. of summed pixel intensities over the 10 images.

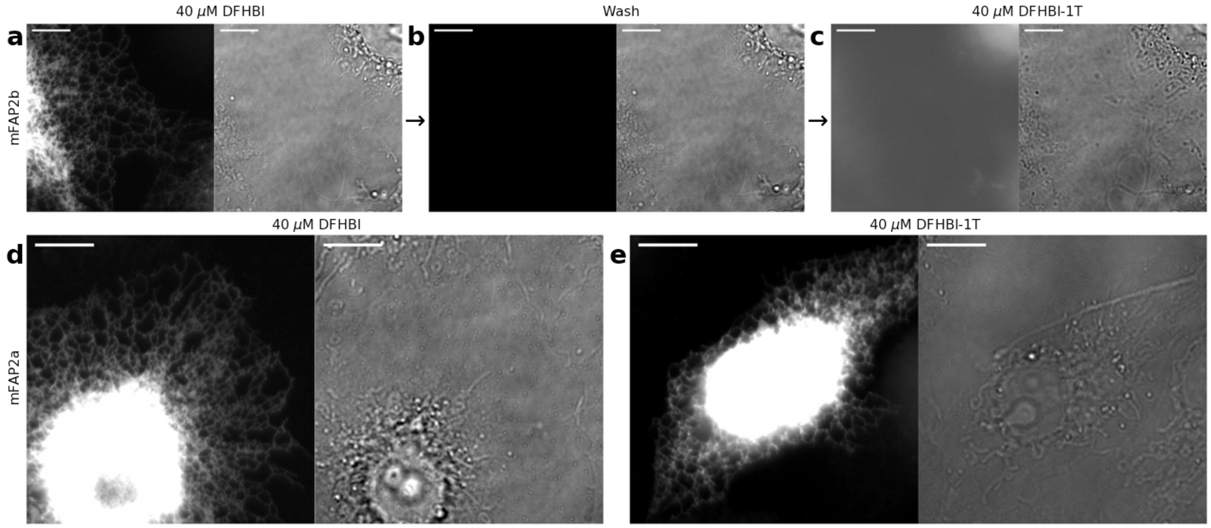

Supplementary Figure 4.

Widefield epifluorescence microscopy of fixed COS-7 cells with expressed mFAP2a or mFAP2b to the endoplasmic reticulum (ER) using a C-terminal sec61β localization sequence.

a-**c**, Images of the same region of an mFAP2b-expressing cell (**a**) labeled with 40.0 μM DFHBI, (**b**) rinsed three times with 1 mL of phosphate buffered saline (PBS), then (**c**) labeled with 40.0 μM DFHBI-1T for 10 minutes, demonstrating reversible on-demand fluorescence. **d**,**e**, Images of different mFAP2a-expressing cells labeled with either (**d**) 40.0 μM DFHBI or (**e**) 40.0 μM DFHBI-1T. **a**-**e**, Panels show fluorescence images *(left)* and brightfield images *(right)*. Scale bars represent 10 microns. Experiments were performed once. Exposure times were 200 ms and current was 500 mA.

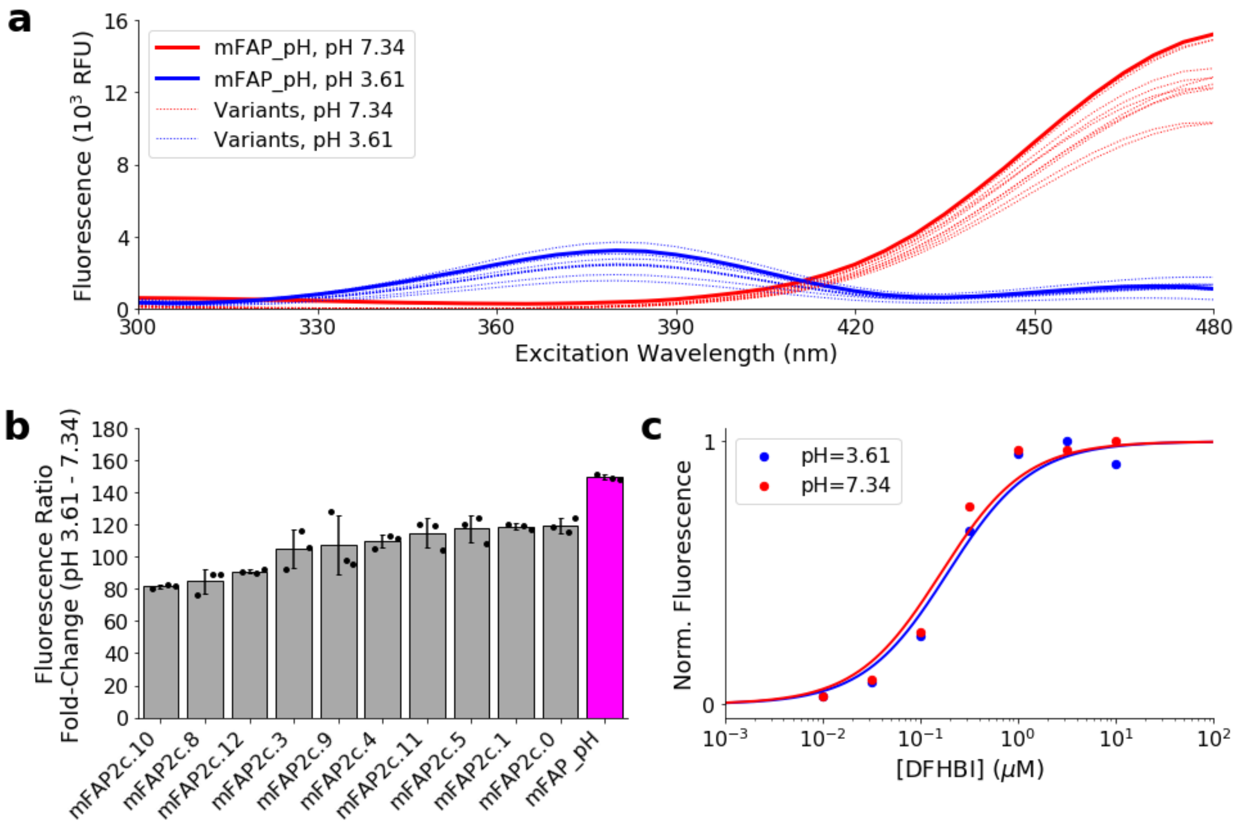

Supplementary Figure 5.

Photophysical characterization of pH-responsive mFAP variants.

a, Fluorescence excitation spectra of selected pH-responsive mFAP variants. **b**, Average (n=3) fluorescence ratio fold-change from pH 3.61-7.34 of selected pH-responsive mFAP variants. Error bars represent s.d. of the mean of 3 technical replicates. **c**, *In vitro* titration of DFHBI with mFAP_pH monitoring fluorescence emission of the protonated (phenolic) form of DFHBI from the blueshifted excitation peak at pH 3.61 *(blue points)*, and fluorescence emission of the deprotonated (phenolate) form of DFHBI from the redshifted excitation peak at pH 7.34 *(red points)*. Normalized fluorescence intensities (n=1) were fit to a single binding site isotherm function *(lines)* using non-linear least squares fitting to obtain K_d values.

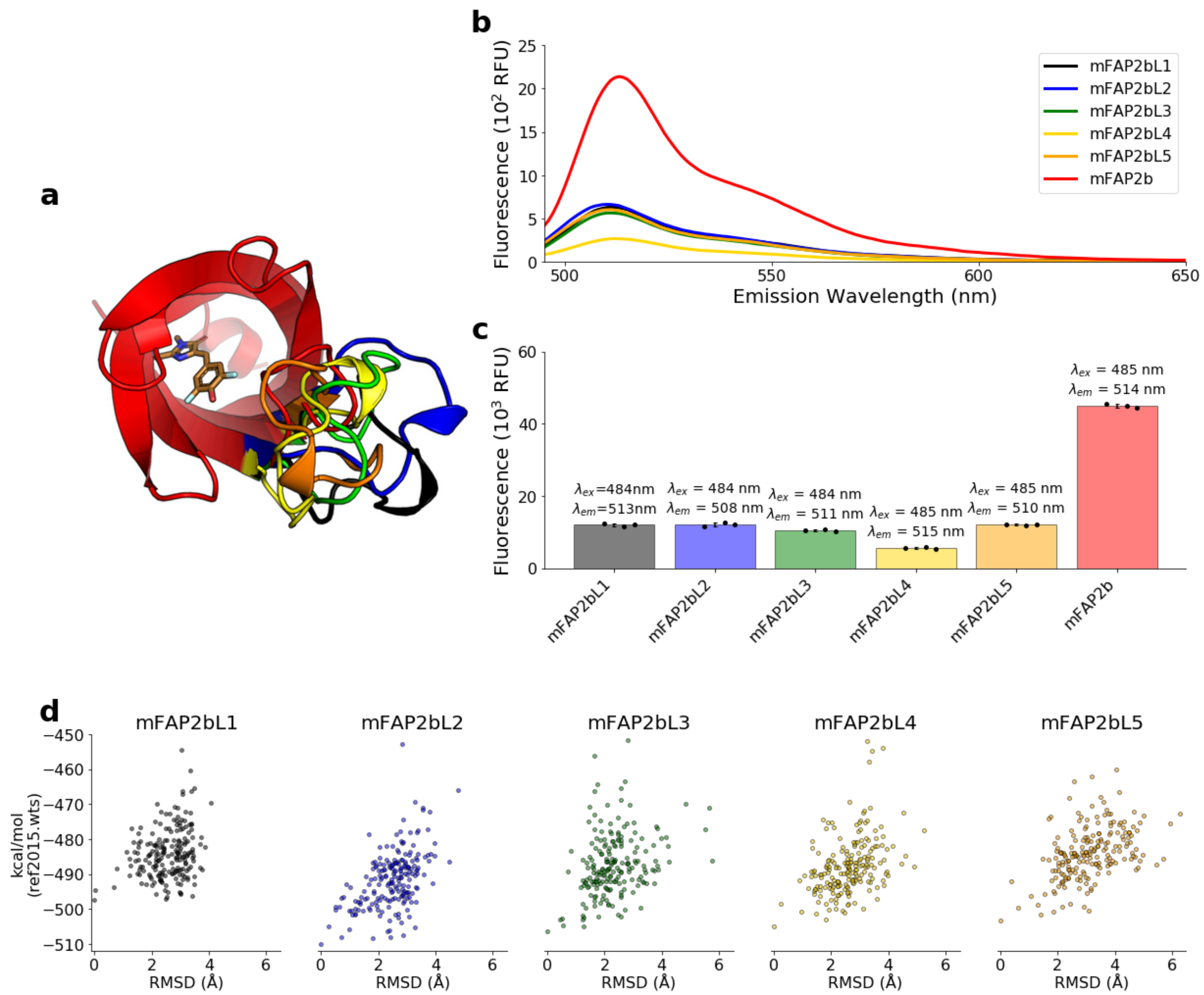

Supplementary Figure 6.

Photophysical characterization and computational modeling of extended loop7 variants.

a, Aligned computational models of mFAP2b *(red cartoon)* and five extended loop7 variants *(colored according to* **b**,**c**) including mFAP2bL1 *(black cartoon)*, mFAP2bL2 *(blue cartoon)*, mFAP2bL3 *(green cartoon)*, mFAP2bL4 *(yellow cartoon)*, mFAP2bL5 *(orange cartoon)* with DFHBI bound *(copper sticks)*. **b**, Fluorescence emission spectra from the deprotonated (phenolate) form of DFHBI labeled 10-fold below protein concentration for equimolar mFAP2b *(red)* and five extended loop7 variants *(colored according to* **a**,**c**). **c**, Average (n=3) fluorescence

intensity from the deprotonated (phenolate) form of DFHBI labeled 10-fold below protein concentration at the indicated peak excitation and emission wavelengths for equimolar mFAP2b and five extended loop7 variants *(colored according to* **a**,**b***)*. Error bars represent s.d. of the mean of 3 technical replicates. **d**, Modeled loop7 residues 95-112 of the five fluorescent extended loop7 variants were computationally refined using the Rosetta loopmodel application[1,2,3,4] over 200 repeats with the "ref2015.wts" energy function[5] (Supplementary Note 2). For each computational model, the root mean square deviation (RMSD) of protein backbone heavy atoms to the lowest energy ($kcal \cdot mol^{-1}$) computational model (shown in **a**) was computed after protein backbone C_α-C_α atom alignment.

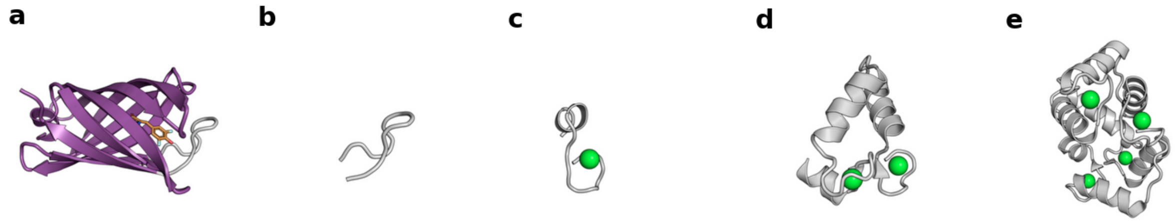

Supplementary Figure 7.

Structures of motifs conferring Ca^{2+}-responsiveness to mFAPs.

a, Computational model of *de novo* designed β-barrel scaffold *(purple cartoon)* with bound DFHBI *(copper sticks)* showing replaceable loop7 *(grey cartoon)*. **b**, Computational model of mFAP2b loop7. **c**, Solution nuclear magnetic resonance (NMR) structure of Ca^{2+}-binding peptide *(grey cartoon)* bound to one La^{3+} ion *(green sphere)* from RCSB Protein Data Bank (PDB) accession code 1NKF[6]. **d**, X-ray crystal structure of *E. coli* fragment TR2C from the Ca^{2+}-binding protein calmodulin *(grey cartoon)* bound to two Ca^{2+} ions *(green spheres)* from PDB accession code 1FW4[7]. **e**, Crystal structure of bovine brain calmodulin in a compact form *(grey cartoon)* bound to four Ca^{2+} ions *(green spheres)* from PDB accession code 1PRW[8]. (**a**-**e**) Models and structures are shown at different scales.

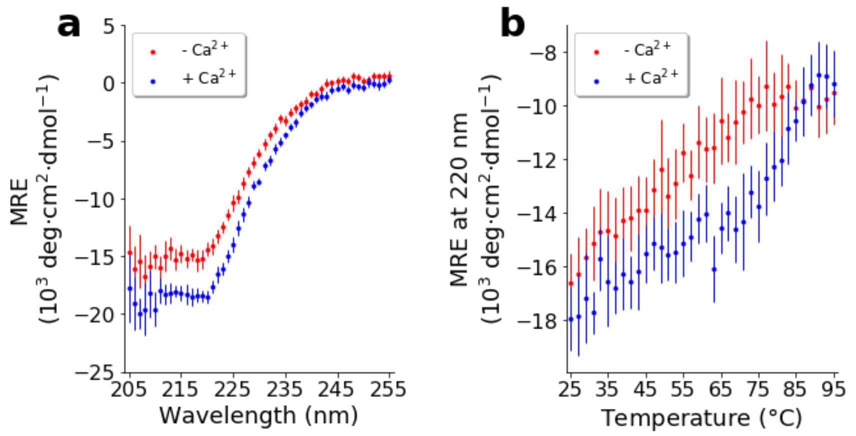

Supplementary Figure 8.

Far-ultraviolet (UV) circular dichroism (CD) characterization of EF4n_mFAP2b without DFHBI.

a, Average (n=1) far-UV CD wavelength scans in either buffer only *(red)* or buffer supplemented with 100 µM $CaCl_2$ *(blue)*. **b**, Thermal denaturation monitoring average (n=1) CD signal at 220 nm in either buffer only *(red)* or buffer supplemented with 100 µM $CaCl_2$ *(blue)*. **a**,**b**, Reported averages are background subtracted using averages (n=1) at identical conditions in the absence of protein. Error bars represent s.d. of the mean of 3 independent measurements (**a**) per wavelength or (**b**) per °C.

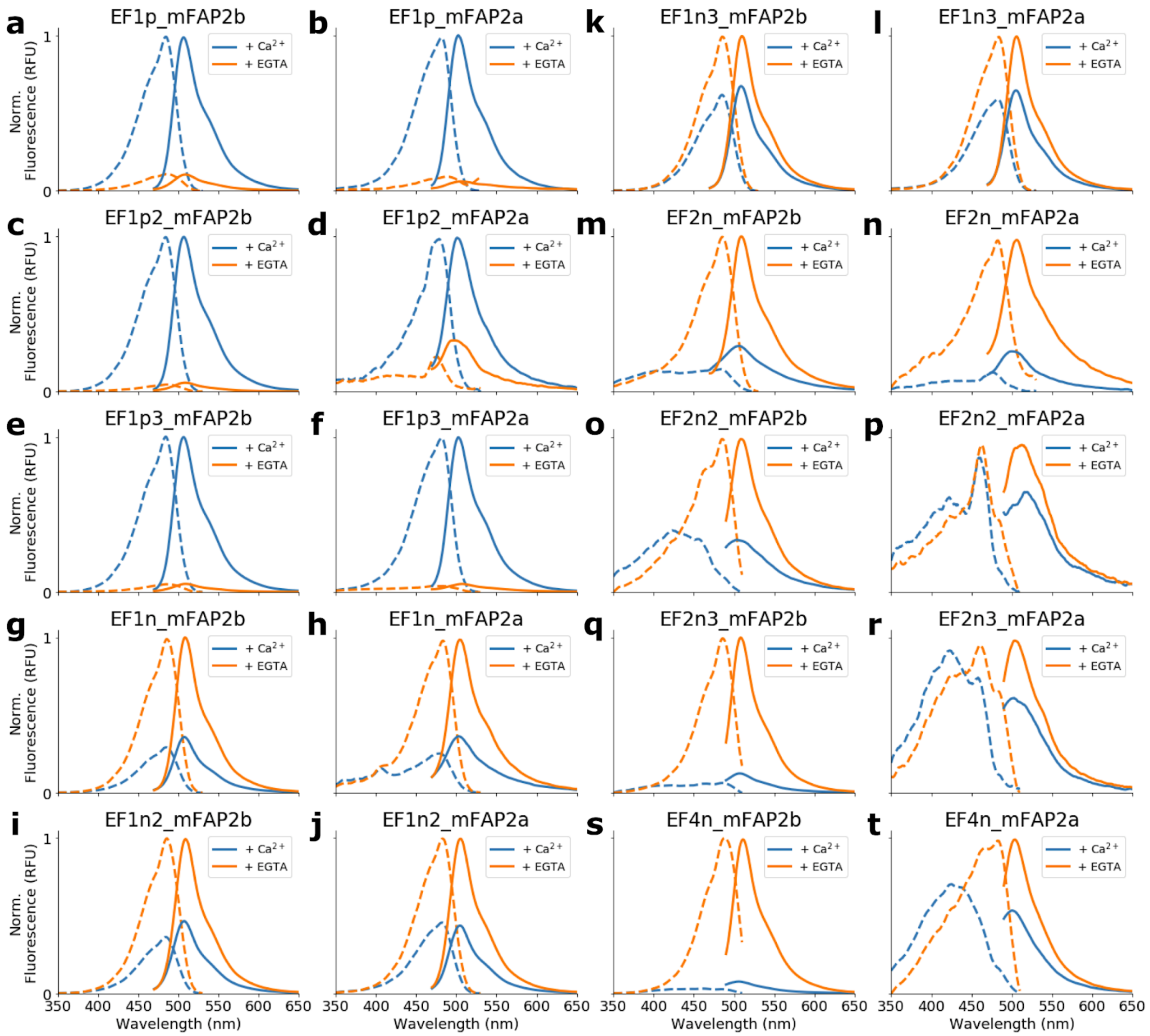

Supplementary Figure 9.

Photophysical characterization of Ca^{2+}-responsive mFAPs.

a-t, Normalized fluorescence excitation *(dotted lines)* and emission *(solid lines)* spectra (n=1) of Ca^{2+}-responsive mFAPs in either 750 mM $CaCl_2$ *(blue)* or 1.50 mM EGTA *(orange)*. **a-f**, Positively allosteric Ca^{2+}-responsive mFAPs are labeled with DFHBI concentrations at their $\frac{K_d^{+}}{2}$, respectively (Supplementary Table 2). **g-t**, Negatively allosteric Ca^{2+}-responsive mFAPs are

labeled with DFHBI concentrations at their $\frac{\bar{K_d}}{2}$, respectively (Supplementary Table 2). The fluorescence dynamic ranges (F_{max}/F_{min}) using the fluorescence emission intensities at peak emission wavelengths are reported in Supplementary Table 2.

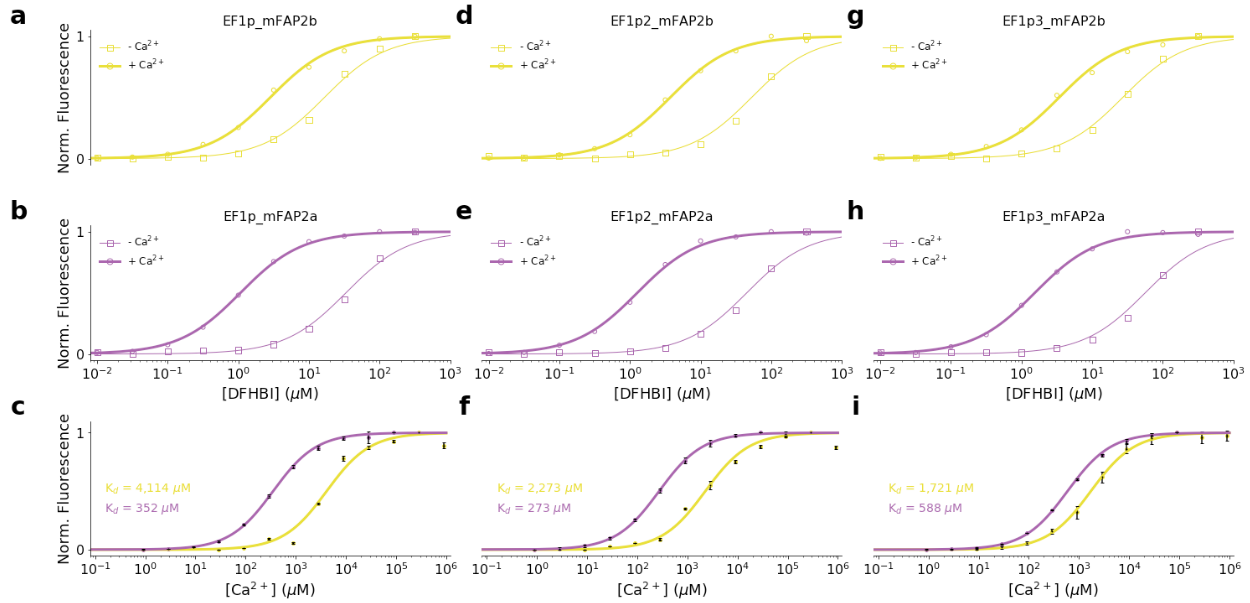

Supplementary Figure 10.

Normalized fluorescence of positively allosteric Ca^{2+}-responsive mFAPs containing one EF-hand motif.

a,**b**,**d**,**e**,**g**,**h**, DFHBI titrations of 500 nM sensors in buffer *(squares)* or buffer supplemented with 450 mM $CaCl_2$ *(circles)*. **c**,**f**,**i**, Ca^{2+} titrations of 500 nM sensors with 5.00 μM DFHBI. Error bars represent s.d. of the mean of 3 technical replicates. **a-i**, Non-linear least squares fitting to a single binding site isotherm function with Hill coefficients of 1 were used to obtain K_d^- (**a**,**b**,**d**,**e**,**g**,**h**, *thin lines*), K_d^+ (**a**,**b**,**d**,**e**,**g**,**h**, *thick lines*), and Ca^{2+} K_d (**c**,**f**,**i**, *thick lines)* values (Supplementary Table 2).

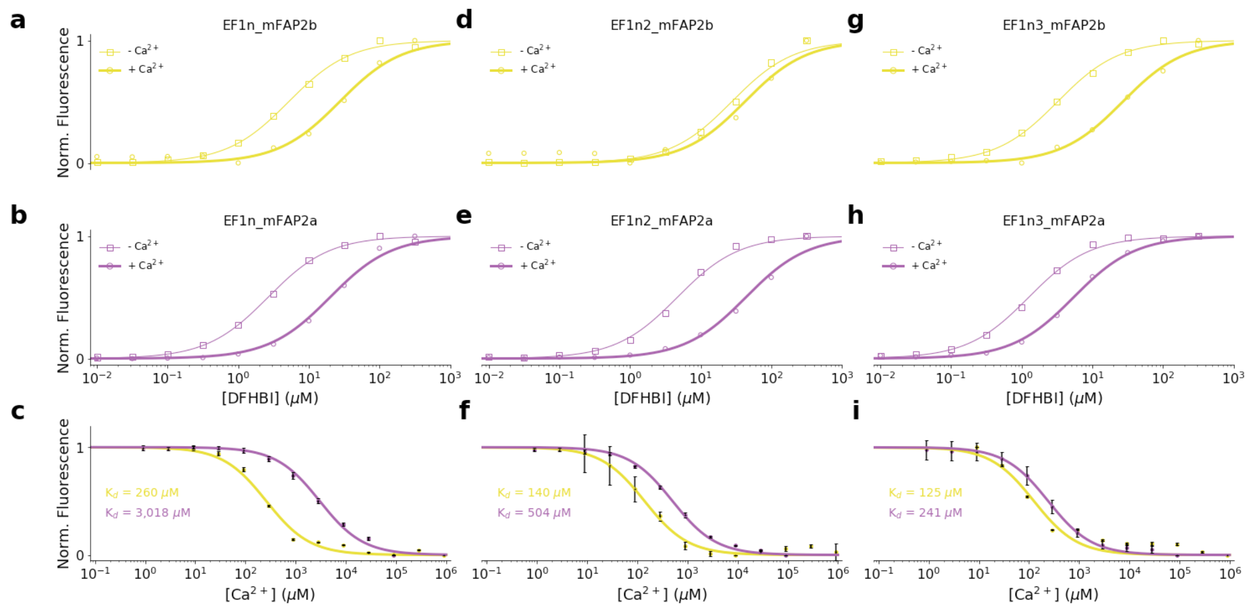

Supplementary Figure 11.

Normalized fluorescence of negatively allosteric Ca^{2+}-responsive mFAPs containing one EF-hand motif.

a,**b**,**d**,**e**,**g**,**h**, DFHBI titrations of 500 nM sensors in buffer *(squares)* or buffer supplemented with 450 mM $CaCl_2$ *(circles)*. **c**,**f**,**i**, Ca^{2+} titrations of 500 nM sensors with 5.00 µM DFHBI. Error bars represent s.d. of the mean of 3 technical replicates. **a-i**, Non-linear least squares fitting to (**a**,**b**,**d**,**e**,**g**,**h**) a single binding site isotherm function or (**c**,**f**,**i**) an inverse single binding site isotherm function with Hill coefficients of 1 were used to obtain K_d^- (**a**,**b**,**d**,**e**,**g**,**h**, *thin lines*), K_d^+ (**a**,**b**,**d**,**e**,**g**,**h**, *thick lines*), and Ca^{2+} K_d (**c**,**f**,**i**, *thick lines)* values (Supplementary Table 2).

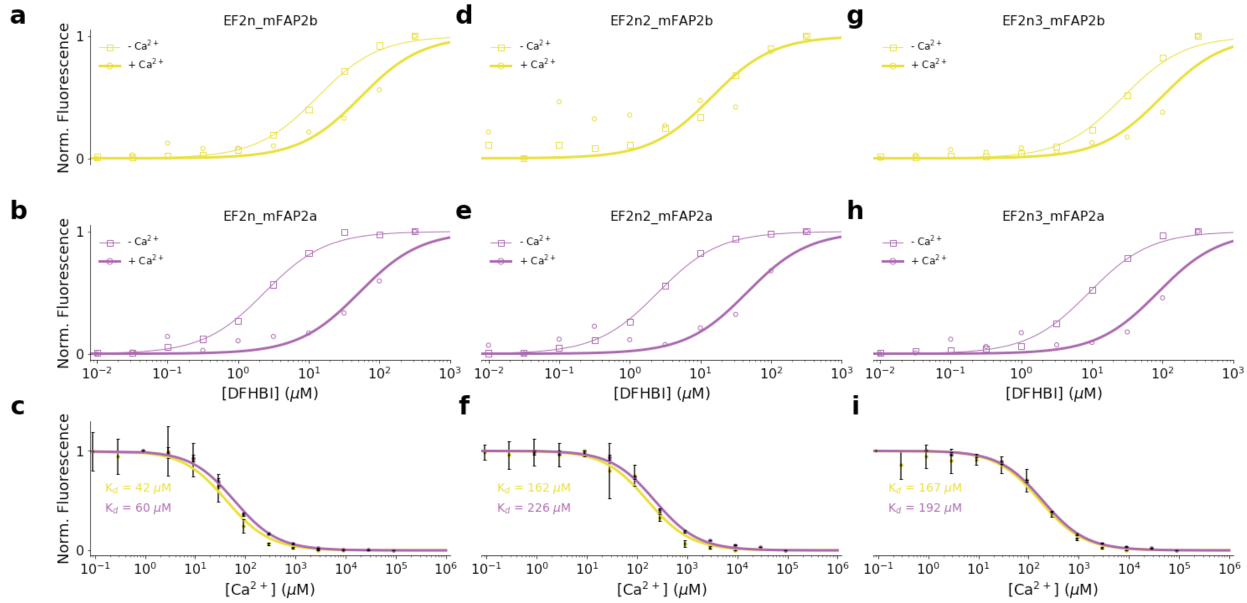

Supplementary Figure 12.

Normalized fluorescence of negatively allosteric Ca^{2+}-responsive mFAPs containing two EF-hand motifs.

a,**b**,**d**,**e**,**g**,**h**, DFHBI titrations of 500 nM sensors in buffer *(squares)* or buffer supplemented with 450 mM $CaCl_2$ *(circles)*. **c**,**f**,**i**, Ca^{2+} titrations of 500 nM sensors with 5.00 μM DFHBI. Error bars represent s.d. of the mean of 3 technical replicates. **a**-**i**, Non-linear least squares fitting to (**a**,**b**,**d**,**e**,**g**,**h**) a single binding site isotherm function or (**c**,**f**,**i**) an inverse single binding site isotherm function with Hill coefficients of 1 were used to obtain K_d^- (**a**,**b**,**d**,**e**,**g**,**h**, *thin lines*), K_d^+ (**a**,**b**,**d**,**e**,**g**,**h**, *thick lines*), and Ca^{2+} K_d (**c**,**f**,**i**, *thick lines)* values (Supplementary Table 2).

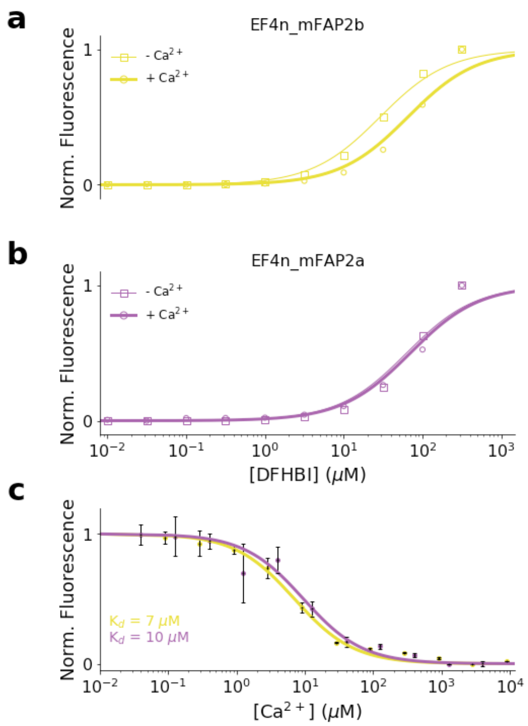

Supplementary Figure 13.

Normalized fluorescence of negatively allosteric Ca^{2+}-responsive mFAPs containing four EF-hand motifs.

a,**b**, DFHBI titrations of 5.00 µM sensors in either 500 µM EGTA *(squares)* or 500 µM $CaCl_2$ *(circles)*. **c**, Ca^{2+} titrations of 6.25 µM EF4n_mFAP2b with 43.4 µM DFHBI *(lime),* and 8.00 µM EF4n_mFAP2a with 80.0 µM DFHBI *(violet),* using buffer pre-treated with Chelex 100. Error bars represent s.d. of the mean of 3 technical replicates. **a-c**, Non-linear least squares fitting to (**a**,**b**) a single binding site isotherm function or (**c**) an inverse single binding site isotherm function with Hill coefficients of 1 were used to obtain K_d^- (**a**,**b**,**d**,**e**,**g**,**h**, *thin lines*), K_d^+ (**a**,**b**,**d**,**e**,**g**,**h**, *thick lines*), and Ca^{2+} K_d (**c**,**f**,**i**, *thick lines)* values (Supplementary Table 2).

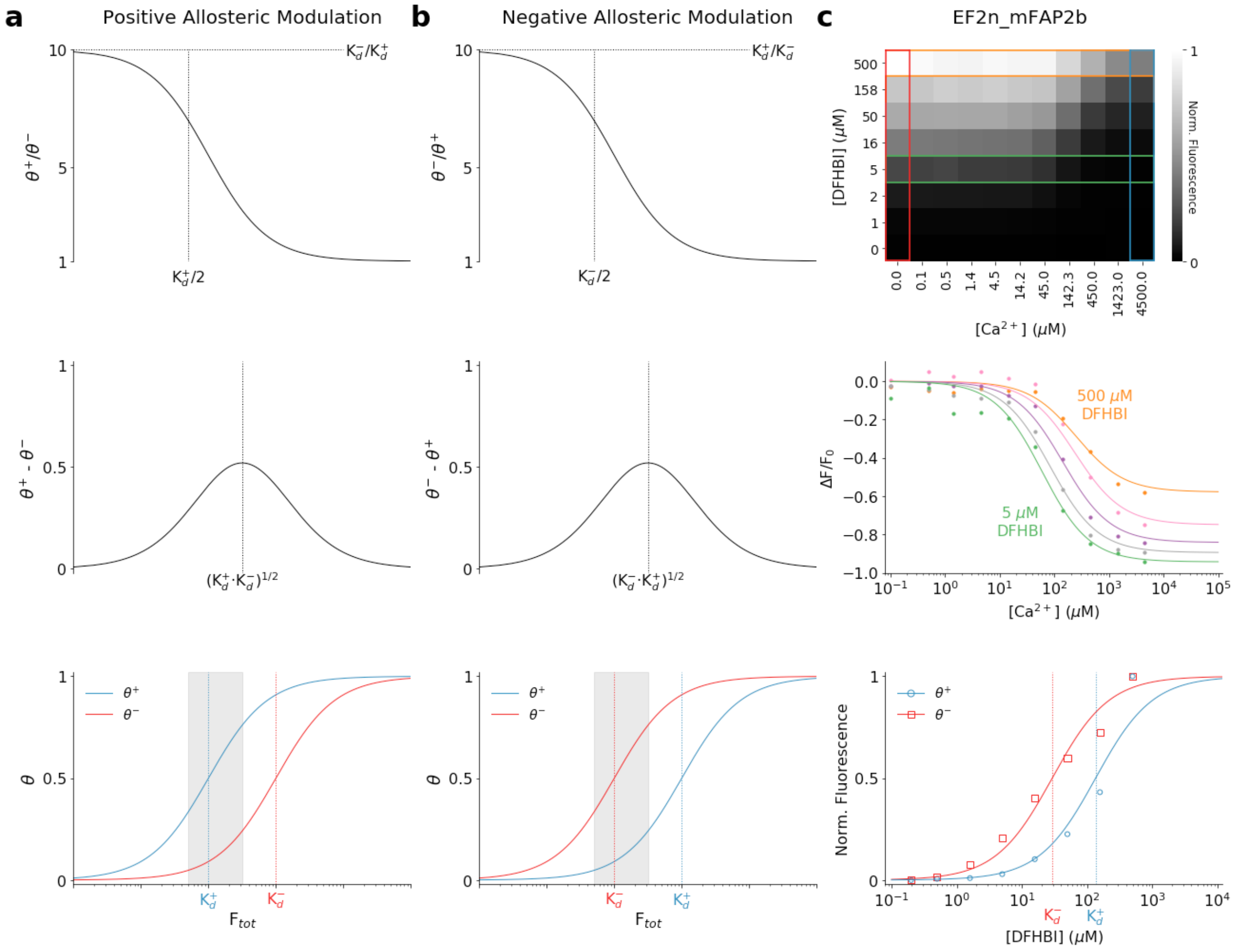

Supplementary Figure 14.

Thermodynamic properties of Ca^{2+}-responsive mFAPs.

a,**b**, Theoretical fraction of protein–fluorogen complex to total protein in excess Ca^{2+}, θ^+ *(blue)*, and in absence of Ca^{2+}, θ^- *(red)*, simulated with a 10-fold difference in the fluorogen thermodynamic dissociation constant between in excess Ca^{2+} (K_d^+) and in absence of Ca^{2+} (K_d^-) for (**a**) positive allosteric modulation and (**b**) negative allosteric modulation. *(Top row)* Theoretical dependence of the fractional change in the ratio of protein–fluorogen complex to total protein upon Ca^{2+} binding (i.e. the theoretical fluorescence dynamic range of the sensor), (**a**) $\frac{\theta^+}{\theta^-}$ for positive allosteric modulation or (**b**) $\frac{\theta^-}{\theta^+}$ for negative allosteric modulation, on the total

fluorogen concentration, F_{tot}. *(Middle row)* Theoretical dependence of the difference in the ratio of protein–fluorogen complex to total protein upon Ca^{2+} binding on the total fluorogen concentration, F_{tot}. *(Bottom row)* Choosing F_{tot} between the range $\frac{K_d^+}{2} \leq F_{tot} \leq (K_d^+ \cdot K_d^-)^{1/2}$ for positive allosteric modulation (**a**, *grey rectangle*) or between the range $\frac{K_d^-}{2} \leq F_{tot} \leq (K_d^- \cdot K_d^+)^{1/2}$ for negative allosteric modulation (**b**, *grey rectangle*) provides a compromise in both good dynamic range and satisfactory detection sensitivity of the sensor (see Supplementary Note 1). **c**, Experimental DFHBI titration versus Ca^{2+} titration of a negatively allosteric Ca^{2+}-responsive mFAP, EF2n_mFAP2b. The heatmap *(top row)* represents the fluorescence intensities normalized from 0 to 1 across all DFHBI and Ca^{2+} concentrations. Normalized fluorescence intensities from the heatmap in the absence of Ca^{2+} *(red box)* and in excess Ca^{2+} *(blue box)* are fit to a single binding site isotherm function using non-linear least squares fitting *(bottom row lines)* to obtain the fluorogen thermodynamic dissociation constant in the absence of Ca^{2+} (K_d^-) and in excess Ca^{2+} (K_d^+). Unnormalized fluorescence intensities from Ca^{2+} titrations at DFHBI concentrations between 5.00 μM *(green box)* and 500 μM *(orange box)* from the heatmap were used to calculate the fluorescence fold-change ($\frac{\Delta F}{F_0}$) at each DFHBI concentration *(middle row)*, which were then fit to an inverse single binding site isotherm function using non-linear least squares fitting and scaled to the minimum values *(middle row lines)*. The dependence of the peak magnitude of fluorescence fold-change ($\frac{\Delta F}{F_0}$) on DFHBI concentration follows the theoretical dependence of $\frac{\theta^-}{\theta^+}$ on F_{tot} for negative allosteric modulation (**b**, *top row*). Due to the thermodynamic coupling between Ca^{2+} and DFHBI binding, it is evident that the thermodynamic dissociation constant for Ca^{2+} follows the DFHBI concentration for negatively allosteric Ca^{2+}-responsive mFAPs.

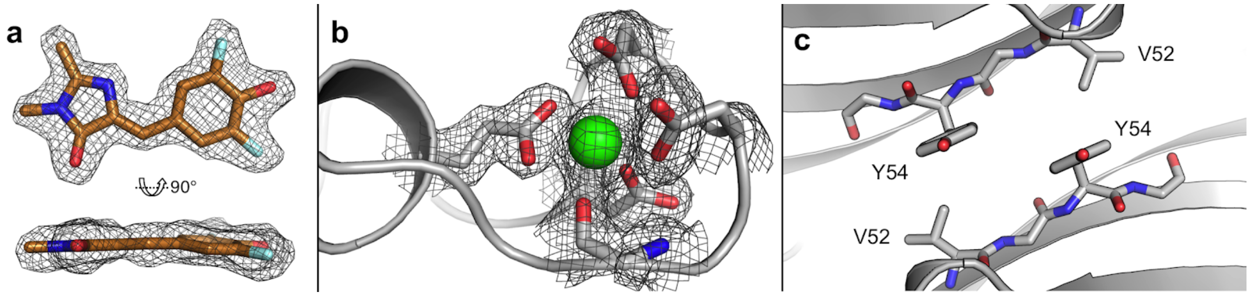

Supplementary Figure 15.

X-ray co-crystal structure of EF1p2_mFAP2b–DFHBI–Ca^{2+} solved at 2.1 Å resolution as a dimeric asymmetric unit (PDB accession code 6OHH).

a, DFHBI *(copper sticks)* shown with 2Fo-Fc omit density maps indicate that DFHBI is in the *cis*-planar conformation in the crystal structure. **b**, Structure of grafted EF-hand motif *(grey cartoon)*, bound Ca^{2+} *(green sphere)*, and Ca^{2+}-coordinating residues *(grey sticks)* shown with 2Fo-Fc omit density map. **c**, Asymmetric unit dimer interface suggests that the S52V mutation (acquired from mFAP2 to mFAP2.1 and incorporated into EF1p2_mFAP2b), and additionally Y54 present on mFAP2, mFAP2.1 and EF1p2_mFAP2b, promotes crystallization by forming hydrophobic contacts between β-barrel monomers.

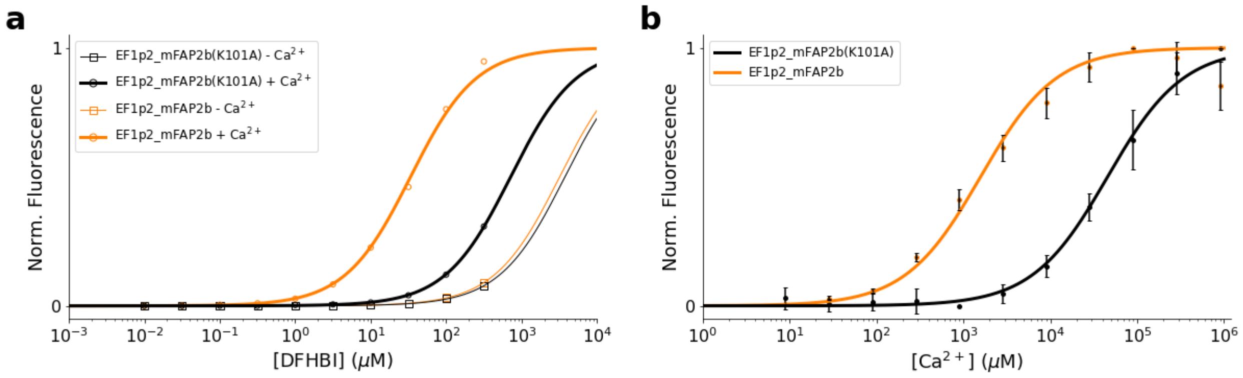

Supplementary Figure 16.

Normalized fluorescence of Ca^{2+}-responsive mFAP EF1p2_mFAP2b with and without the K101A single residue mutation.

a, DFHBI titrations of 500 nM sensors in buffer *(squares)* or buffer supplemented with 450 mM $CaCl_2$ *(circles)*. **b**, Ca^{2+} titrations of 500 nM sensors with 5.00 µM DFHBI. Error bars represent s.d. of the mean of 3 technical replicates. **a**,**b**, Non-linear least squares fitting to a single binding site isotherm function with Hill coefficients of 1 were used to obtain K_d^- (**a**, *thin lines*), K_d^+ (**a**, *thick lines*), and Ca^{2+} K_d (**b**, *thick lines)* values.

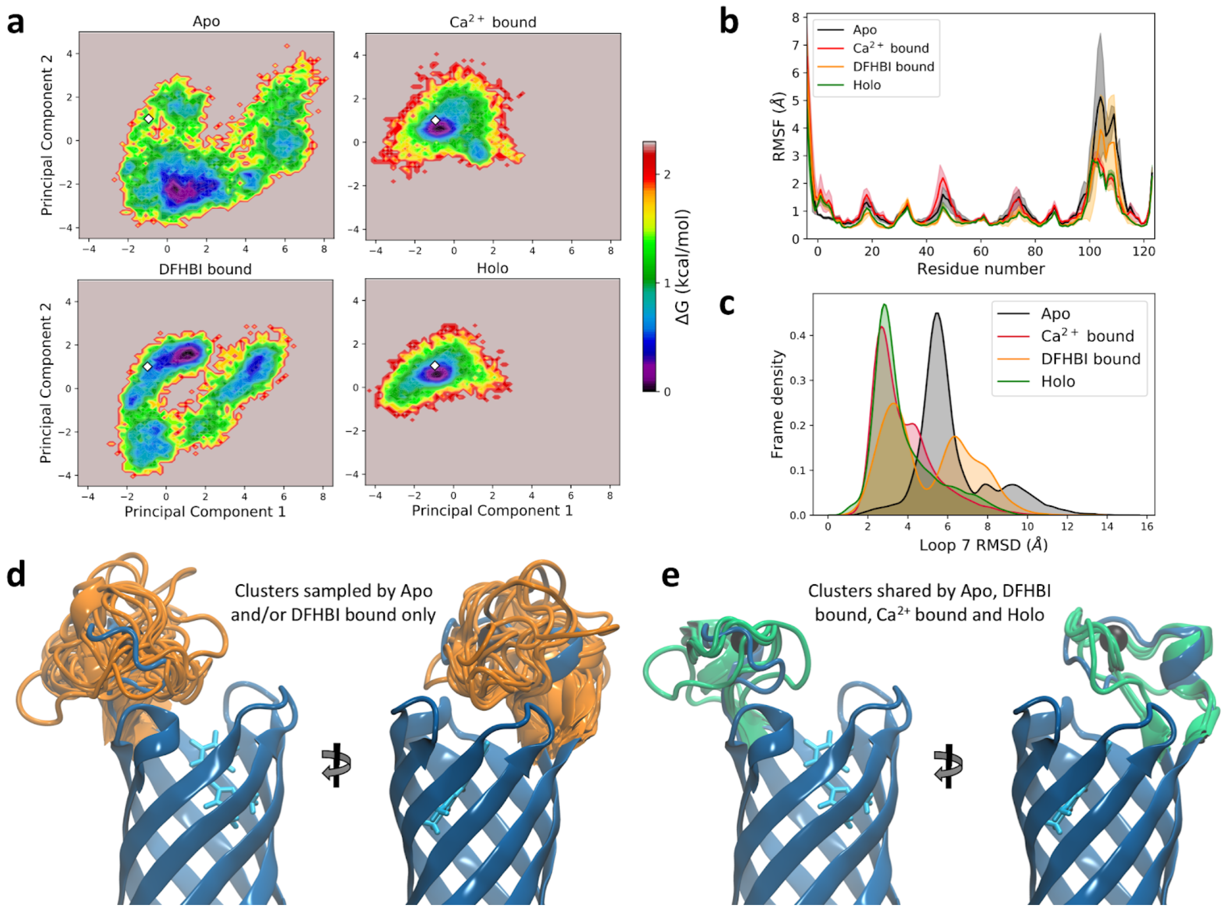

Supplementary Figure 17.

Molecular dynamics (MD) simulations of the X-ray crystal structure of EF1p2_mFAP2b under the four conditions: Apo, Ca^{2+}-bound, DFHBI-bound, and Holo.

a, Conformational free energy landscape in terms of the principal components of the protein backbone atom coordinates. The coordinates of the crystallographic structure are shown *(white diamonds)*. The Ca^{2+}-bound and Holo MD simulations did not diverge significantly from the crystal conformation *(white diamonds)* and displayed similar conformational free energy profiles, while the Apo and DFHBI-bound systems presented a wider and shallower free energy landscape and sampled a large range of conformations with pronounced deviation from the

crystal structure coordinates *(white diamonds)*. **b**, Average root mean square fluctuations (RMSF) of the protein backbone C_α atoms *(lines)* and s.d. of the means *(shaded)* of 3 replicates per condition revealed that the main source of conformational variability stems from the fluctuations of loop7. Apo and DFHBI-bound systems displayed a large degree of loop7 flexibility, whereas Ca^{2+}-bound and Holo showed dampened and similar degrees of fluctuations. **c**, Smoothed histograms of the root mean square deviation (RMSD) of loop7 backbone C, N and C_α atoms in the MD simulations from the crystallographic loop7 conformation. DFHBI-bound simulations, particularly, had a two-lobe RMSD distribution, in agreement with the two low-energy wells seen in the conformational free energy landscape in (**a**). **d**, Centroid structures from clusters sampled exclusively by the Apo and DFHBI-bound MD simulations *(orange cartoons)* overlaid onto the protein crystal structure *(blue cartoon)* with DFHBI *(cyan sticks)* and Ca^{2+} *(black sphere)* bound. The centroid structures point to the high degree of flexibility of loop7 in the absence of Ca^{2+}. **e**, Centroids of the clusters that are sampled by all four conditions. Loop7 conformations *(green cartoons)* are overlaid onto the protein crystal structure *(blue cartoon)* with DFHBI *(cyan sticks)* and Ca^{2+} *(black sphere)* bound. No clusters were sampled exclusively by Ca^{2+} and Holo simulations, since the conformational ensemble of these simulations showed overlap with regions of the Apo and DFHBI-bound free energy landscape in (**a**). Thus, the clusters to which the Ca^{2+} and Holo simulations were assigned contained a minority of frames from the Apo and DFHBI-bound simulations as well. These cluster centroids showed a much more organized loop7, with smaller variations from the crystal structure coordinates. **d**,**e**, The MD simulation conditions which did not contain Ca^{2+} bound in loop7 sampled a larger variety of conformations, which also included the active (fluorescent), Holo-like conformation to a small

degree. The introduction of the Ca^{2+} into loop7, however, favored the active conformation in a conformational selection manner and stabilized loop7 fluctuations.

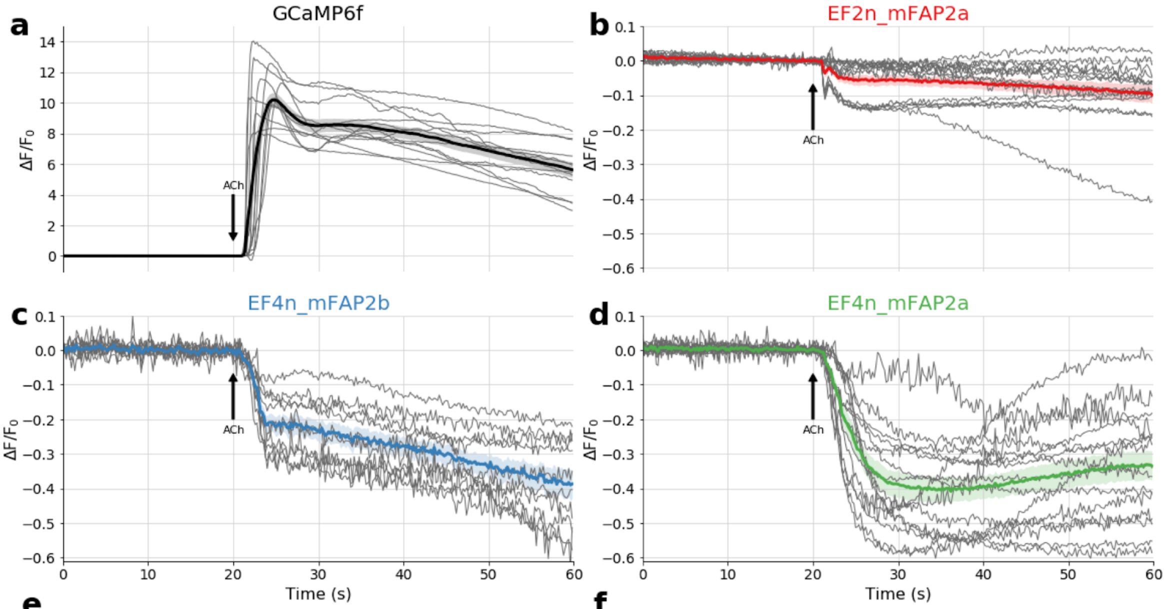

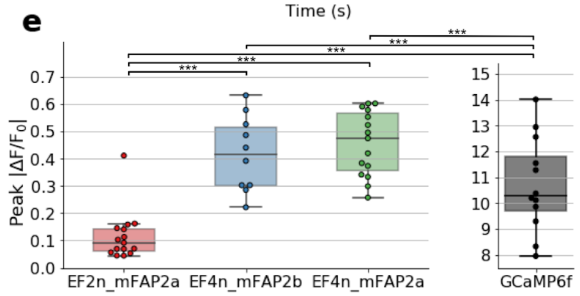

f

Fluorescent Sensors		P-value
EF2n_mFAP2a	EF4n_mFAP2a	$1.1 \cdot 10^{-5}$
EF4n_mFAP2a	EF4n_mFAP2b	$5.2 \cdot 10^{-1}$
EF2n_mFAP2a	EF4n_mFAP2b	$1.2 \cdot 10^{-4}$
EF2n_mFAP2a	GCaMP6f	$1.3 \cdot 10^{-5}$
EF4n_mFAP2a	GCaMP6f	$1.3 \cdot 10^{-5}$
EF4n_mFAP2b	GCaMP6f	$8.7 \cdot 10^{-5}$

Supplementary Figure 18.

Time-lapse epifluorescence microscopy of acetylcholine (ACh) stimulations of live HEK293 cells expressing cytosolic Ca^{2+}-responsive mFAPs or GCaMP6f.

a-**d**, 100 µM ACh was added *(black arrows)* and regions of interest (ROIs) surrounding single cells were analyzed for fluorescence fold-change ($\frac{\Delta F}{F_0}$) for either (**a**) GCaMP6f (n=12 ROIs from 3 technical replicates), (**b**) EF2n_mFAP2a (n=15 ROIs from 3 technical replicates) labeled at 20.0 µM DFHBI, (**c**) EF4n_mFAP2b (n=10 ROIs from 3 technical replicates) labeled at 43.3 µM DFHBI, or (**d**) EF4n_mFAP2a (n=15 ROIs from 3 technical replicates) 43.3 µM DFHBI. The mean $\frac{\Delta F}{F_0}$ *(thick lines)* and s.e.m. *(shading)* are shown for the ROIs *(grey lines)*. **e**, For each ROI in (**a**-**d**), the peak absolute value of the fluorescence fold-change upon ACh stimulation is

plotted showing: center line, median; box limits, upper and lower quartiles; whiskers, distribution excluding outliers; points, peak absolute values of the fluorescence fold-change upon ACh stimulation. The two-sided Wilcoxon rank sum test was used to statistically compare groups (*** denotes P-value < 0.001). **f**, P-values between groups plotted in (**e**).

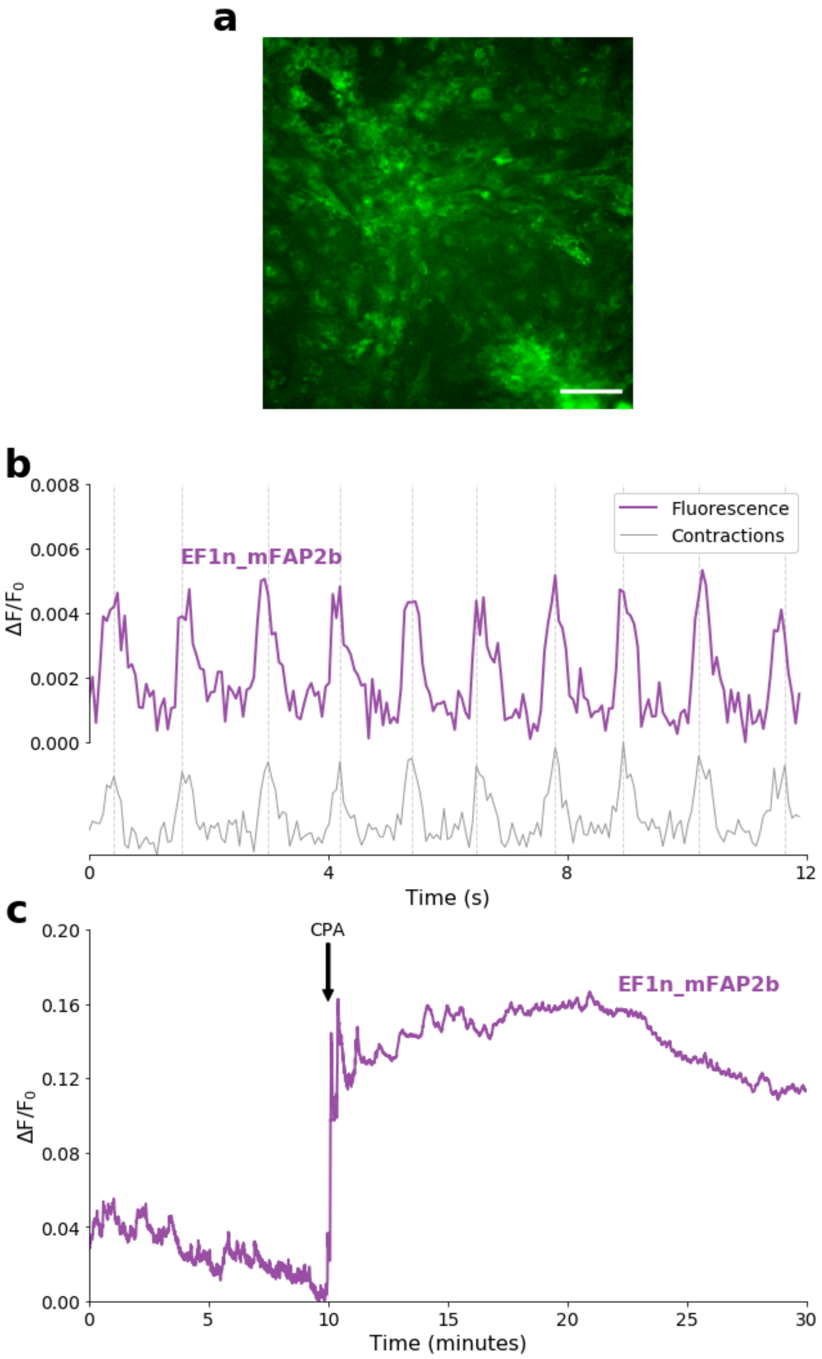
a
b
0.008
0.006
0.004
0.002
0.000
ΔF/F0
EF1n_mFAP2b
Fluorescence
Contractions
0
4
8
12
Time (s)
c
0.20
0.16
0.12
0.08
0.04
0.00
ΔF/F0
CPA
EF1n_mFAP2b
0
5
10
15
20
25
30
Time (minutes)

Supplementary Figure 19.

Time-lapse epifluorescence microscopy of human induced pluripotent stem cell (hiPSC)-derived cardiomyocytes (CMs) expressing sarcoplasmic reticulum (SR)-targeted EF1n_mFAP2b.

a, Pseudocolored maximum intensity z-axis projection from (**b**) of hiPSC-derived CMs expressing SR-targeted EF1n_mFAP2b. Scale bar represents 100 microns. Each experiment was performed once on hiPSC-derived CMs. **b**, Fluorescence imaging of live hiPSC-derived CMs expressing SR-targeted EF1n_mFAP2b *(violet)* labeled at 6.00 µM DFHBI (approximately $(K_d^+ \cdot K_d^-)^{1/2}$ for EF1n_mFAP2b) (Supplementary Table 2) imaged at ~16.7 Hz for 12 s showing whole field of view normalized fluorescence fold-change, demonstrating negative allostery *in cyto* and high photostability (compared with Figure 5f). The normalized average of 3 region of interest (ROI) traces in the fluorescence channel *(grey)* indicate the peak cardiac contraction frames *(dotted lines)*. **c**, Time-lapse epifluorescence microscopy imaging of hiPSC-derived CMs expressing SR-targeted EF1n_mFAP2b labeled at 3.00 µM DFHBI imaged at 2 Hz for 30 minutes. 20.0 µM cyclopiazonic acid (CPA) final concentration was added *(black arrow)*, which inhibits SERCA Ca^{2+} pumps blocking Ca^{2+} recovery into the SR. The negatively allosteric thermodynamic coupling between Ca^{2+} and DFHBI binding of SR-targeted EF1n_mFAP2b resulted in a sustained increase in fluorescence fold-change ($\frac{\Delta F}{F_0}$) upon CPA treatment via Ca^{2+} depletion from the SR.

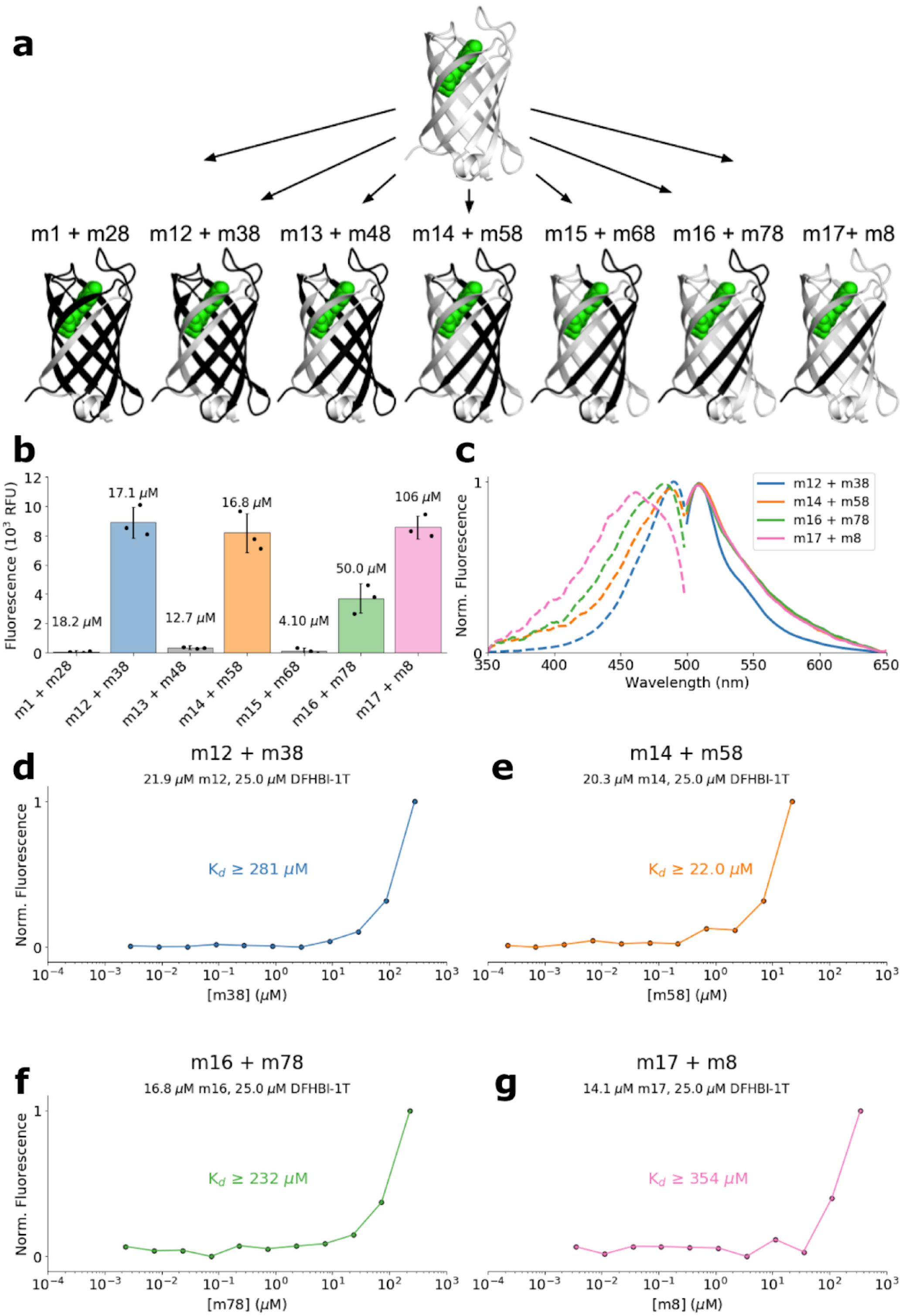

Supplementary Figure 20.

Design and biophysical characterization of split mFAP2a variants.

a, Computational model of mFAP2a *(top row)* used to design self-complementing split mFAPs *(bottom row)*. Separate polypeptide chains *(grey and black cartoons)* and the chromophore DFHBI-1T *(green spheres)* are shown. Split mFAP fragment combinations are annotated above split mFAP models *(bottom row)*, showing *(left to right)*: β-strand 1 with β-strands 2-8 *(m1 + m28)*; β-strands 1-2 with β-strands 3-8 *(m12 + m38)*; β-strands 1-3 with β-strands 4-8 *(m13 + m48)*; β-strands 1-4 with β-strands 5-8 *(m14 + m58)*; β-strands 1-5 with β-strands 6-8 *(m15 + m68)*; β-strands 1-6 with β-strands 7-8 *(m16 + m78)*; and β-strands 1-7 with β-strand 8 *(m17 + m8)*. **b**, Self-complementation of maltose binding protein (MBP)-tagged split mFAPs incubated at the annotated equimolar concentrations in 50.0 μM DFHBI-1T showing the average (n=3) fluorescence intensity. Error bars represent s.d. of the mean of 3 technical replicates. **c**, Normalized fluorescence excitation *(dotted lines)* and emission *(solid lines)* spectra (n=1) of assembled MBP-tagged split mFAP fragments. **d-g**, Titrations of MBP-tagged split mFAP fragments into their complementary MBP-tagged split mFAP fragments showing normalized fluorescence intensity in 25.0 μM DFHBI-1T *(points)*. **d**, MBP-tagged m12 was fixed at 21.9 μM final concentration as MBP-tagged m38 was titrated (n=1). **e**, MBP-tagged m14 was fixed at 20.3 μM final concentration as MBP-tagged m58 was titrated (n=1). **f**, MBP-tagged m16 was fixed at 16.8 μM final concentration as MBP-tagged m78 was titrated (n=1). **g**, MBP-tagged m17 was fixed at 14.1 μM final concentration as MBP-tagged m8 was titrated (n=1). **d-g**, The annotated thermodynamic dissociation constants (K_d values) are at least the highest concentration of titrant measured.

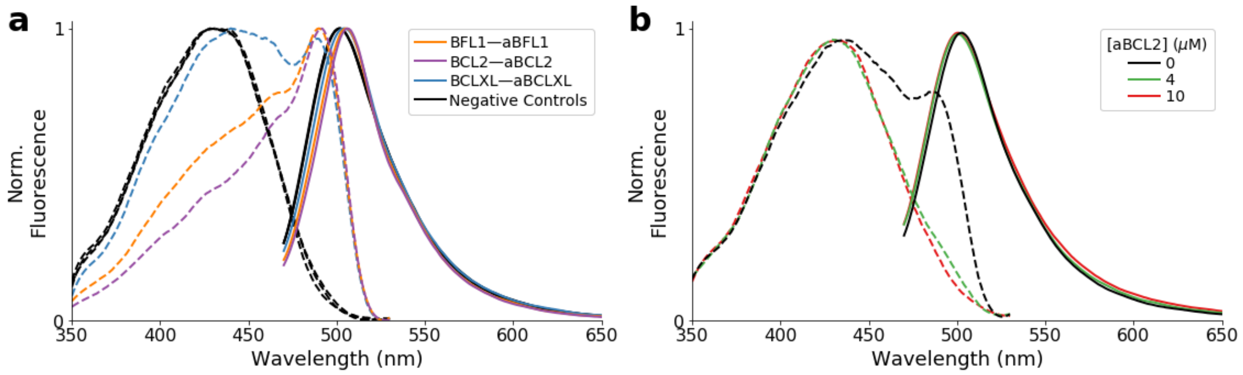

Supplementary Figure 21.

Photophysical characterization of split mFAP2a fragments m14 and m58 fused to BCL2 family proteins.

a, Normalized fluorescence excitation *(dotted lines)* and emission *(solid lines)* spectra (n=1) after equilibrium was reached in Figure 6d, in which BCLXL_m58 was pre-incubated with aBCLXL in excess DFHBI-1T before addition of m14_aBCLXL *(blue lines)* or buffer *(black lines)*. The reaction evolved analogously for BFL1–aBFL1 *(orange lines)* and BCL2–aBCL2 *(violet lines)* cognate binding partners. **b**, Normalized fluorescence excitation *(dotted lines)* and emission *(solid lines)* spectra (n=1) after equilibrium was reached in Figure 6f, in which BCL2_m58 was pre-assembled with m14_aBFL1 at 2.00 µM final concentrations in excess DFHBI-1T before addition of unfused aBCL2 at 0 µM *(black lines)*, 4.00 µM *(green lines)*, and 10.0 µM *(red lines)* final concentrations.

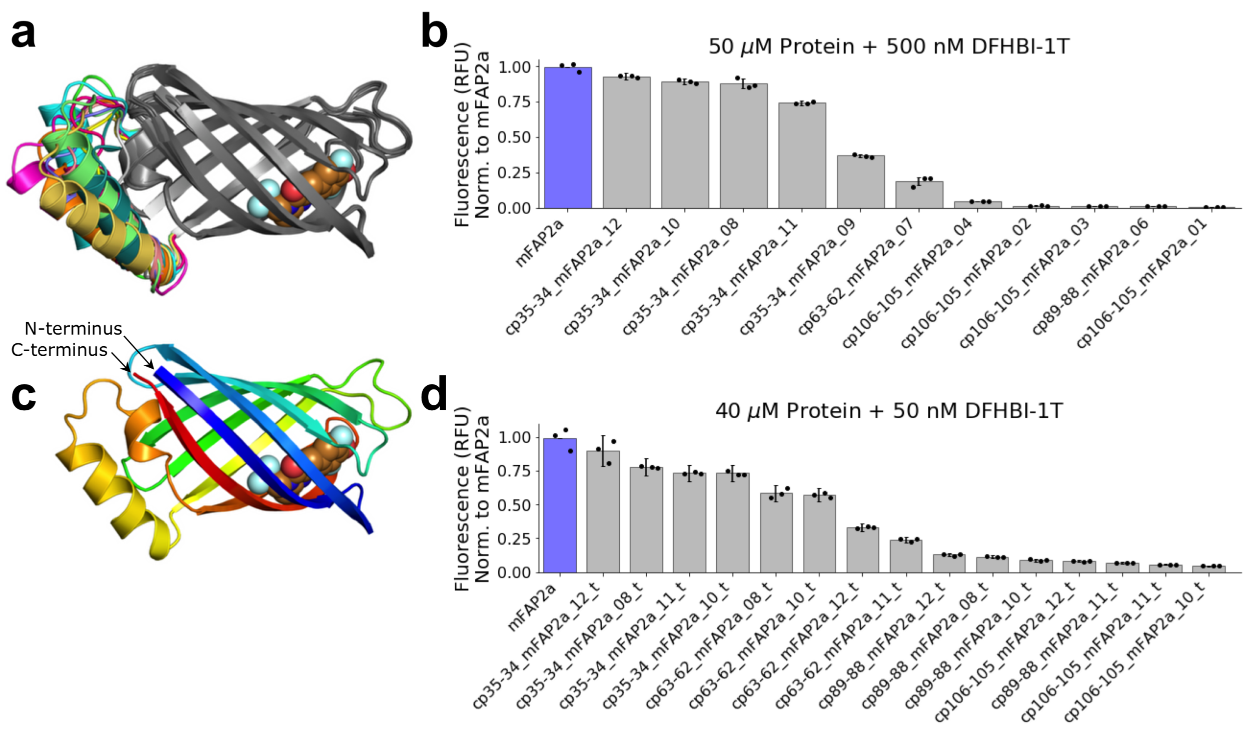

Supplementary Figure 22.

Computational design and photophysical characterization of circularly permuted mFAP (cpmFAP) variants.

a, Superimposed and overlaid computational models of *de novo* designed circularly permuted mFAP2a variants from (**b**), showing circularly permuted β-barrel protein backbones *(grey cartoons)* bound to the chromophore DFHBI-1T *(spheres)* and *de novo* designed linkers covalently fusing together the N- and C-termini of mFAP2a *(colored cartoons)*. **b**, Average fluorescence intensity of 50.0 μM cpmFAPs *(grey bars)* versus mFAP2a *(blue bar)* in 500 nM DFHBI-1T. **c**, Computational model of the cpmFAP cp35-34_mFAP2a_12, the brightest cpmFAP variant in this study (**b**,**d**), showing the protein backbone *(rainbow cartoon)* with N-terminus *(blue)* and C-terminus *(red)* bound to the chromophore DFHBI-1T *(spheres)*. **d**,

Average fluorescence intensity of 40.0 μM cpmFAP variants *(grey bars)* versus mFAP2a *(blue bar)* in 50.0 nM DFHBI-1T. **b**,**d**, Error bars represent s.d. of the mean of 3 technical replicates.

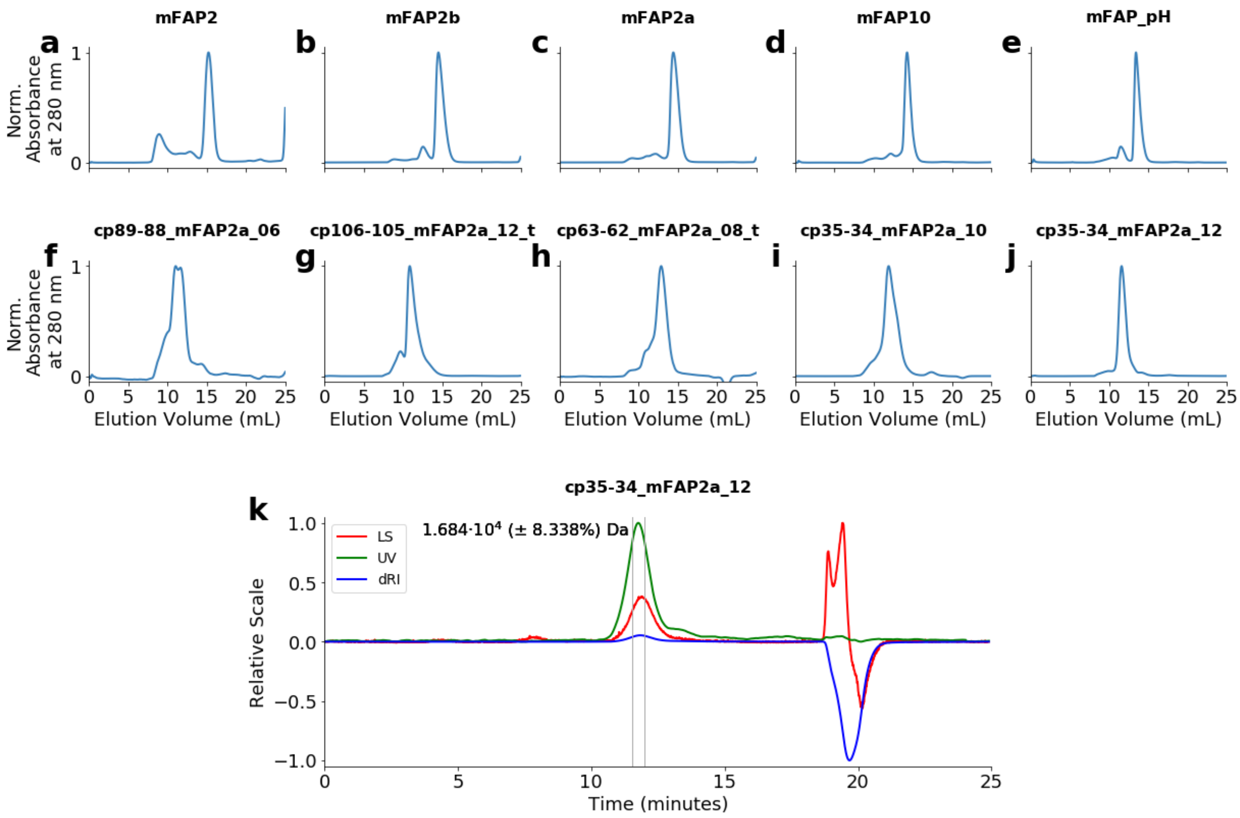

Supplementary Figure 23.

Size-exclusion chromatography (SEC) and SEC with multi-angle light scattering (MALS) of mFAP variants and circularly permuted mFAP (cpmFAP) variants.

a-**j**, SEC traces of protein samples run on a Superdex 75 Increase 10/300 GL column measuring absorbance at 280 nm (n=1), showing representative traces for 6xHis-tagged (**a**) mFAP2, (**b**) mFAP2b, (**c**) mFAP2a, (**d**) mFAP10, (**e**) mFAP_pH, (**f**) cp89-88_mFAP2a_06, (**g**) cp106-105_mFAP2a_12_t, (**h**) cp63-62_mFAP2a_08_t, (**i**) cp35-34_mFAP2a_10, and (**j**) the brightest cpmFAP tested, cp35-34_mFAP2a_12. **k**, SEC-MALS analysis (n=1) revealed a monomer peak for 6xHis-tagged cp35-34_mFAP2a_12 in which the measured molecular mass ($1.684 \cdot 10^4 \pm 8.338\%$ Da) corroborated the expected monomeric molecular mass ($1.691 \cdot 10^4$ Da),

showing light scattering (LS) signal *(red line)*, ultraviolet absorbance (UV) signal *(green line)*, and differential refractive index (dRI) signal *(blue line)*.

	10μM DFHBI	10μM DFHBI-1T	10μM DFHO	Densitometry
mFAP2b	1.0 ± 0.0	0.01 ± 0.0	0.01 ± 0.0	0.486
mFAP4	0.89 ± 0.13	0.14 ± 0.01	0.02 ± 0.0	0.449
mFAP5	0.81 ± 0.13	0.04 ± 0.0	0.01 ± 0.0	0.764
mFAP2.5	0.8 ± 0.03	0.01 ± 0.0	0.0 ± 0.0	0.641
mFAP2a	0.77 ± 0.12	0.92 ± 0.05	0.04 ± 0.0	0.388
mFAP2.4	0.69 ± 0.06	0.01 ± 0.0	0.0 ± 0.0	0.75
mFAP3	0.65 ± 0.14	0.63 ± 0.07	0.1 ± 0.01	0.289
mFAP2.2.9	0.64 ± 0.03	0.01 ± 0.0	0.0 ± 0.0	0.966
mFAP2.5.1	0.61 ± 0.02	0.01 ± 0.0	0.01 ± 0.0	0.542
mFAP7	0.52 ± 0.09	0.47 ± 0.04	0.01 ± 0.0	0.252
mFAP2.2.10	0.44 ± 0.04	0.01 ± 0.0	0.0 ± 0.0	0.884
mFAP2.5.2	0.39 ± 0.05	0.0 ± 0.0	0.0 ± 0.0	0.499
mFAP2.5.3	0.36 ± 0.02	0.01 ± 0.0	0.0 ± 0.0	0.45
mFAP2.5.4	0.34 ± 0.01	0.05 ± 0.01	0.01 ± 0.0	0.571
mFAP2.5.5	0.25 ± 0.01	0.0 ± 0.0	0.0 ± 0.0	0.542
mFAP2.2.2	0.23 ± 0.01	0.83 ± 0.08	0.02 ± 0.0	0.825
mFAP2.2.1	0.23 ± 0.02	0.81 ± 0.06	0.02 ± 0.0	0.878
mFAP2bL2	0.22 ± 0.03	0.0 ± 0.0	0.01 ± 0.0	0.191
mFAP2bL5	0.22 ± 0.02	0.0 ± 0.0	0.0 ± 0.0	0.409
mFAP2.2.3	0.21 ± 0.03	0.65 ± 0.06	0.01 ± 0.0	0.684
mFAP2.2.4	0.2 ± 0.03	0.66 ± 0.04	0.01 ± 0.0	0.654
mFAP2.2.12	0.2 ± 0.03	0.61 ± 0.04	0.01 ± 0.0	0.873
mFAP2.3	0.2 ± 0.03	0.67 ± 0.04	0.01 ± 0.0	0.796
mFAP2.0.1	0.19 ± 0.01	0.05 ± 0.01	0.0 ± 0.0	0.585
mFAP8	0.18 ± 0.02	0.0 ± 0.0	0.0 ± 0.0	0.321
mFAP2bL4	0.18 ± 0.02	0.0 ± 0.0	0.0 ± 0.0	0.346
mFAP2.2	0.17 ± 0.03	0.55 ± 0.02	0.01 ± 0.0	0.475
mFAP2.2.5	0.17 ± 0.03	0.61 ± 0.0	0.01 ± 0.0	0.64
mFAP2.2.6	0.16 ± 0.03	0.46 ± 0.03	0.01 ± 0.0	0.624
mFAP2.2.13	0.15 ± 0.03	0.5 ± 0.0	0.01 ± 0.0	0.955
mFAP6	0.15 ± 0.01	0.0 ± 0.0	0.0 ± 0.0	0.683
mFAP2	0.14 ± 0.03	0.33 ± 0.04	0.01 ± 0.0	0.433
mFAP2.2.7	0.14 ± 0.03	0.45 ± 0.01	0.01 ± 0.0	0.631
mFAP2.2.14	0.14 ± 0.03	0.28 ± 0.02	0.01 ± 0.0	0.727
mFAP1	0.13 ± 0.02	0.01 ± 0.0	0.0 ± 0.0	0.433
mFAP2bL3	0.13 ± 0.03	0.0 ± 0.0	0.0 ± 0.0	0.145
mFAP2.2.15	0.13 ± 0.03	0.27 ± 0.02	0.0 ± 0.0	0.716
mFAP2bL1	0.12 ± 0.02	0.0 ± 0.0	0.0 ± 0.0	0.204
mFAP2c.0	0.11 ± 0.03	0.33 ± 0.01	0.0 ± 0.0	0.422
mFAP_pH	0.11 ± 0.03	0.41 ± 0.02	0.01 ± 0.0	0.222
mFAP2.2.16	0.11 ± 0.02	0.29 ± 0.0	0.01 ± 0.0	0.546
mFAP2.2.8	0.1 ± 0.02	0.16 ± 0.03	0.0 ± 0.0	1.0
mFAP2a.0	0.09 ± 0.02	0.01 ± 0.0	0.0 ± 0.0	0.318
mFAP2.1	0.07 ± 0.02	0.25 ± 0.01	0.01 ± 0.0	0.475
mFAP2.2.17	0.07 ± 0.02	0.12 ± 0.01	0.0 ± 0.0	0.33
mFAP0.1	0.06 ± 0.0	0.1 ± 0.01	0.01 ± 0.0	0.788
mFAP0.2	0.03 ± 0.0	0.08 ± 0.01	0.01 ± 0.0	0.715
mFAP2c.1	0.01 ± 0.0	0.04 ± 0.01	0.0 ± 0.0	0.243
mFAP0.3	0.0 ± 0.0	0.0 ± 0.0	0.01 ± 0.0	0.765
mFAP2c.3	0.0 ± 0.0	0.01 ± 0.0	0.0 ± 0.0	0.022
mFAP2c.4	0.0 ± 0.0	0.0 ± 0.0	0.0 ± 0.0	0.0
mFAP2a.1	0.0 ± 0.0	0.0 ± 0.0	0.0 ± 0.0	0.009
mFAP2c.8	0.0 ± 0.0	0.0 ± 0.0	0.0 ± 0.0	0.027
mFAP2c.9	0.0 ± 0.0	0.0 ± 0.0	0.0 ± 0.0	0.002
mFAP2c.10	0.0 ± 0.0	0.0 ± 0.0	0.0 ± 0.0	0.001
mFAP2c.11	0.0 ± 0.0	0.0 ± 0.0	0.0 ± 0.0	0.001
mFAP2c.12	0.0 ± 0.0	0.0 ± 0.0	0.0 ± 0.0	0.004
mFAP2c.5	0.0 ± 0.0	0.0 ± 0.0	0.0 ± 0.0	0.001
mFAP2c.13	0.0 ± 0.0	0.0 ± 0.0	0.0 ± 0.0	0.002
mFAP2c.6	0.0 ± 0.0	0.0 ± 0.0	0.0 ± 0.0	0.0
mFAP2c.14	0.0 ± 0.0	0.0 ± 0.0	0.0 ± 0.0	0.001
mFAP0.4	0.0 ± 0.0	0.0 ± 0.0	0.0 ± 0.0	0.067

	100nM DFHBI	100nM DFHBI-1T	100nM DFHO	Densitometry
mFAP2a	1.0 ± 0.0	0.59 ± 0.06	0.03 ± 0.01	0.388
mFAP3	0.96 ± 0.02	0.39 ± 0.07	0.05 ± 0.02	0.289
mFAP5	0.93 ± 0.03	0.04 ± 0.01	0.01 ± 0.0	0.764
mFAP2.5	0.83 ± 0.02	0.01 ± 0.0	0.01 ± 0.0	0.641
mFAP2b	0.82 ± 0.02	0.01 ± 0.0	0.01 ± 0.0	0.486
mFAP4	0.81 ± 0.03	0.04 ± 0.01	0.01 ± 0.0	0.449
mFAP7	0.79 ± 0.04	0.29 ± 0.07	0.01 ± 0.0	0.252
mFAP2.4	0.69 ± 0.03	0.01 ± 0.0	0.01 ± 0.0	0.75
mFAP2.2.9	0.69 ± 0.01	0.02 ± 0.0	0.01 ± 0.0	0.966
mFAP2.2.10	0.61 ± 0.01	0.01 ± 0.0	0.01 ± 0.0	0.884
mFAP2.5.2	0.55 ± 0.03	0.01 ± 0.0	0.01 ± 0.0	0.499
mFAP2.5.1	0.4 ± 0.04	0.01 ± 0.0	0.01 ± 0.0	0.542
mFAP6	0.29 ± 0.04	0.01 ± 0.0	0.01 ± 0.0	0.683
mFAP2.5.4	0.22 ± 0.0	0.02 ± 0.0	0.01 ± 0.0	0.571
mFAP2.0.1	0.22 ± 0.0	0.04 ± 0.0	0.01 ± 0.0	0.585
mFAP2.5.5	0.21 ± 0.02	0.01 ± 0.0	0.01 ± 0.0	0.542
mFAP1	0.18 ± 0.0	0.01 ± 0.0	0.01 ± 0.0	0.433
mFAP2.5.3	0.16 ± 0.04	0.01 ± 0.0	0.01 ± 0.0	0.45
mFAP_pH	0.16 ± 0.0	0.2 ± 0.0	0.01 ± 0.0	0.222
mFAP2.2.4	0.15 ± 0.0	0.5 ± 0.02	0.02 ± 0.0	0.654
mFAP2.3	0.15 ± 0.0	0.52 ± 0.02	0.02 ± 0.0	0.796
mFAP2.2.3	0.15 ± 0.0	0.43 ± 0.02	0.02 ± 0.0	0.684
mFAP2.2.1	0.15 ± 0.0	0.59 ± 0.0	0.02 ± 0.0	0.878
mFAP2.2.2	0.15 ± 0.0	0.54 ± 0.01	0.02 ± 0.0	0.825
mFAP2c.0	0.14 ± 0.0	0.19 ± 0.01	0.01 ± 0.0	0.422
mFAP2.2.15	0.14 ± 0.0	0.3 ± 0.0	0.01 ± 0.0	0.716
mFAP2bL5	0.14 ± 0.02	0.01 ± 0.0	0.01 ± 0.0	0.409
mFAP2.2.5	0.14 ± 0.0	0.46 ± 0.01	0.02 ± 0.0	0.64
mFAP2.2.16	0.14 ± 0.0	0.34 ± 0.0	0.02 ± 0.0	0.546
mFAP2.2.13	0.14 ± 0.0	0.54 ± 0.0	0.03 ± 0.0	0.955
mFAP2.2.17	0.14 ± 0.0	0.1 ± 0.01	0.01 ± 0.0	0.33
mFAP2.2.12	0.13 ± 0.0	0.47 ± 0.0	0.02 ± 0.0	0.873
mFAP2.1	0.13 ± 0.0	0.34 ± 0.01	0.01 ± 0.0	0.475
mFAP2.2	0.13 ± 0.0	0.33 ± 0.02	0.01 ± 0.0	0.475
mFAP2a.0	0.13 ± 0.02	0.01 ± 0.0	0.01 ± 0.0	0.318
mFAP2.2.6	0.13 ± 0.0	0.35 ± 0.01	0.02 ± 0.0	0.624
mFAP2.2.14	0.13 ± 0.0	0.26 ± 0.01	0.01 ± 0.0	0.727
mFAP2.2.7	0.13 ± 0.0	0.41 ± 0.01	0.02 ± 0.0	0.631
mFAP2	0.12 ± 0.0	0.15 ± 0.01	0.01 ± 0.0	0.433
mFAP2bL2	0.11 ± 0.02	0.01 ± 0.0	0.01 ± 0.0	0.191
mFAP2c.1	0.1 ± 0.0	0.22 ± 0.01	0.01 ± 0.0	0.243
mFAP2.2.8	0.1 ± 0.0	0.27 ± 0.01	0.01 ± 0.0	1.0
mFAP2bL1	0.07 ± 0.02	0.01 ± 0.0	0.01 ± 0.0	0.204
mFAP2bL4	0.06 ± 0.01	0.01 ± 0.0	0.01 ± 0.0	0.346
mFAP2bL3	0.05 ± 0.01	0.01 ± 0.0	0.01 ± 0.0	0.145
mFAP8	0.04 ± 0.01	0.01 ± 0.0	0.01 ± 0.0	0.321
mFAP0.1	0.02 ± 0.0	0.06 ± 0.0	0.02 ± 0.0	0.788
mFAP2c.9	0.02 ± 0.01	0.01 ± 0.0	0.01 ± 0.0	0.002
mFAP2a.1	0.02 ± 0.0	0.01 ± 0.0	0.01 ± 0.0	0.009
mFAP2c.8	0.02 ± 0.0	0.01 ± 0.0	0.01 ± 0.0	0.027
mFAP2c.3	0.01 ± 0.0	0.01 ± 0.0	0.01 ± 0.0	0.022
mFAP2c.4	0.01 ± 0.0	0.01 ± 0.0	0.01 ± 0.0	0.0
mFAP2c.10	0.01 ± 0.0	0.01 ± 0.0	0.01 ± 0.0	0.001
mFAP0.2	0.01 ± 0.0	0.04 ± 0.0	0.01 ± 0.0	0.715
mFAP2c.13	0.01 ± 0.0	0.01 ± 0.0	0.01 ± 0.0	0.002
mFAP2c.11	0.01 ± 0.0	0.01 ± 0.0	0.01 ± 0.0	0.001
mFAP2c.5	0.01 ± 0.0	0.01 ± 0.0	0.01 ± 0.0	0.001
mFAP2c.14	0.0 ± 0.0	0.01 ± 0.0	0.01 ± 0.0	0.001
mFAP2c.6	0.0 ± 0.0	0.01 ± 0.0	0.01 ± 0.0	0.0
mFAP2c.12	0.0 ± 0.0	0.01 ± 0.0	0.01 ± 0.0	0.004
mFAP0.4	0.0 ± 0.0	0.01 ± 0.0	0.01 ± 0.0	0.067
mFAP0.3	0.0 ± 0.0	0.01 ± 0.0	0.02 ± 0.0	0.765

	DFHBI K_d (μM)	DFHBI-1T K_d (μM)	DFHO K_d (μM)
mFAP2c.13	< 0.5 (0.01 ± 0.01)	79.50 ± 29.51	42.34 ± 23.82
mFAP2a	0.05 ± 0.01	3.70 ± 0.56	8.47 ± 1.99
mFAP2c.5	< 0.5 (0.09 ± 0.02)	1.19 ± 0.22	3.98 ± 1.54
mFAP3	0.10 ± 0.06	2.34 ± 0.38	3.56 ± 1.81
mFAP2c.11	< 0.5 (0.11 ± 0.02)	0.87 ± 0.12	4.00 ± 1.21
mFAP2.2.13	< 0.5 (0.12 ± 0.03)	1.06 ± 0.18	3.78 ± 3.10
mFAP2c.9	< 0.5 (0.13 ± 0.02)	1.68 ± 0.23	6.06 ± 1.97
mFAP2c.12	< 0.5 (0.14 ± 0.02)	0.73 ± 0.09	3.43 ± 1.32
mFAP2.3	< 0.5 (0.15 ± 0.02)	1.58 ± 0.34	10.41 ± 3.59
mFAP2.2.12	< 0.5 (0.15 ± 0.02)	2.00 ± 0.31	5.83 ± 4.89
mFAP2.2.17	< 0.5 (0.15 ± 0.02)	3.11 ± 0.38	4.27 ± 4.16
mFAP2.2.7	< 0.5 (0.15 ± 0.03)	1.32 ± 0.22	0.74 ± 0.77
mFAP2.2.1	< 0.5 (0.16 ± 0.03)	1.66 ± 0.34	< 0.5 (0.01 ± 0.01)
mFAP2.2.16	< 0.5 (0.16 ± 0.03)	3.11 ± 0.43	2.35 ± 2.35
mFAP2c.10	< 0.5 (0.16 ± 0.03)	3.38 ± 0.87	8.94 ± 2.65
mFAP_pH	< 0.5 (0.16 ± 0.04)	1.50 ± 0.31	10.47 ± 4.57
mFAP2c.3	< 0.5 (0.18 ± 0.03)	1.28 ± 0.26	10.98 ± 4.99
mFAP2c.4	< 0.5 (0.19 ± 0.02)	4.07 ± 0.45	8.60 ± 1.45
mFAP2.2.2	< 0.5 (0.20 ± 0.03)	1.42 ± 0.28	13.26 ± 7.38
mFAP2c.0	< 0.5 (0.20 ± 0.03)	2.39 ± 0.49	10.19 ± 3.05
mFAP2.2.15	< 0.5 (0.21 ± 0.03)	3.28 ± 0.79	4.67 ± 4.43
mFAP2c.1	< 0.5 (0.21 ± 0.02)	0.90 ± 0.17	7.85 ± 2.69
mFAP2.2.3	< 0.5 (0.21 ± 0.04)	1.85 ± 0.36	5.96 ± 3.54
mFAP2.2.14	< 0.5 (0.21 ± 0.04)	3.27 ± 0.54	8.41 ± 6.83
mFAP2.2.4	< 0.5 (0.23 ± 0.06)	1.91 ± 0.50	5.17 ± 2.90
mFAP2.2.6	< 0.5 (0.24 ± 0.03)	2.13 ± 0.33	6.17 ± 3.07
mFAP2c.8	< 0.5 (0.27 ± 0.05)	1.89 ± 0.38	5.25 ± 1.98
mFAP4	< 0.5 (0.28 ± 0.08)	20.50 ± 2.85	12.82 ± 4.05
mFAP7	< 0.5 (0.34 ± 0.09)	3.71 ± 0.33	16.07 ± 3.93
mFAP2a.1	< 0.5 (0.43 ± 0.02)	3.39 ± 0.45	6.69 ± 1.50
mFAP2c.14	< 0.5 (0.46 ± 0.77)	16.39 ± 13.03	55.04 ± 23.95
mFAP1	< 0.5 (0.50 ± 0.09)	10.80 ± 1.00	7.80 ± 2.89
mFAP5	0.88 ± 0.14	48.90 ± 7.78	28.87 ± 13.17
mFAP2.2.9	0.95 ± 0.05	21.68 ± 3.99	1.95 ± 2.11
mFAP2.2.10	1.04 ± 0.15	19.52 ± 5.39	< 0.5 (0.00 ± 0.01)
mFAP2.5	1.13 ± 0.09	52.54 ± 7.48	34.23 ± 13.69
mFAP2.2	1.15 ± 0.16	43.16 ± 7.01	22.72 ± 9.66
mFAP2.4	1.31 ± 0.10	38.74 ± 5.71	32.73 ± 14.21
mFAP2.5.2	1.36 ± 0.17	40.47 ± 4.26	27.73 ± 5.72
mFAP2bL2	1.41 ± 0.10	N/D	N/D
mFAP2.5.4	1.64 ± 0.14	39.16 ± 4.33	23.46 ± 7.34
mFAP2b	1.67 ± 0.08	42.20 ± 6.03	9.01 ± 1.91
mFAP2.2.5	1.72 ± 0.22	N/D	N/D
mFAP2a.0	1.90 ± 0.23	43.25 ± 6.34	< 0.5 (0.01 ± 0.02)
mFAP0.1	2.26 ± 1.37	0.85 ± 0.16	23.47 ± 19.09
mFAP2bL5	2.70 ± 0.15	N/D	N/D
mFAP2.5.1	4.43 ± 0.44	71.74 ± 13.55	32.52 ± 18.50
mFAP2bL1	4.59 ± 0.48	N/D	N/D
mFAP2.5.5	5.53 ± 0.59	66.60 ± 12.29	39.18 ± 13.38
mFAP2bL3	5.93 ± 0.39	N/D	N/D
mFAP2.5.3	9.47 ± 0.72	91.84 ± 20.65	< 0.5 (0.00 ± 0.01)
mFAP2bL4	13.04 ± 0.82	N/D	N/D
mFAP6	13.14 ± 1.71	< 0.5 (0.00 ± 0.01)	< 0.5 (0.00 ± 0.00)
mFAP8	58.12 ± 8.61	40.51 ± 14.87	46.41 ± 31.00
mFAP2c.6	150.14 ± 264.69	98.69 ± 65.59	41.64 ± 8.08

Supplementary Table 1.

Relative fluorescence intensity, relative expression level, and chromophore affinities of mFAP variants.

Fluorescence intensity of the deprotonated state of each chromophore at 10.0 μM (left) and 100 nM (middle) chromophore concentrations were measured at two purified protein volumes (25.0 μL and 50.0 μL). Fluorescence intensities were normalized from 0 to 1 across all three chromophores (DFHBI, DFHBI-1T, and DFHO) for each chromophore concentration and each protein volume, and the normalized values for the two protein volumes tested for each chromophore at each chromophore concentration were averaged and s.d. of the mean computed. Reported values are the normalized averages and s.d. of the means for each chromophore at each chromophore concentration in descending order of fluorescence intensity at each chromophore concentration. Heatmap grayscale colors aid visualization of the brightest variants per chromophore concentration per chromophore relative to the brightest variant per chromophore concentration. Reported densitometry values are the relative densitometry values normalized from 0 to 1 and represent how well each design expresses relative to one another in *E. coli* cultures. Thermodynamic dissociation constants *(right)* are obtained by non-linear least squares fitting of fluorescence intensity measurements normalized from 0 to 1 of chromophore titrations (n=1) to a single binding site isotherm function, and the K_d values reported with the s.d. of the fit. Where the obtained K_d values are below the protein concentration tested, the K_d and s.d. of the fit are reported in parentheses. Values with "N/D" were not determined. Heatmap grayscale colors aid visualization of the spread of K_d values for each chromophore.

Design Name	DFHBI K_d^+ (μM)	DFHBI K_d^- (μM)	Ca^{2+} K_d (μM)	Hill Coefficient, n_H	K_d^-/K_d^+	K_d^+/K_d^-	$(K_d^+ \cdot K_d^-)^{1/2}$ (μM)	Max. $\lvert\theta^+ - \theta^-\rvert$ (%)	Dynamic Range at K_d^+/2 μM DFHBI	Dynamic Range at K_d^-/2 μM DFHBI
EF1p_mFAP2b	2.83 ± 0.15	16.6 ± 1.3	$4{,}110 \pm 640$	1.31 ± 0.19	5.85 ± 0.56	-	6.86 ± 0.33	41.5	9.32	-
EF1p_mFAP2a	1.07 ± 0.023	33.3 ± 3.2	352 ± 11	0.944 ± 0.022	31.2 ± 3.1	-	5.97 ± 0.3	69.6	16.1	-
EF1p2_mFAP2b	10.8 ± 1.1	34.8 ± 3.3	$2{,}270 \pm 290$	0.852 ± 0.081	3.22 ± 0.46	-	19.4 ± 1.4	28.4	17.3	-
EF1p2_mFAP2a	1.27 ± 0.054	45.0 ± 5.3	273 ± 3.9	0.982 ± 0.012	35.5 ± 4.4	-	7.55 ± 0.47	71.3	2.99	-
EF1p3_mFAP2b	3.41 ± 0.21	27.1 ± 2.2	$1{,}720 \pm 81$	1.03 ± 0.045	7.94 ± 0.82	-	9.62 ± 0.49	47.6	18.6	-
EF1p3_mFAP2a	1.54 ± 0.067	56.2 ± 8.0	588 ± 14	0.948 ± 0.012	36.5 ± 5.5	-	9.30 ± 0.7	71.6	18.7	-
EF1n_mFAP2b	6.94 ± 0.27	5.03 ± 0.32	260 ± 29	1.20 ± 0.12	-	1.38 ± 0.10	5.91 ± 0.22	8.02	-	2.79
EF1n_mFAP2a	19.9 ± 1.6	2.64 ± 0.11	$3{,}020 \pm 200$	0.881 ± 0.038	-	7.54 ± 0.69	7.25 ± 0.33	46.6	-	2.71
EF1n2_mFAP2b	42.2 ± 8.0	27.6 ± 2.2	140 ± 14	1.06 ± 0.10	-	1.53 ± 0.31	34.1 ± 3.5	10.6	-	2.13
EF1n2_mFAP2a	43.6 ± 4.8	4.68 ± 0.28	504 ± 26	0.883 ± 0.023	-	9.32 ± 1.2	14.3 ± 0.9	50.7	-	2.28
EF1n3_mFAP2b	26.9 ± 2.2	3.21 ± 0.13	125 ± 78	0.843 ± 0.46	-	8.37 ± 0.76	9.30 ± 0.42	48.6	-	1.48
EF1n3_mFAP2a	5.42 ± 0.22	1.25 ± 0.062	241 ± 13	0.951 ± 0.045	-	4.32 ± 0.28	2.61 ± 0.084	35.0	-	1.54
EF2n_mFAP2b	38.0 ± 3.6	10.0 ± 0.65	41.9 ± 5.9	1.45 ± 0.13	-	3.79 ± 0.44	19.5 ± 1.1	32.1	-	3.40
EF2n_mFAP2a	51.9 ± 14	2.36 ± 0.15	60.0 ± 4.0	1.14 ± 0.059	-	21.9 ± 6.2	11.1 ± 1.6	64.8	-	3.78
EF2n2_mFAP2b	14.5 ± 9.7	14.6 ± 2.7	162 ± 38	1.27 ± 0.29	-	0.994 ± 0.69	14.5 ± 5.1	0.154	-	2.93
EF2n2_mFAP2a	45.7 ± 13	2.50 ± 0.085	226 ± 15	1.07 ± 0.066	-	18.2 ± 5.2	10.7 ± 1.5	62.0	-	1.47
EF2n3_mFAP2b	96.6 ± 25	27.4 ± 2.3	167 ± 26	1.07 ± 0.16	-	3.53 ± 0.96	51.4 ± 7.0	30.5	-	8.02
EF2n3_mFAP2a	85.7 ± 24	9.09 ± 0.54	192 ± 7.1	1.04 ± 0.034	-	9.43 ± 2.7	27.9 ± 4.0	50.9	-	1.61
EF4n_mFAP2b	$65.1 \pm 10^{*}$	$28.9 \pm 2.6^{\dagger}$	$7.02 \pm 0.56^{*}$	0.951 ± 0.067	-	2.25 ± 0.41	43.4 ± 4.0	20.1	-	13.0
EF4n_mFAP2a	$69.9 \pm 12^{*}$	$62.7 \pm 10^{\dagger}$	$9.52 \pm 1.7^{*}$	0.823 ± 0.12	-	1.12 ± 0.26	66.2 ± 7.8	2.73	-	1.86

Supplementary Table 2.

Thermodynamic and photophysical properties of Ca^{2+}-responsive mFAPs.

Thermodynamic dissociation constants for DFHBI in excess Ca^{2+} (K_d^+) and in absence of Ca^{2+} (K_d^-) were computed by fitting the normalized fluorescence intensity measurements (n=1) from DFHBI titrations to a single binding site isotherm function with Hill coefficient of 1 using non-linear least squares fitting, with s.d. of the fit reported. Thermodynamic dissociation constants for Ca^{2+} in excess DFHBI (Ca^{2+} K_d) are computed by fitting the normalized average fluorescence intensity measurements (n=3) from Ca^{2+} titrations to a single binding site isotherm function (for positively allosteric Ca^{2+}-responsive mFAPs) or to an inverse single binding site isotherm function (for negatively allosteric Ca^{2+}-responsive mFAPs) with Hill coefficient of 1 using non-linear least squares fitting, with s.d. of the fit reported. Hill coefficients were computed by fitting the Ca^{2+} titration normalized average fluorescence endpoint measurements to a single binding site isotherm function (for positively allosteric Ca^{2+}-responsive mFAPs) or to an inverse single binding site isotherm function (for negatively allosteric Ca^{2+}-responsive mFAPs) with Hill coefficient as a free parameter using non-linear least squares fitting, with the s.d. of the

fit reported. For positively allosteric Ca^{2+}-responsive mFAPs, K_d^-/K_d^+ is reported, and for negatively allosteric Ca^{2+}-responsive mFAPs, K_d^+/K_d^- is reported. For all Ca^{2+}-responsive mFAPs, $(K_d^+ \cdot K_d^-)^{1/2}$ is reported (see Supplementary Figure 14). The maximum absolute values of the difference in ratio of sensor bound by DFHBI to total sensor in excess Ca^{2+} and in absence of Ca^{2+}, Max. $\left|\theta^+ - \theta^-\right|$, is reported as a percent (see Supplementary Note 1). The fluorescence dynamic ranges (F_{max}/F_{min}) are calculated (n=1) using fluorescence emission intensities at peak emission wavelengths of sensors in 750 mM $CaCl_2$ and 1.50 mM EGTA for positively allosteric Ca^{2+}-responsive mFAPs at $\frac{K_d^+}{2}$ μM DFHBI and for negatively allosteric Ca^{2+}-responsive mFAPs at $\frac{K_d^-}{2}$ μM DFHBI (Supplementary Figure 9). We expect that under various experimental conditions, such as different chromophore concentrations (see Supplementary Figure 14) or the presence of free competitive inhibitor ions[9,10] known to bind EF-hand motifs (e.g. Mg^{2+}, Tb^{3+}, La^{3+}), the thermodynamic dissociation constants for DFHBI and Ca^{2+} will be altered. Therefore, characterization of the thermodynamic properties of Ca^{2+}-responsive mFAPs under various systems of interest should be performed. * Chelex 100 was used to pre-treat buffers. † Titrations carried out in EGTA.

Data Collection		Refinement	
Space group	P 1 21 1	R-work	0.1691
Unit cell		R-free	0.2010
a, b, c	36.4, 35.6, 86.6	Number of non-hydrogen atoms	2,160
alpha, beta, gamma	90, 90.7, 90	macromolecules	1,904
Wavelength (Å)	1.54	ligands	38
Resolution range (Å)	50-2.1 (2.2-2.1)	water	218
Unique reflections	13,070	Protein residues	254
R-merge	0.025 (0.055)	RMS(bonds)	0.004
R-meas	0.030 (0.068)	RMS(angles)	0.83
R-pim	0.016 (0.040)	Ramachandran favored (%)	98.00
CC1/2	(0.996)	Ramachandran allowed (%)	2.00
I/sigma(I)	35.2 (14.7)	Ramachandran outliers (%)	0.00
Chi^2	0.595	Clashscore	4.27
Multiplicity	3.6 (2.8)	Average B-factor	20.11
Completeness (%)	98.9 (86.2)	macromolecules	19.33
Wilson B-factor	18.62	ligands	14.58
		solvent	27.9

Supplementary Table 3.

X-ray crystallography data collection and refinement metrics for the EF1p2_mFAP2b–DFHBI–Ca^{2+} co-crystal structure (PDB accession code 6OHH).

Supplementary Note 1.

Theoretical thermodynamic properties of Ca^{2+}-responsive mFAPs.

We propose that the discussion of theoretical thermodynamics of analyte-dependent fluorogenic proteins as previously presented[11] applies to the suite of positively allosteric Ca^{2+}-responsive mFAPs presented in this study, and we further extend this discussion to negatively allosteric analyte-dependent fluorogenic proteins. In the presence of excess fluorogen, the fraction of protein–fluorogen complex to total protein, θ, is given by

$$\theta = \frac{F_{tot}}{K_d + F_{tot}} \tag{1}$$

where F_{tot} is the total concentration of fluorogen and K_d is the thermodynamic dissociation constant of protein–fluorogen complex. In the presence of excess analyte, one can model the fraction of protein–fluorogen complex to total protein, θ^+, as:

$$\theta^+ = \frac{F_{tot}}{K_d^+ + F_{tot}} \tag{2}$$

where K_d^+ is the thermodynamic dissociation constant of protein–fluorogen complex in the presence of excess analyte. Conversely, in the absence of analyte, one can model the fraction of protein–fluorogen complex to total protein, θ^-, as:

$$\theta^- = \frac{F_{tot}}{K_d^- + F_{tot}} \tag{3}$$

where K_d^- is the thermodynamic dissociation constant of protein–fluorogen complex in the absence of analyte.

For positively allosteric analyte-dependent fluorogenic proteins, where analyte binding increases the affinity of fluorogen to protein, one can characterize the fluorescence response

upon analyte binding by computing the difference in protein–fluorogen complex to total protein in excess or in absence of analyte as the following:

$$\theta^{+} - \theta^{-} = \frac{F_{tot}}{K_d^{+} + F_{tot}} - \frac{F_{tot}}{K_d^{-} + F_{tot}} \tag{4}$$

For negatively allosteric analyte-dependent fluorogenic proteins, where analyte binding decreases the affinity of fluorogen to protein, one can characterize the fluorescence response upon analyte binding by computing the difference in protein–fluorogen complex to total protein in absence or in excess of analyte as the following:

$$\theta^{-} - \theta^{+} = \frac{F_{tot}}{K_d^{-} + F_{tot}} - \frac{F_{tot}}{K_d^{+} + F_{tot}} \tag{5}$$

In both positive and negative allosteric modulation, it follows that the maximum variational change in protein–fluorogen complex to total protein upon analyte binding is given by:

$$F_{tot} = (K_d^{+} \cdot K_d^{-})^{1/2} \tag{6}$$

Additionally, for positively allosteric analyte-dependent fluorogenic proteins, one can characterize the fluorescence response upon analyte binding by computing the fractional increase in protein–fluorogen complex to total protein as the following:

$$\frac{\theta^{+}}{\theta^{-}} = \frac{\dfrac{F_{tot}}{K_d^{+} + F_{tot}}}{\dfrac{F_{tot}}{K_d^{-} + F_{tot}}} \tag{7}$$

Similarly, for negatively allosteric analyte-dependent fluorogenic proteins, one can characterize the fluorescence response upon analyte binding by computing the fractional decrease in protein–fluorogen complex to total protein as the following:

$$\frac{\theta^-}{\theta^+} = \frac{\frac{F_{tot}}{K_d^- + F_{tot}}}{\frac{F_{tot}}{K_d^+ + F_{tot}}} \quad (8)$$

For both positive and negative allosteric modulation, it follows that $\frac{\theta^+}{\theta^-}$ and $\frac{\theta^-}{\theta^+}$ are maximal at exceedingly low F_{tot}, approaching $\frac{K_d^-}{K_d^+}$ for positive allosteric modulation and approaching $\frac{K_d^+}{K_d^-}$ for negative allosteric modulation. Furthermore, by considering the fluorescence intensities of an analyte-dependent fluorogenic protein in excess or in absence of analyte as I^+ or I^-, respectively, and brightnesses (given by quantum yield multiplied by extinction coefficient) in excess or in absence of analyte as B^+ or B^-, respectively, the fluorescence dynamic range of a positively allosteric analyte-dependent fluorogenic protein is given by

$$\frac{I^+}{I^-} = \frac{B^+ \cdot \theta^+}{B^- \cdot \theta^-} \quad (9)$$

and the fluorescence dynamic range of a negatively allosteric analyte-dependent fluorogenic protein is given by

$$\frac{I^-}{I^+} = \frac{B^- \cdot \theta^-}{B^+ \cdot \theta^+} \quad (10)$$

Therefore, the dynamic range of an analyte-dependent fluorogenic protein is directly proportional to $\frac{\theta^+}{\theta^-}$ for positive allosteric modulation and $\frac{\theta^-}{\theta^+}$ for negative allosteric modulation, which is experimentally optimized as F_{tot} decreases. However, F_{tot} must be high enough to achieve a detectable fluorescence signal without compromising fluorescence dynamic range of the sensor. As previously proposed for positively allosteric analyte-dependent fluorogenic

proteins[11], we concur that an ideal compromise to obtain both good dynamic range and satisfactory detection sensitivity is to choose F_{tot} in the range:

$$\frac{K_d^+}{2} \leq F_{tot} \leq (K_d^+ \cdot K_d^-)^{1/2} \tag{11}$$

We extend this suggestion by proposing that for negatively allosteric analyte-dependent fluorogenic proteins, an ideal compromise to obtain both good dynamic range and satisfactory detection sensitivity is to choose F_{tot} in the range:

$$\frac{K_d^-}{2} \leq F_{tot} \leq (K_d^- \cdot K_d^+)^{1/2} \tag{12}$$

The total fluorogen concentration may be tuned according to the experimental requirements, ultimately choosing lower fluorogen concentrations to improve overall fluorescence dynamic range of the sensor, and choosing higher fluorogen concentrations to improve overall fluorescence signal and photostability in both the presence and absence of analyte. Experimentally determined K_d^+ and K_d^-, and computed $(K_d^+ \cdot K_d^-)^{1/2}$, for positively allosteric and negatively allosteric Ca^{2+}-responsive mFAPs are reported in Supplementary Table 2 with further analysis of thermodynamic properties presented in Supplementary Figure 14.

Supplementary Note 2.

Custom RosettaScripts XML script used to design extended loops onto loop7 of mFAP2b.

Rosetta build specifications:

Git tag: `v2018.12-dev60119`

Operating system: Linux

Compiler: gcc (Ubuntu 5.4.0-6ubuntu1~16.04.4) 5.4.0 20160609

Example RosettaScripts[12] command line:

```
rosetta_scripts.hdf5.linuxgccrelease @flags -s mFAP2b.pdb
-parser:script_vars bp=mFAP2b_cutpoints_71_77_L3A_0035.bp
```

Example blueprint file (e.g. `mFAP2b_cutpoints_71_77_L3A_0035.bp`*):*

```
1 S LE .
2 R HA .
3 A HA .
4 A HA .
5 Q HA .
6 L HA .
7 L LA .
8 P LB .
9 G EE .
10 T EB .
11 W EB .
12 Q EB .
13 V EB .
14 T EB .
15 M EB .
16 T EB .
17 N EB .
18 E LA .
19 D LA .
20 G LG .
21 Q LB .
```

22 T EB .
23 S EB .
24 Q EB .
25 G EE .
26 Q EB .
27 W EB .
28 H EB .
29 F EB .
30 Q EB .
31 P EB .
32 R EA .
33 S EB .
34 P LA .
35 Y LA .
36 T EB .
37 M EB .
38 D EB .
39 I EB .
40 V EB .
41 A EB .
42 Q EB .
43 G EE .
44 T EB .
45 I EB .
46 S LG .
47 D LG .
48 G LG .
49 R EB .
50 P EB .
51 I EB .
52 V EB .
53 G EB .
54 Y EB .
55 G EB .
56 K EB .
57 A EB .
58 T EB .
59 V EB .
60 K EA .
61 T EB .
62 P LA .
63 D LA .
64 T EB .
65 L EB .
66 D EB .

67 I EB .
68 D EB .
69 I EB .
70 T EB .
71 Y EB R
0 E LE R
0 G EB R
0 K LA R
0 S LB R
0 G HE R
0 S HA R
0 L HA R
0 A EB R
0 G LG R
0 S LB R
0 K LB R
0 I LO R
77 I EB R
78 K EB .
79 A EB .
80 Q EB .
81 G EE .
82 Q EB .
83 I EB .
84 T EB .
85 M EB .
86 D EA .
87 S EB .
88 P LA .
89 T LA .
90 Q EB .
91 F EB .
92 K EB .
93 W EB .
94 D EB .
95 A EB .
96 T EB .
97 T LA .
98 K LB .
99 G LG .
100 E LB .
101 N LE .
102 D LA .
103 F LB .
104 H LB .

```
105 G LE .
106 R EB .
107 L EB .
108 T EB .
109 G EB .
110 T EB .
111 L EB .
112 Q EB .
113 R EB .
114 Q LB .
115 E LO .
116 Z L  .
```

Example RosettaScripts[12] command line flags file:

```
-parser:protocol Supplementary_Note_2.xml
-out:level 100
-out:path:all output
-scorefile score.sc
-extra_res_fa HBI.fa.params
-overwrite
-write_all_connect_info 1
-run::preserve_header
-connect_info_cutoff 3.0
-nblist_autoupdate
-jd2:ntrials 1
-packing::use_input_sc 1
-packing::extrachi_cutoff 1
-packing::ex1
-packing::ex2
-linmem_ig 10
-parser_read_cloud_pdb 1
-ignore_unrecognized_res
-no_optH false
-flip_HNQ
-no_his_his_pairE
-enzdes:detect_design_interface
-chemical:exclude_patches LowerDNA  UpperDNA Cterm_amidation
VirtualBB ShoveBB VirtualDNAPhosphate VirtualNTerm CTermConnect
sc_orbitals pro_hydroxylated_case1 pro_hydroxylated_case2
ser_phosphorylated thr_phosphorylated  tyr_phosphorylated
tyr_sulfated lys_dimethylated lys_monomethylated
lys_trimethylated lys_acetylated glu_carboxylated cys_acetylated
```

```
tyr_diiodinated N_acetylated C_methylamidated
MethylatedProteinCterm
-holes:dalphaball DAlphaBall.gcc
```

To download the RosettaScripts[12] XML script to build extended loop7 designs

(`Supplementary_Note_2.xml`):

```
git clone https://github.com/klimaj/mFAPs.git
```

Rosetta loopmodel application sampling.

Rosetta build specifications:

Git tag: `v2018.22-dev60238`

Operating system: Linux

Compiler: gcc (Ubuntu 5.4.0-6ubuntu1~16.04.4) 5.4.0 20160609

Example command line execution:

```
~/loopmodel.hdf5.linuxgccrelease @flags -s

mFAP2b_singleloop_p01wC6_mFAP2b_0001.pdb -out:file:silent

silent_107.out
```

Example command line flags file:

```
-nstruct 1
-extra_res_fa HBI.fa.params
-extra_res_cen HBI.cen.params
-loops:remodel perturb_kic
```

```
-loops:refine refine_kic
-kic_rama2b
-loops:ramp_fa_rep
-loops:ramp_rama
-loops:refine_outer_cycles 5
-kic_omega_sampling
-allow_omega_move true
-ex1
-ex2
-loops:loop_file loopdef.loop
-overwrite
```

Example loop definition file:

```
95 112 112 0 1
```

Supplementary Note 3.

Python script to design combinatorial library of linkers for grafting one EF-hand motif onto loop7 of mFAP2b.

The script requires installation of the biopython python module:

```
conda install -c bioconda biopython=1.72
```

To download the script as a Jupyter Notebook (`Supplementary_Note_3.ipynb`):

```
git clone https://github.com/klimaj/mFAPs.git
```

Supplementary Note 4.

PyRosetta script used to design circularly permuted mFAP2a and mFAP2b.

The serialization build of PyRosetta must be compiled to run the script. To download the python script (`Supplementary_Note_4.py`):

```
git clone https://github.com/klimaj/mFAPs.git
```

Supplementary References:

1. Mandell, D. J., Coutsias, E. A. & Kortemme, T. Sub-angstrom accuracy in protein loop reconstruction by robotics-inspired conformational sampling. *Nat. Methods* **6**, 551–552 (2009).
2. Stein, A. & Kortemme, T. Improvements to robotics-inspired conformational sampling in rosetta. *PLoS One* **8**, e63090 (2013).
3. Qian, B. *et al.* High-resolution structure prediction and the crystallographic phase problem. *Nature* **450**, 259–264 (2007).
4. Wang, C., Bradley, P. & Baker, D. Protein-protein docking with backbone flexibility. *J. Mol. Biol.* **373**, 503–519 (2007).
5. Alford, R. F. *et al.* The Rosetta All-Atom Energy Function for Macromolecular Modeling and Design. *J. Chem. Theory Comput.* **13**, 3031–3048 (2017).
6. Siedlecka, M., Goch, G., Ejchart, A., Sticht, H. & Bierzynski, A. Alpha-helix nucleation by a calcium-binding peptide loop. *Proc. Natl. Acad. Sci. U. S. A.* **96**, 903–908 (1999).
7. Olsson, L. L. & Sjölin, L. Structure of Escherichia coli fragment TR2C from calmodulin to 1.7 Å resolution. *Acta Crystallogr. D Biol. Crystallogr.* **57**, 664–669 (2001).
8. Fallon, J. L. & Quiocho, F. A. A closed compact structure of native Ca(2+)-calmodulin. *Structure* **11**, 1303–1307 (2003).
9. Ye, Y. *et al.* A grafting approach to obtain site-specific metal-binding properties of EF-hand proteins. *Protein Eng.* **16**, 429–434 (2003).
10. Zondlo, S. C., Gao, F. & Zondlo, N. J. Design of an encodable tyrosine kinase-inducible domain: detection of tyrosine kinase activity by terbium luminescence. *J. Am. Chem. Soc.* **132**, 5619–5621 (2010).
11. Tebo, A. G. *et al.* Circularly Permuted Fluorogenic Proteins for the Design of Modular Biosensors. *ACS Chem. Biol.* **13**, 2392–2397 (2018).
12. Fleishman, S. J. *et al.* RosettaScripts: a scripting language interface to the Rosetta macromolecular modeling suite. *PLoS One* **6**, e20161 (2011).
