# Supplementary material for: Incorporation of sensing modalities into de novo designed fluorescence-activating proteins
Source: Nat Commun. 2021 Feb 8;12:856. doi: 10.1038/s41467-020-18911-w (PMC7870846; doi:10.1038/s41467-020-18911-w)
Supplement: Supplementary file 19 — Reporting Summary [file 41467_2020_18911_MOESM19_ESM.pdf]

nature research

Corresponding author(s): David Baker

Last updated by author(s): Aug 4, 2020

Reporting Summary

Nature Research wishes to improve the reproducibility of the work that we publish. This form provides structure for consistency and transparency in reporting. For further information on Nature Research policies, see Authors & Referees and the Editorial Policy Checklist .

Statistics

For all statistical analyses, confirm that the following items are present in the figure legend, table legend, main text, or Methods section.

n/a	Confirmed	
☐	☒	The exact sample size (*n*) for each experimental group/condition, given as a discrete number and unit of measurement
☐	☒	A statement on whether measurements were taken from distinct samples or whether the same sample was measured repeatedly
☐	☒	The statistical test(s) used AND whether they are one- or two-sided<br>*Only common tests should be described solely by name; describe more complex techniques in the Methods section.*
☐	☒	A description of all covariates tested
☐	☒	A description of any assumptions or corrections, such as tests of normality and adjustment for multiple comparisons
☐	☒	A full description of the statistical parameters including central tendency (e.g. means) or other basic estimates (e.g. regression coefficient) AND variation (e.g. standard deviation) or associated estimates of uncertainty (e.g. confidence intervals)
☐	☒	For null hypothesis testing, the test statistic (e.g. *F*, *t*, *r*) with confidence intervals, effect sizes, degrees of freedom and *P* value noted<br>*Give P values as exact values whenever suitable.*
☐	☒	For Bayesian analysis, information on the choice of priors and Markov chain Monte Carlo settings
☐	☒	For hierarchical and complex designs, identification of the appropriate level for tests and full reporting of outcomes
☐	☒	Estimates of effect sizes (e.g. Cohen's *d*, Pearson's *r*), indicating how they were calculated

Our web collection on statistics for biologists contains articles on many of the points above.

Software and code

Policy information about availability of computer code

Data collection

- For designing extended loop7 decoys, the Rosetta build used was: v2018.12-dev60119
- The Rosetta loopmodel application (loopmodel.hdf5.linuxgccrelease) build used was: v2018.22-dev60238
- For performing superpositions and RMSD calculations, the PyRosetta conda build used was: 2019.01+HEAD.dbc838b6ae6
- For designing combinatorial libraries, the Rosetta versions used were: 2018.11.post.dev+93.master.5d3c24d; 2018.12+HEAD.5ecebca
- For designing combinatorial libraries, the Rosetta FoldIt standalone build used was: 20170206-84b2a4a6ef-osx_x86-INTERNAL
- The Synergy Neo2 hybrid multi-mode reader (BioTek) used Gen5 version 3.03.14.
- Densitometry was performed with Image Lab Software Version 6.0.1 build 34 Standard Edition (Bio-Rad).
- Laser scanning microscope LSM-510 used Release Version 4.2 SP1 software (Zeiss).
- Molecular dynamics simulations used: Schrodinger Maestro version 10.4; Dowser version 1.1; Amber version 14; Gaussian 09, revision C.01.
- Widefield epifluorescence microscopy acquisition of COS-7 cell images used: Micro-Manager version 1.4; MM Studio version 1.4.23. 20160628; MMCore version 8.3.0; Device API version 67; Module API version 10.
- Widefield epifluorescence microscopy acquisition of HEK293 cells images used: MetaMorph version 7.10.0.119.

Data analysis

- Molecular dynamics simulations used: VMD version 1.9.3; pyEMMA version 2.5.2; CPPTRAJ version 15; MDTraj version 1.9.3.
- Size-exclusion chromatography with multi-angle light scattering used: ASTRA version 7.2.
- SwiftLib used: online version [http://rosettadesign.med.unc.edu/SwiftLib/] accessed 2018-2019.
- Scale bars were added to LSM-510 images using LSM Image Examiner software version 4,0,0,241 (Zeiss).
- Absolute quantum yield data was analyzed by the built-in software U6039-05 PLQY measurement software Version 3.2.1 (Hamamatsu Photonics, Ltd., Shizuoka, Japan).
- X-ray crystallography data were analyzed with: HKL2000 v716.1; Phenix Phaser v2.7.16; Phenix Suite v1.11.1; Coot v0.8.9.
- Two-sided Wilcoxon sum rank was calculated in MATLAB version R2018b.
- Calculation of ΔF/F0 for HEK293 cell regions of interest was performed in Excel 2016.
- Figure 5d,e inset panels were rendered in Fiji (ImageJ) version 2.0.0-rc-69/1.52n.

- Figure 5f inset panel, Supplementary Figure 19a, and HEK293 cell regions of interest were hand-drawn and rendered with Fiji (ImageJ) version 2.0.0-rc-68/1.52g; java 1.8.0_66.
- Supplementary Movies 1 and 2, and fluorescence and brightfield images in Supplementary Figure 4, were rendered in Fiji (ImageJ) version 1.51 g.
- Relative quantum yield measurements were analyzed in Microsoft Excel 2013.
- A Savitzky-Golay filter of polyorder 3 was used to smooth fluorescence excitation and emission spectra.
- Open-source python modules installed into conda environments used for data analysis are available at: https://github.com/klimaj/mFAPs/tree/master/environments/

For manuscripts utilizing custom algorithms or software that are central to the research but not yet described in published literature, software must be made available to editors/reviewers. We strongly encourage code deposition in a community repository (e.g. GitHub). See the Nature Research guidelines for submitting code & software for further information.

Data

Policy information about availability of data

All manuscripts must include a data availability statement. This statement should provide the following information, where applicable:
- Accession codes, unique identifiers, or web links for publicly available datasets
- A list of figures that have associated raw data
- A description of any restrictions on data availability

The atomic coordinates and experimental data of the EF1p2_mFAP2b–DFHBI–Ca2+ co-crystal structure have been deposited in the RCSB Protein Data Bank with the accession code 6OHH. Time-lapse widefield epifluorescence microscopy movies of live COS-7 cells are available in Supplementary Movie 1 and Supplementary Movie 2. The normalized time-lapse widefield epifluorescence microscopy movie of live hiPSC-derived CMs expressing SR-targeted EF1n_mFAP2b labeled at 3.00 µM DFHBI is available in Supplementary Movie 3. Amino acid sequences of mFAP variants are reported in Supplementary Data 1 and Supplementary Data 2. Other amino acid, DNA and oligonucleotide sequences used throughout this research are reported in Supplementary Data 3 through Supplementary Data 17. Plasmid DNA that support the findings of this study are available at Addgene [www.addgene.org]. Source data (Figure 1f,g,h,i, Figure 2, Figure 5d,e,f, Supplementary Figure 1b,c, Supplementary Figure 3c, Supplementary Figure 4, Supplementary Figure 8a,b, Supplementary Figure 19b,c, and Supplementary Figure 23) are available online [https://doi.org/10.5281/zenodo.3960743]. Other plasmid DNA and data that support the findings of this study are available from the corresponding author upon reasonable request. Computational models for mFAP2a, mFAP2b, mFAP10, the 59 extended loop decoys, 5 refined extended loop7 decoys, 8 circularly permuted mFAP2a or mFAP2b decoys, and 12 circularly permuted mFAP2a or mFAP2b decoys with designed linkers are available to download; the β-barrel loop fragment databases used to design the extended loop library, Supplementary Table 1, and Supplementary Table 2 are available to download [https://github.com/klimaj/mFAPs].

Field-specific reporting

Please select the one below that is the best fit for your research. If you are not sure, read the appropriate sections before making your selection.

☒ Life sciences ☐ Behavioural & social sciences ☐ Ecological, evolutionary & environmental sciences

For a reference copy of the document with all sections, see nature.com/documents/nr-reporting-summary-flat.pdf

Life sciences study design

All studies must disclose on these points even when the disclosure is negative.

Sample size	Individual E. coli libraries were functionally screened to approximately 98% coverage (i.e. the number of E. coli colonies picked was approximately 4-fold the theoretical diversity of each library), which was considered adequate for experimental validation of each library (Engqvist MK, McIsaac RS, Dollinger P, et al. Directed evolution of Gloeobacter violaceus rhodopsin spectral properties. J Mol Biol. 2015;427(1):205-220. doi:10.1016/j.jmb.2014.06.015; Bosley AD, Ostermeier M. Mathematical expressions useful in the construction, description and evaluation of protein libraries. Biomol Eng 2005;22:57–61). For in cyto characterization of calcium-responsive mFAPs in HEK293 cells, sample-size calculations were not performed; 10-15 hand-drawn regions of interest was considered sufficient to capture the variability in cellular fluorescence responses (Supplementary Figure 18a,b,c,d). Technical and biological replicate sample sizes were considered sufficient based on the magnitude of measurable differences amongst samples.
Data exclusions	For analysis of photostability assays, pixel intensity values at the bit depth of the microscope detector (=4095) were discounted in each acquired frame because regions saturating the microscope detector do not capture photobleaching. This criterion was pre-established before image acquisition, and detector gain was adjusted between samples to minimize saturation of the microscope detector. Hand-drawn regions of interest (ROIs) of HEK293 cells for characterization of calcium-responsive mFAPs excluded cellular regions in each field of view. This criterion was pre-established prior to image acquisitions. For molecular dynamics simulations, the first 100 ns of each production run were discarded from analysis, which was established after running simulations and considered adequate to allow for structural relaxation from the starting conformations.
Replication	Individual E. coli libraries were screened once. Circular dichroism measurements, quantum yield measurements, X-ray crystallography, densitometry, epifluorescence microscopy of live and fixed COS-7 cells, HEK293 cell acetylcholine stimulations, cardiomyocyte imaging experiments, and size-exclusion chromatography with multi-angle light scattering were each performed once. Photostability assays, laser scanning confocal fluorescence microscopy of E. coli, HEK293 cell surface-displayed calcium titrations, size-exclusion chromatography, and in vitro chromophore, pH, calcium and split mFAP titrations were each replicated ≥2 times successfully. Variability was observed between purified protein samples in size-exclusion chromatography in terms of the relative fractions of monomeric/dimeric/aggregated proteins.
Randomization	Randomization was not performed because samples were defined by genetically-encoded amino acid sequence.

Blinding	Blinding was not performed because experiments were conducted by individual experimentalists, and was considered to not be relevant due to computer-based data analysis.

Reporting for specific materials, systems and methods

We require information from authors about some types of materials, experimental systems and methods used in many studies. Here, indicate whether each material, system or method listed is relevant to your study. If you are not sure if a list item applies to your research, read the appropriate section before selecting a response.

Materials & experimental systems

n/a	Involved in the study
☒	☐ Antibodies
☐	☒ Eukaryotic cell lines
☒	☐ Palaeontology
☒	☐ Animals and other organisms
☒	☐ Human research participants
☒	☐ Clinical data

Methods

n/a	Involved in the study
☒	☐ ChIP-seq
☒	☐ Flow cytometry
☒	☐ MRI-based neuroimaging

Eukaryotic cell lines

Policy information about cell lines

Cell line source(s)	HEK293 cell line source is from ATCC CRL-1573. COS-7 cell line source is from ATCC CRL-1651. IMR90 iPS cells (Clone #1) cell line source is from WiCell.
Authentication	HEK293 and COS-7 cell lines used were not authenticated upon receiving them. IM90 iPSCs were authenticated by short tandem repeat profiling performed by WiCell prior to acquirement.
Mycoplasma contamination	HEK293 cell lines tested negative for mycoplasma contamination upon receiving them. COS-7 cells tested negative for mycoplasma using 2-(4-Amidinophenyl)-6-indolecarbamidine dihydrochloride. IMR90 iPSCs were screened by WiCell prior to acquirement and tested negative for mycoplasma contamination.
Commonly misidentified lines (See ICLAC register)	Commonly misidentified lines were not used.
