# Supplementary material for: Incorporation of sensing modalities into de novo designed fluorescence-activating proteins
Source: Nat Commun. 2021 Feb 8;12:856. doi: 10.1038/s41467-020-18911-w (PMC7870846; doi:10.1038/s41467-020-18911-w)
Supplement: Supplementary file 20 — Description of Additional Supplementary Files [file 41467_2020_18911_MOESM20_ESM.pdf]

Title: Supplementary Movie 1.

Description: Time-lapse widefield epifluorescence microscopy of live COS-7 cell expressing mFAP2a targeted to the endoplasmic reticulum (ER) using a C-terminal fused sec61β localization sequence, labeled with 40.0 μM DFHBI. The time-lapse movie was acquired using 200 ms exposure times every 5 s for 25 total frames. The total acquisition duration was just over 2 min, and movie playback speed is 5 frames·s^{-1}. Excitation current was 100 mA.

Title: Supplementary Movie 2.

Description: Time-lapse widefield epifluorescence microscopy of live COS-7 cell expressing mFAP2b targeted to the ER using a C-terminal fused sec61β localization sequence, labeled with 40.0 μM DFHBI. The time-lapse movie was acquired using 200 ms exposure times every 5 s for 25 total frames. The total acquisition duration was just over 2 min, and movie playback speed is 5 frames·s^{-1}. Excitation current was 100 mA.

Title: Supplementary Movie 3.

Description: Time-lapse epifluorescence microscopy of human induced pluripotent stem cell (hiPSC)-derived cardiomyocytes (CMs) expressing sarcoplasmic reticulum (SR)-targeted EF1n_mFAP2b labeled at 3.00 μM DFHBI (Figure 5f). The average fluorescence pixel intensity per frame was computed, and the baseline of the average fluorescence pixel intensities approximated by iteratively fitting the baseline to a polynomial of degree 20. The approximate baseline average fluorescence pixel intensity per frame was subtracted from the average fluorescence pixel intensity per frame to compensate for photobleaching, then the baseline-subtracted average fluorescence pixel intensities were normalized across all frames scaling the

minimum peak baseline-subtracted average fluorescence pixel intensity to 1 and the maximum trough baseline-subtracted average fluorescence pixel intensity to 0. Raw fluorescence pixel intensities within each frame were normalized from 0 to 1, then re-scaled proportionally to the baseline-subtracted normalized average fluorescence pixel intensity, then pseudo-colored to show normalized fluorescence. The field of view contained 100% confluent cells, so regions without apparent hiPSC-derived CMs represent out-of-focus cellular SR fluorescence.

Title: Supplementary Data 1.

Description: mFAP, circularly permuted mFAP, split mFAP, and control amino acid sequences used in this study.

Title: Supplementary Data 2.

Description: Ca^{2+}-responsive mFAP amino acid sequences.

Title: Supplementary Data 3.

Description: Oligonucleotides and template DNA sequences used to construct the mFAP2.1 β-barrel site-directed mutagenesis (SDM) library.

Title: Supplementary Data 4.

Description: Oligonucleotides and template DNA sequences used to construct the mFAP2.2 β-barrel loop7 combinatorial library.

Title: Supplementary Data 5.

Description: Oligonucleotides and template DNA sequences used to construct the mFAP2.2 β-barrel core combinatorial library.

Title: Supplementary Data 6.

Description: Oligonucleotides and template DNA sequences used to construct the mFAP2.5 β-barrel methyl group combinatorial library.

Title: Supplementary Data 7.

Description: Oligonucleotides and template DNA sequences used to construct the mFAP2b β-barrel aromatics and aliphatics combinatorial library.

Title: Supplementary Data 8.

Description: Oligonucleotides and template DNA sequences used to construct the mFAP2a β-barrel aromatics and glycine combinatorial library.

Title: Supplementary Data 9.

Oligonucleotides and template DNA sequences used to construct the mFAP2a β-barrel "IN2" combinatorial library.

Title: Supplementary Data 10.

Description: Circular plasmid DNA sequence, gene sequences (human codon-optimized) and protein amino acid sequences used for transfection of cultured COS-7 cells.

Title: Supplementary Data 11.

Description: Oligonucleotides and plasmid DNA sequences used to construct the extended loop library for loop1, loop3, and loop7 of mFAP2b.

Title: Supplementary Data 12.

Description: Oligonucleotides and plasmid DNA sequences used to construct the combinatorial linker library to insert one EF-hand motif into loop7 of mFAP2b.

Title: Supplementary Data 13.

Description: Oligonucleotides and plasmid DNA sequences used to construct the combinatorial linker library to insert two EF-hand motifs into loop7 of mFAP2b.

Title: Supplementary Data 14.

Description: Oligonucleotides and plasmid DNA sequences used to construct the combinatorial linker library to insert four EF-hand motifs into loop7 of mFAP2b.

Title: Supplementary Data 15.

Description: Circular plasmid DNA sequence, gene sequences (human codon-optimized) and protein amino acid sequences of Ca^{2+}-responsive mFAP used for cell surface-displayed HEK293 cell Ca^{2+} titrations.

Title: Supplementary Data 16.

Description: Circular plasmid DNA sequence and gene sequence (human codon-optimized) of

Ca^{2+}-responsive mFAP used for rAAV6 production. The protein amino acid sequence expressed in human induced pluripotent stem cell-derived cardiomyocytes after viral infection is provided.

Title: Supplementary Data 17.

Description: Circular plasmid DNA sequence, gene sequence (human codon-optimized) and protein amino acid sequences of Ca^{2+}-responsive mFAPs used for HEK293 cell acetylcholine stimulations.
